# Pharmacological targeting of the mitochondrial phosphatase PTPMT1 sensitizes hepatocellular carcinoma to ferroptosis

**DOI:** 10.1038/s41419-025-07581-5

**Published:** 2025-04-06

**Authors:** Miaomiao Li, Yi Wang, Xinyan Li, Jiayi Xu, Liangwen Yan, Shenkang Tang, Chenyue Liu, Mengjiao Shi, Rongrong Liu, Yaping Zhao, Yi Zhang, Lan Yang, Yinggang Zhang, Gang Wang, Zongfang Li, Ying Guo, Yetong Feng, Pengfei Liu

**Affiliations:** 1 Department of Critical Care Medicine, National & Local Joint Engineering Research Center of Biodiagnosis and Biotherapy, The Second Affiliated Hospital of Xi’an Jiaotong University, Xi’an, China; 2 International Joint Research Center on Cell Stress and Disease Diagnosis and Therapy, National & Local Joint Engineering Research Center of Biodiagnosis and Biotherapy, The Second Affiliated Hospital of Xi’an Jiaotong University, Xi’an, China; 3 Department of Regenerative Medicine, School of Pharmaceutical Science, Jilin University, Changchun, China; 4 Department of Oncology, Affiliated Hospital of Shaanxi University of Chinese Medicine, Xianyang, China; 5 Department of Medical Image, The First Affiliated Hospital of Xi’an Jiaotong University, Xi’an, China; 6 Shaanxi Provincial Clinical Research Center for Hepatic & Splenic Diseases, The Second Affiliated Hospital of Xi’an Jiaotong University, Xi’an, China; 7 Key Laboratory of Surgical Critical Care and Life Support, Xi’an Jiaotong University, Ministry of Education of China, Xi’an, China; 8 Department of General Surgery, National & Local Joint Engineering Research Center of Biodiagnosis and Biotherapy, The Second Affiliated Hospital of Xi’an Jiaotong University, Xi’an, China; 9 Core Research Laboratory, The Second Affiliated Hospital of Xi’an Jiaotong University, Xi’an, China; 10 Key Laboratory of Environment and Genes Related To Diseases, Xi’an Jiaotong University, Ministry of Education of China, Xi’an, China

**Keywords:** Pharmacology, Cancer metabolism

## Abstract

Protein tyrosine phosphatase mitochondrial 1 (PTPMT1), is a member of the protein tyrosine phosphatase superfamily localized on the mitochondrial inner membrane, and regulates the biosynthesis of cardiolipin. Given the important position of PTPMT1 in mitochondrial function and metabolism, pharmacological targeting of PTPMT1 is considered a promising manner in disease treatments. In this study, we mainly investigated the role of PTPMT1 in hepatocellular carcinoma (HCC) ferroptosis, a new type of cell death accompanied by significant iron accumulation and lipid peroxidation. Herein, the pharmacological inhibition of PTPMT1 was induced by alexidine dihydrochloride (AD, a dibiguanide compound). Human HCC cell lines with PTPMT1 knockout and PTPMT1 overexpression were established using CRISPR/Cas9 and lentiviral transduction methods, respectively. The position of PTPMT1 in regulating HCC ferroptosis was evaluated in vitro and in vivo. Our results indicated that pharmacological inhibition of PTPMT1, facilitated by AD treatment, heightens the susceptibility of HCC to cystine deprivation-ferroptosis, and AD treatment promoted the conversion from ferritin-bound Fe^3+^ to free Fe^2+^, which contributed to the labile iron pool in cytoplasm. Meanwhile, pharmacological inhibition of PTPMT1 also induced the formation of both swollen mitochondria and donut mitochondria, and enhanced the metabolism process form succinate to fumarate in mitochondrial tricarboxylic acid (TCA) cycle, which increased the sensitivity of HCC cells to cystine deprivation-induced ferroptosis. In total, our work reveals the close association of PTPMT1 with cysteine deprivation-induced ferroptosis, providing a novel insight into chemotherapy strategies against human HCC.

## Introduction

PTPMT1 is the first protein tyrosine phosphatase found on the inner membrane of mitochondria. Some researchers have demonstrated that PTPMT1 is essential for the biosynthesis of cardiolipin, which is abundant in the inner mitochondrial membrane and necessary for normal respiratory chain enzyme activity [1, 2]. PTPMT1 deficiency influences mitochondrial respiration and leads to abnormal mitochondrial morphology. Moreover, a whole-body knockout PTPMT1 causes obvious embryonic lethality in mouse model, indicating the indispensable position of PTPMT1 during embryo development [2–4]. The function of PTPMT1 in metabolism regulation has also been confirmed in recent years. For example, mitochondrial metabolism is regulated by PTPMT1, and mitochondrial uncoupling protein 2 (UCP2) can be activated by the phosphatidylinositol phosphate substrates of PTPMT1 directly during the process of hematopoietic stem cell differentiation [5]. Another study also indicated that succinate dehydrogenase (SDH) serves as a substrate of PTPMT1, and the inhibition of PTPMT1 enhances the hyperphosphorylation and activation of SDH, suggesting the potential of PTPMT1 in glucose homeostasis [6]. Based on the important position of PTPMT1 in mitochondrial function and metabolism, pharmacological targeting of PTPMT1 has been considered a promising manner in disease treatments [7–9]. Currently, AD, a dibiguanide compound, is the only effective and selective inhibitor of PTPMT1 both in vitro and in vivo, and has been applied in cancer treatments, such as hepatocellular carcinoma [10] and pancreatic cancer [8]. In addition, PTPMT1 suppression also shows significant therapeutic action against breast cancer [11] and lung cancer [12]. Therefore, PTPMT1 is regarded as a novel target for cancer chemotherapy currently.

HCC is the most common form of primary liver cancer. It’s also one of the most prevalent and deadly cancers all over the world. The risk of HCC is higher in patients with hepatitis B/C infection or other long-term liver diseases. Human HCC is usually diagnosed at an advanced stage, and is characterized by a high resistance of chemotherapeutic drugs, resulting in limited chemotherapeutic efficacy and relapse after treatment [13–15]. Thus, effective strategies to overcome drug resistance are essential in anti-HCC therapeutics. Emerging evidence highlights the key position of ferroptosis, a new type of cell death accompanied by significant iron accumulation and lipid peroxidation, in regulating therapeutic responses of cancer cells. As a potential target for overcoming chemotherapy resistance, inducing ferroptosis reverses chemotherapeutic drug resistance in cancers, and understanding the mechanisms related to regulation of ferroptosis sensitivity is crucial to promote clinical application of ferroptosis modulators [16, 17]. Given that cellular redox contents are primarily controlled by glutathione (GSH), one of the most potent antioxidants, the suppression of cystine-glutamate antiporter (system Xc^-^, comprised of solute carrier family 7 member 11 (SLC7A11) and solute carrier family 3 member 2 (SLC3A2)) disrupts redox homeostasis and leads to ferroptosis via reducing cellular cysteine and GSH levels. Besides, GSH is considered as a necessary cofactor for the function of glutathione peroxidase 4 (GPX4), an antioxidant enzyme quenching phospholipid hydroperoxide. Thereby, GPX4 holds a central role in limiting lipid peroxidation and has been recognized as ferroptosis gatekeeper [18, 19]. Additionally, ferroptosis suppressor protein 1 (FSP1) is identified as a repressor of ferroptosis via regulating the production of Coenzyme Q10 (CoQ10), which is parallel to the GSH-dependent GPX4 pathway [20, 21]. Recently, some researchers have further underscored the importance of dihydroorotate dehydrogenase (DHODH) in regulating ferroptosis. DHODH reduces ubiquinone to ubiquinol, which is a radical-trapping antioxidant against ferroptosis. The function is independent of cytosolic GPX4 or FSP1, but parallel to mitochondrial GPX4 [22]. Similarly, phospholipid-modifying enzymes MBOAT1/2, which are transcriptionally upregulated by sex hormones and sex hormone receptors, suppress cell ferroptosis via remodeling the cellular phospholipid profile, and the effect is also independent of GPX4 or FSP1, highlighting the complexity of ferroptosis defense mechanisms [23]. Moreover, lots of components in the ferroptosis cascade are identified as target genes of the transcription factor nuclear factor erythroid 2-related factor 2 (NRF2), such as HMOX1, SLC7A11, and FSP1 [24–26], suggesting the potential of NRF2 mediators in the regulation of ferroptotic response as well as the treatment of ferroptosis-driven diseases.

The interplay between ferroptosis and mitochondria has garnered attention from several research groups, yet the precise role of mitochondria in ferroptosis regulation remains a topic of debate. Inhibition of either the mitochondrial TCA cycle or electron transfer chain (ETC) has been shown to mitigate lipid peroxide accumulation and, consequently, ferroptosis [22, 27, 28]. Furthermore, modulating the activity of mitochondrial TCA cycle and ETC, either by targeting specific mitochondrial enzymes or the anti-oxidant system, has been demonstrated to influence the susceptibility of cancer cells to ferroptosis [29, 30]. Interestingly, while mitochondria play a pivotal role in cysteine deprivation-induced ferroptosis, they appear to be less involved in ferroptosis triggered by GPX4 inhibition. Inhibition of canonical mitochondrial metabolic activities, such as the TCA cycle and ETC, alleviates ferroptosis induced by agents like erastin (a system Xc^−^ inhibitor) or conditions like cystine deprivation [29–31]. Given the integral role of PTPMT1 in mitochondrial function and metabolism, its pharmacological targeting emerges as a potential avenue to modulate ferroptosis sensitivity, thereby enhancing the efficacy of cancer chemotherapy. In this study, we focused on the role of PTPMT1 in modulating HCC ferroptosis. Our findings primarily suggest that pharmacological inhibition of PTPMT1, facilitated by AD treatment, heightens the susceptibility of HCC to ferroptosis through alterations in the mitochondrial TCA cycle and the labile iron pool. This underscores the potential of targeting PTPMT1 as a promising strategy in HCC chemotherapy.

## Material and methods

All animal experiments were conducted in strict compliance with ethical standards and were approved by the Biomedical Ethics Committee of the Health Science Center of Xi’an Jiaotong University (Approval number: 2022–1371) on June 9th 2022. These experiments adhered to the principles set forth in the Declaration of Helsinki, which guides the ethical conduct of research and is the framework upon which the Biomedical Ethics Committee of Health Science Center of Xi’an Jiaotong University bases its decisions.

### Chemicals and cell culture

Erastin (HY-15763), Sorafenib (HY-10201), RSL3 (HY-100218A), Alexidine dihydrochloride (AD, HY-108547), iFSP1 (HY-136057), Dimethyl malonate (DMM, HY-136057), Chloroquine (HY-17589A), MG-132 (HY-13259), and Cycloheximide (HY-12320) were sourced from MCE. Hepatocellular carcinoma cell lines, Hep 3B and Hepa1-6, were obtained from the American Type Culture Collection (ATCC, USA). MHCC97H was acquired from the China Center for Type Culture Collection (China), while Bel-7404 was procured from the Cell Bank of the Chinese Academy of Sciences (China). In this study, the normal liver cell line, BNL CL.2, was sourced from the National Collection of Authenticated Cell Cultures (China). All of the cell lines were maintained in DMEM (High Glucose) supplemented with 10% FBS, 100 U/mL penicillin, and 0.1 g/mL streptomycin, and incubated at 37 °C in a humidified atmosphere containing 5% CO_2_. The culture medium was refreshed every 2 days, and cells were passaged approximately every 3 to 4 days. For the establishment of a PTPMT1-knockout cell line, the CRISPR/Cas9 method, as described in our previous studies [32], was employed. The sgRNA sequences (5′-3′) used are as follows:

Human *PTPMT1*-sgRNA-A: 5′-TGAACGAGGAGTACGAGACG-3’

Human *PTPMT1*-sgRNA-B: 5′-GGTGCACAAATGGAGTCCAG-3’

### Cell viability assay

In our investigation, cell viability across various groups was assessed using the Cell Counting Kit-8 (CCK-8, Dojindo, Japan). Briefly, 10 μl of CCK-8 reagent was introduced to each well of a 96-well microplate, each containing 100 μl of medium. The microplate was then incubated at 37 °C for a duration of 4 h. Subsequently, the optical density (OD) values at 450 nm were recorded for each group (*n* = 3). Cell viability was calculated using the formula: Cell viability = (OD of experimental group − OD of blank group)/(OD of Ctrl group − OD of blank group). The cell viability for the Ctrl group (without any treatment) was set as a reference at “100%”, and the relative cell viability for the remaining groups was determined in comparison.

Furthermore, to evaluate cell death in different groups, samples were treated with propidium iodide (PI) staining solution (40710ES03, YEASEN) and incubated for 15 min. Subsequently, they were washed with phosphate-buffered saline (PBS) for three times. The stained cells from each group were then visualized and captured using a fluorescence microscope (Zeiss).

### Real-time qRT-PCR

Real-time qRT-PCR was executed in accordance with our previously established methodologies [29, 33]. Within this context, GAPDH was utilized for qPCR normalization, and all experiments were conducted in triplicate. The primer sequences (5’-3’) employed for the Real-time qRT-PCR reactions are as follows:

*PTGS2*-Forward 5’-CGGTGAAACTCTGGCTAGACAG-3’

*PTGS2*-Reverse 5’-GCAAACCGTAGATGCTCAGGGA-3’

*GAPDH*-Forward 5’-CTGACTTCAACAGCGACACC-3’

*GAPDH*-Reverse 5’-TGCTGTAGCCAAATTCGTTGT-3’

### Western blot

In this study, the western blot assay was conducted in alignment with our previously described methodologies [34, 35]. Concisely, protein samples (20 µg/lane) were separated utilizing either 8% or 12% SDS-PAGE gels and subsequently transferred to PVDF membranes. The membrane was blocked with 5% Nonfat-Dried Milk, followed by an overnight incubation with primary antibodies at 4 °C. In this study, the primary antibodies employed were as follows: anti-GPX4 (1:1000; Proteintech, 67763-1-Ig), anti-SLC7A11 (1:1000; Proteintech, 26864-1-AP), anti-FSP1 (1:1000; Proteintech, 68049-1-Ig), anti-DHODH (1:2000; Proteintech, 14877-1-AP), anti-TUBULIN (1:3000; Proteintech, 11224-1-AP), anti-FTL (1:2000; Proteintech, 10727-1-AP), anti-ATG5 (1:2000; Proteintech, 10181-2-AP), anti-BECLIN1 (1:2000; Proteintech, 11306-1-AP), anti-p62 (1:1000; Proteintech, 18420-1-AP), anti-LC3 (1:1000; Proteintech, 14600-1-AP), anti-MFN1 (1:2000; Proteintech, 13798-1-AP), anti-TOM20 (1:2000; Proteintech, 11802-1-AP), anti-PINK (1:2000; Proteintech, 23274-1-AP), anti-PGC-1α (1:2000; Proteintech, 66369-1-Ig), anti-SDHA (1:2000; Proteintech, 14865-1-AP), anti-NRF2 (1:1000; Proteintech, 16396-1-AP), anti-CASPASE3 (1:1000; ABclonal, A2156), anti-BCL2 (1:1000; ABclonal, A0208) and anti-GAPDH (1:3000; Proteintech, 10494-1-AP). Following incubation with HRP-labeled secondary antibodies (ABclonal), protein bands were visualized utilizing an enhanced chemiluminescence kit (SuperSignal West Femto Maximum Sensitivity Substrate, Thermo Fisher) and ChemiDoc Imagers (Bio-Rad Laboratories, USA).

### Evaluation of malondialdehyde (MDA)

In this study, MDA levels across various groups were measured to evaluate the extent of ferroptosis. MDA concentrations within cell lysates were assessed utilizing the Lipid Peroxidation (MDA) Assay Kit (MAK085, Sigma–Aldrich, USA), in strict accordance with the manufacturer’s instructions.

### BODIPY staining and intracellular iron measurement

For the assessment of BODIPY staining and intracellular iron content, cancer cells were first rinsed with PBS. They were then incubated with 1 μM BODIPY 581/591 C11 (D3861, Thermo Fisher) and FerroOrange solution (F374, Dojindo) for 30 min at 37 °C, respectively. Following the incubation, cells were washed twice with PBS. Subsequently, cell samples from each group were analyzed using the FACSAria II Flow Cytometer (BD Biosciences). Data interpretation was conducted using the FlowJo 7.6.1 software.

### Protein half-life assay

The assessment of protein half-life was conducted in a manner consistent with our prior research [36, 37]. In brief, cells were either maintained untreated or exposed to 1 μM Alexidine dihydrochloride for a duration of 24 h. Concurrently, 30 μM cycloheximide (CHX) was introduced to the culture medium to inhibit de novo protein synthesis. Cell samples were collected at designated time intervals and subjected to immunoblot analysis. The relative intensity of PTPMT1 in comparison to GAPDH was quantified utilizing the ImageJ software.

### TCA cycle assay

To assess the TCA cycle activity across different groups, key metabolites, namely α-ketoglutarate (α-KG), succinate (Suc), and fumarate (Fum), were quantified. In this study, α-KG concentrations were determined using the α-Ketoglutarate Assay Kit (MAK054, Sigma), while Suc levels were ascertained with the Succinate Colorimetric Assay Kit (MAK184, Sigma), both in strict accordance with the manufacturer’s protocols. Furthermore, Fum concentrations were evaluated using Fumarate Detection Kit (ab102516, Abcam). The enzymatic activity of succinate dehydrogenase (SDH) was gauged using SDH Assay Kit (BC0955, Solarbio).

### RNA sequencing and proteomics assay

In this study, total RNA was extracted from both AD-treated and untreated HCC cell replicates using TRIzol reagent. mRNA was subsequently isolated from the total RNA samples using poly-T oligo-attached magnetic beads. Sequencing libraries for the different groups were prepared using the NEBNext® Ultra™ RNA Library Prep Kit for Illumina® (NEB), strictly adhering to the manufacturer’s protocol. The prepared libraries were then sequenced on an Illumina Novaseq 6000 platform, facilitated by Novogene Beijing, China. In addition, the proteomics assay in our work was completed by Cosmos Wisdom, China.

### Measurement of mitochondrial membrane potential (MMP)

For the assessment of MMP, we utilized the Mitochondrial membrane potential assay kit (C2006, Beyotime). Briefly, cells from various experimental groups were incubated with JC-1 solution for a duration of 30 min, followed by washing with PBS. Subsequently, the MMP levels across the groups were quantified using a FACSAria II Flow Cytometer (BD Biosciences). Data analysis was conducted using FlowJo 7.6.1 software.

### Mitochondria and lysosomes staining

The localization of mitochondria and lysosomes was assessed utilizing Mito-Tracker Green (C1048, Beyotime) and Lyso-Tracker Red (C1046, Beyotime) staining, respectively. Briefly, cell samples from distinct experimental groups were incubated with 0.1 μM Mito-Tracker Green and 0.05 μM Lyso-Tracker Red staining solutions for a period of 30 min, followed by a wash with PBS. Subsequently, cellular imaging was performed using a Confocal Laser Scanning Microscope (Zeiss, Germany). In addition, the mitochondrial morphology was further evaluated under transmission electron microscope (TEM) as described [38].

### Ubiquitination assay

Herein, cells were co-transfected with overexpression vectors for Flag-PTPMT1 and HA-Ub for 24 h. To avoid AD-induced strong degradation PTPMT1, the transfected cells were treated with AD (1 μM and 2 μM) and MG132 for 4 h, then harvested using lysis buffer as our previous work [36, 37]. The target protein was pulled down using anti-Flag beads, and ubiquitination of PTPMT1 was evaluated using western blot finally.

### Immunoprecipitation assay

In the Immunoprecipitation Assay, cells were transfected with Flag-tagged PTPMT1 and Amcyan 1-tagged FTL, then were treated with AD (1 μM) for 4 h. Finally, cell lysates were collected in radioimmunoprecipitation assay (RIPA) buffer. The immunoprecipitated complexes were pulled down using anti-Flag beads. The protein samples in different groups were resolved by SDS-PAGE gel and subjected to immunoblot assay at last.

### Live cell immunofluorescence microscopy

In our study, the ptf-LC3 vector (mRFP-GFP-LC3 reporter construct) was used for Live Cell Immunofluorescence. Hep 3B cells were transfected with ptf-LC3 vectors for 16 h using Lipofectamine 3000 (Thermo Fisher Scientific). Then, the cells were treated with AD (1 μM) for 24 h. Finally, both treated and untreated cells were imaged using a fluorescence microscope (Zeiss).

### Xenograft mouse model

NOG mice, sourced from Charles River Laboratories, were utilized for the study. The 6-week-old male mice (weight = 18–22 g) were inoculated with cancer cell suspension containing 1 × 10^7^ cells/mouse (i.h.). Subsequently, these mice were randomly categorized into four experimental groups: Ctrl, AD, Era, and AD+Era. Tumor dimensions were ascertained using a vernier caliper, with the volume calculated as: (Volume = π/6 × Length × Width^2^). Both AD (0.25 mg/kg) and Era (15 mg/kg) were prepared in a 5% DMSO/corn oil solution and administered intraperitoneally to the mice twice weekly for a duration of 5 weeks. Following this treatment period, the mice were euthanized, and the tumor weight and TCA cycle metrics were evaluated for each group (*n* = 5).

### Statistical analysis

In this study, data are expressed as mean ± SD, Statistical analyses were conducted using SPSS 17.0. For comparisons between two distinct groups, unpaired Student’s *t*-tests were employed, while one-way ANOVA followed by Bonferroni’s post-hoc correction was utilized for comparisons among three or more groups. The Student’s *t*-test was executed as a one-tailed test, with a *p* value of less than 0.05 deemed statistically significant.

## Results

### Pharmacological inhibition of PTPMT1 sensitizes HCC to cystine deprivation-induced ferroptosis

**Fig. 1:**
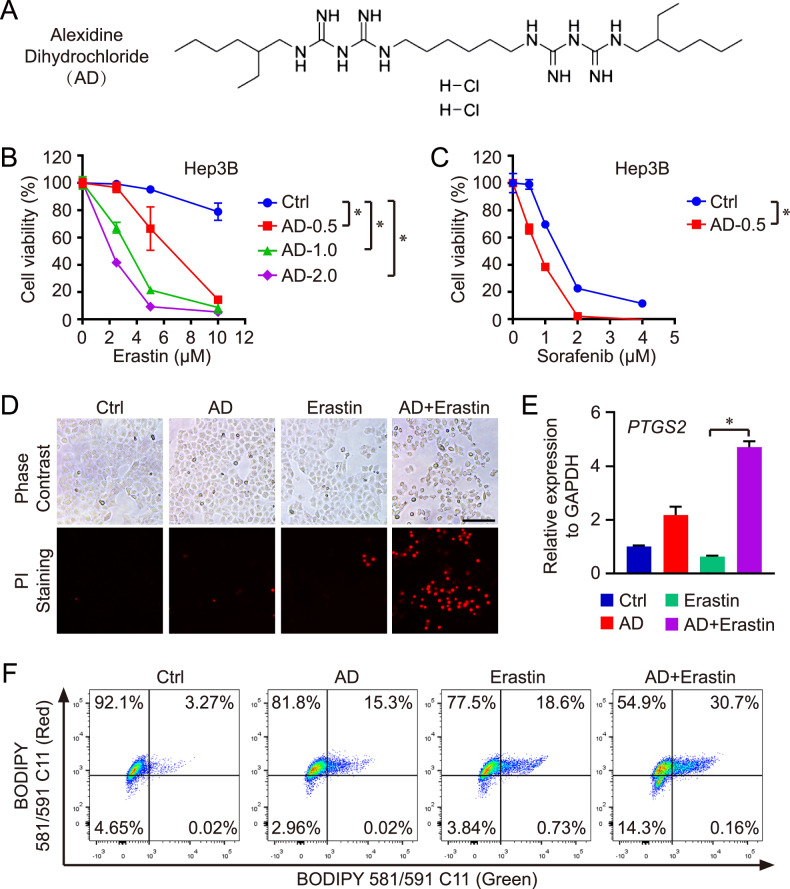
Pharmacological inhibition of PTPMT1 sensitizes HCC to ferroptosis. Pharmacological inhibition of PTPMT1 in Hep 3B cells was achieved through treatment with alexidine dihydrochloride (AD, the chemical structure is shown in (**A**)), while cystine deprivation-induced ferroptosis was induced using Erastin and Sorafenib for 24 h. Subsequently, cell viability in each group was assessed via the CCK-8 assay (**B**, **C**). Additionally, cell death in various treatment groups (AD (1 μM) and Erastin (10 μM)) was confirmed using PI staining (**D**). Scale bar = 50 μm. To analyze the ferroptosis level, the expression of *PTGS2* (**E**) and lipid peroxidation (**F**) were measured using qRT-PCR and BODIPY staining, respectively. Results are expressed as mean ± SD, and the *P* value less than 0.05 was considered statistically significant. *:*P* < 0.05 compared between two groups.

AD (Fig. 1A), as the sole effective and selective inhibitor of PTPMT1, was employed to pharmacologically inhibit PTPMT1 in HCC. Initially, the system Xc− inhibitor, Erastin, was administered to induce ferroptosis across various HCC cell lines. Our findings suggested that the pharmacological inhibition of PTPMT1 heightened the susceptibility of HCC cells (Hep 3B, BEL-7404, MHCC-97H, and Hepa1-6) to Erastin-mediated ferroptosis (Figs. 1B and S1A–E). Furthermore, we evaluated the response of AD-treated HCC cells to Sorafenib, which is also recognized as a ferroptosis inducer through cysteine depletion [39]. Similarly, the pharmacological inhibition of PTPMT1 markedly increased the sensitivity of HCC cells (Hep 3B and BEL-7404) and normal liver cells (BNL CL.2) to Sorafenib-mediated ferroptosis (Figs. 1C, and S1F, G). Nonetheless, in comparison to the untreated cohort, AD-treated HCC cells did not exhibit heightened sensitivity to RSL3 (GPX4 inhibitor)-induced cell death (Fig. S1H–J) as well as iFSP1 (FSP1 inhibitor)-induced cell death (Fig. S1K). This suggests that the pharmacological inhibition of PTPMT1 specifically augments HCC sensitivity to cystine deprivation-induced ferroptosis, but not to GPX4 or FSP1 inhibition-mediated ferroptosis.

Concurrently, cell mortality across all groups was assessed through PI staining. The expression of *PTGS2*, a maker gene of ferroptosis, and lipid peroxidation were quantified using qRT-PCR and BODIPY staining, respectively. Some recent reports have indicated that both apoptosis and ferroptosis are involved in Sorafenib-induced cell death. Differently, Erastin is regarded as a canonical ferroptosis inducer via suppressing the uptake of cystine mediated by SLC7A11 [40–42]. Therefore, Erastin was chosen for our further research. Our findings further substantiated that AD treatment augmented the susceptibility of HCC cells to Erastin-mediated cell death, underscoring the pivotal role of pharmacological inhibition of PTPMT1 in cystine deprivation-induced ferroptosis (Fig. 1D–F).

### AD-induced PTPMT1 inhibition contributes to the labile iron pool

**Fig. 2:**
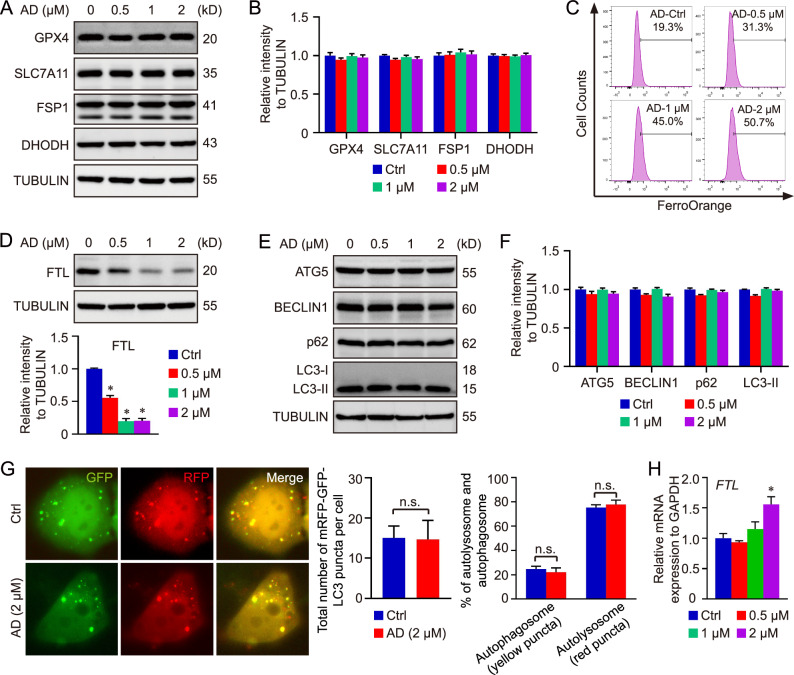
AD-induced PTPMT1 inhibition contributes to the labile iron pool. Hep 3B cells were exposed to AD at concentrations of 0, 0.5, 1, and 2 μM for 24 h. Then the cells were collected and subjected to immunoblotting to assess the expression of ferroptosis regulators (**A**, **B**). Concurrently, the intracellular concentration of free Fe^2+^ was quantified using FerroOrange staining (**C**). The protein abundance of ferritin light chain (FTL) was also evaluated across the treatment groups (**D**). Subsequently, the expression profiles of various autophagy makers were analyzed via western blotting (**E**, **F**), and the influence of AD treatment on autophagic flux was tested via live cell immunofluorescence assay (**G**). Finally, the transcription of FTL was evaluated in Hep 3B treated with AD at different concentrations (**H**). Data are presented as mean ± SD, and the *P* value less than 0.05 was considered statistically significant. *:*P* < 0.05 compared with Ctrl group.

To explore the mechanism related to PTPMT1 and ferroptosis, we subsequently examined the impact of AD treatment on various ferroptosis regulators. Our data revealed that the expression of neither GPX4 nor SLC7A11 was altered by AD treatment. Additionally, the protein levels of FSP1 and DHODH remained unaltered in AD-treated HCC cells compared to the control group (Fig. 2A, B). To validate these findings, PTPMT1 overexpression was induced via vector transfection, and the overexpression of PTPMT1 did not affect the principal ferroptosis regulators (GPX4, SLC7A11, NRF2, and DHODH) (Fig. S2A), highlighting the unique role of PTPMT1 in cellular ferroptosis. Concurrently, the level of Fe^2+^ was increased in HCC cells following AD treatment, accompanied by the downregulation of ferritin light chain (FTL) (Fig. 2C, D). Consequently, AD-induced pharmacological inhibition of PTPMT1 may facilitate the conversion from ferritin-bound Fe^3+^ to free Fe^2+^, contributing to the labile iron pool in the cytoplasm. Furthermore, ferritin degradation is primarily reliant on autophagy, referred to as ferritinophagy [17, 26, 43]. The impact of AD on autophagy was additionally assessed in HCC cells. However, the results indicated that AD treatment did not influence the protein levels of p62, BECLIN1, ATG5, and LC3, analogous to the effect of PTPMT1 overexpression (Figs. 2E, F and S2B). Moreover, the influence of AD on autophagic flux was further determined using the tandem mRFP-GFP-LC3 reporter construct. HCC cells were transfected with the construct and treated with AD (2 µM). The results indicated that either red puncta or yellow puncta were changed in AD-treated cells compared with the untreated cells, suggesting that AD treatment didn’t affect the formation of autophagosome or autolysosome in HCC cells (Fig. 2G). Thus, the AD-induced downregulation of FTL and the elevated level of Fe^2+^ are independent of autophagy, and it’s possible that AD treatment induces FTL downregulation via some other degradation pathway. In addition, we also tested the mRNA level of FTL, and the qRT-PCR results showed that AD-induced PTPMT1 inhibition didn’t suppress the transcription of FTL gene (Fig. 2H). The interplay between PTPMT1 and FTL was also determined via immunoprecipitation and immunofluorescence assay. As shown in Fig. S2C, D, PTPMT1 couldn’t directly bind with FTL. Therefore, the effect of AD treatment on FTL mainly depends on some indirect mechanism, which still needs further investigation.

### AD treatment augments the proteasomal degradation of PTPMT1 in HCC cells

**Fig. 3:**
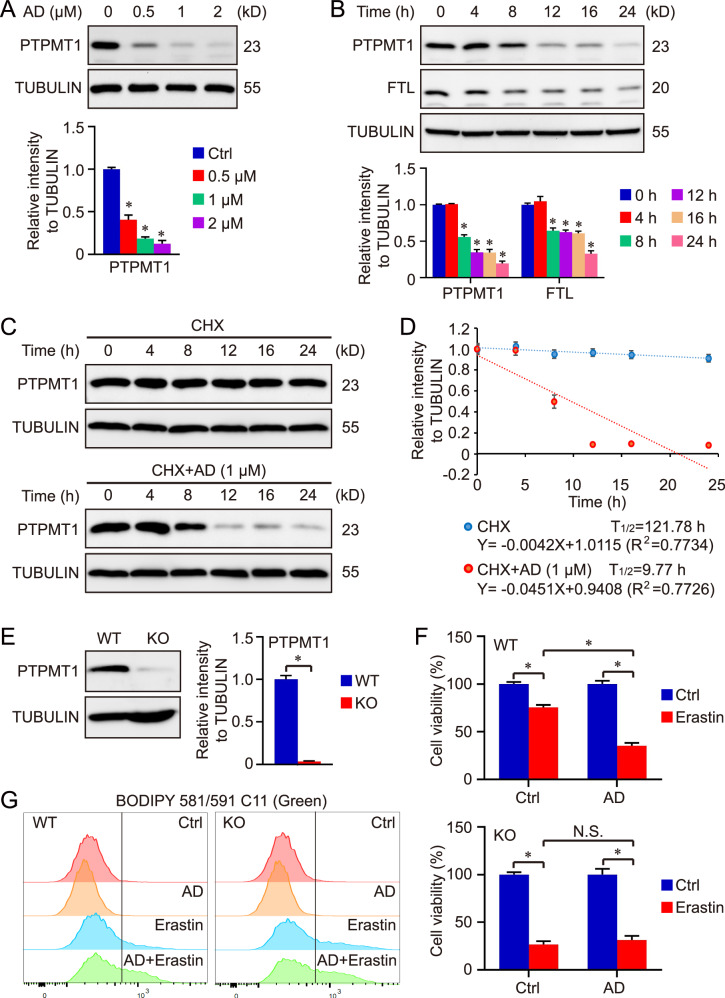
AD treatment augments the proteasomal degradation of PTPMT1 in HCC cells. Hep 3B cells underwent treatment with AD at concentrations of 0, 0.5, 1, and 2 μM for a duration of 24 h, after which they were collected for PTPMT1 assay (**A**). Separately, cells exposed to 1 μM AD were harvested at distinct time intervals (0, 4, 8, 12, 16, and 24 h), and the protein expression of PTPMT1 and FTL was analyzed via western blotting (**B**). Data are depicted as mean ± SD. A *P* value below 0.05 was deemed statistically significant. *: *P* < 0.05 relative to the Ctrl group. To ascertain the half-life of PTPMT1, HCC cells were treated with cycloheximide to inhibit protein synthesis, and PTPMT1 expression was assessed at various time points through western blotting (**C**). The intensity of the PTPMT1 band, normalized to TUBULIN, against time is illustrated in the subsequent plot (**D**). In this study, a PTPMT1 knockout cell line was established using the CRISPR/Cas9 system, with the knockout efficacy verified via immunoblotting (**E**). Furthermore, both wild type (WT) and PTPMT1 knockout (KO) cells were subjected to AD treatment, and the susceptibility of AD (1 μM)-treated cells to Erastin (10 μM)-induced ferroptosis (24 h) was assessed through cell viability assays (**F**) and BODIPY staining (**G**). *:*P* < 0.05 compared between the two groups.

Given that AD treatment did not influence cellular autophagy, it’s plausible that AD-induced PTPTM1 downregulation is also not related with autophagy-dependent degradation. Our data demonstrated that AD downregulated PTPMT1 in a dose-dependent manner (Fig. 3A). In addition, treatment with MG132, a proteasome inhibitor, elevated PTPMT1 protein levels in HCC cells, whereas Chloroquine (CQ, autophagy inhibitor) had no effect on PTPMT1 levels (Fig. S3A). Furthermore, MG132 treatment partially counteracted the inhibitory effect of AD on PTPMT1 (Fig. S3B), underscoring the modest function of ubiquitin-mediated proteasomal degradation in the AD-induced pharmacological inhibition of PTPMT1. To confirm the conclusion, the ubiquitylation state of PTPMT1 was further determined via an immunoprecipitation assay. As shown in Fig. S3C, a higher level of PTPMT1 ubiquitylation was observed in the group treated with AD at a concentration of 1 μM or 2 μM for 4 h. The treatment with AD (1 μM for 8 h) decreased the protein level of PTPMT1 in the vector transfection system (Fig. S3D). Therefore, AD promotes the proteasomal degradation of PTPMT1. However, the treatment with MG132 only partially rescued AD-induced PTPMT1 degradation (Fig. S3B), which indicated that other potential degradation pathways are involved in AD function. It’s possible that the ratio of ubiquitinated PTPMT1 is still too low after 4-h treatment with AD, which is hard to influence the total amount of PTPMT1 in HCC cells.

The degradation kinetics of PTPMT1 and FTL were subsequently assessed in AD-treated HCC cells, revealing that 4-hour treatment with AD didn’t affect the protein level of PTPMT1 or FTL, and the amount of PTPMT1 and FTL exhibited pronounced degradation after a 8-hour treatment at least (Fig. 3B). Additionally, PTPMT1 overexpression elevated FTL protein levels (Fig. S2A), indicating that AD-induced FTL downregulation may contingent upon PTPMT regulation. Nonetheless, the precise interplay between PTPMT1 and FTL warrants further investigation. Based on the impact of AD on proteasomal degradation of PTPMT1, it was hypothesized that the half-life of PTPMT1 might be decreased in AD-treated HCC cells. To validate this, HCC cells were exposed to CHX to inhibit protein synthesis, and PTPMT1 levels were monitored at various intervals. Compared with single CHX treatment, the co-treatment with AD and CHX showed enhanced degradation of PTPMT1 protein. As anticipated, AD treatment significantly reduced the half-life of PTPMT1 (Fig. 3C, D).

Subsequently, HCC cell lines with PTPMT1 knockout were established using CRISPR/Cas9 methods (Fig. 3E). To further elucidate the role of PTPMT1 in the function of AD, both the PTPMT1 knockout cell lines were exposed to AD and Erastin. The data revealed that AD treatment heightened the sensitivity of wild-type HCC cells to cystine deprivation-induced ferroptosis, while PTPMT1 knockout completely negated the AD-mediated effects on HCC ferroptosis (Fig. 3F). Lipid peroxidation across all groups was quantified using BODIPY staining. In alignment with prior findings, AD amplified Erastin-induced lipid peroxidation in wild-type HCC cells but not in PTPMT1 knockout cells (Fig. 3G). Moreover, the proliferation ability of both wild-type cells and PTPMT1 knockout cells was evaluated in our work. We noticed that the proliferation ability was suppressed by PTPMT1 knockout in HCC cells (Fig. S4A). Moreover, we also tested the function of AD on FTL expression in PTPMT1 knockout cells. The results showed that the protein level of FTL was downregulated by AD treatment in wild-type cells. Compared with wild-type cells, the level of FTL in PTPMT1 knockout cells was much lower. In addition, the suppressing function of AD on FTL was also abolished in PTPMT1 knockout cells, suggesting that AD-induced FTL downregulation could be dependent of PTPMT1 regulation (Fig. S4B). In addition, the effect of AD treatment on PTPMT1 transcription was further determined using qRT-PCR. As expected, the mRNA level of PTPMT1 gene wasn’t influenced by AD treatment in different concentrations (Fig. S4C).

### Pharmacological inhibition of PTPMT1 modulates genes pivotal for mitochondrial metabolism in HCC

**Fig. 4:**
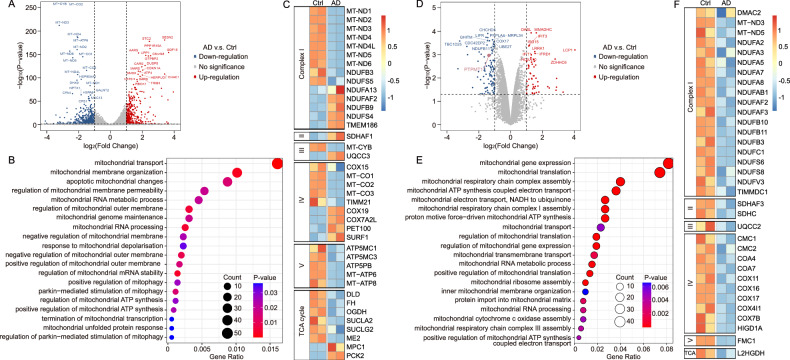
Pharmacological inhibition of PTPMT1 modulates genes pivotal for mitochondrial metabolism in HCC. To elucidate the potential signaling associated with AD treatment, Hep 3B cells were administered AD (1 μM) for 24 h, followed by harvesting for RNA-seq analysis. A volcano plot was generated to visualize differentially expressed genes between AD-treated and AD-untreated cells (**A**). Furthermore, differentially expressed genes were comprehensively summarized through Gene Ontology Enrichment Analysis (**B**), and genes pertinent to mitochondrial TCA cycle and ETC activity were collated in a heat map (**C**). To further confirm the function of AD, proteomics assay was performed along with RNA-seq assay. Similar to RNA-seq data, a volcano plot was generated to visualize differentially expressed genes between AD-treated and AD-untreated cells (**D**), and the differentially expressed genes were further summarized through Gene Ontology Enrichment Analysis (**E**). At last, genes pertinent to mitochondrial TCA cycle and ETC activity were organized in a heat map (**F**).

To elucidate the precise mechanisms underlying the impact of AD on ferroptosis, we conducted an RNA-seq assay to compare genome-wide mRNA expression profiles between AD-treated and untreated HCC cells. Following a 24-hour exposure, 447 genes were upregulated and 808 genes were downregulated in the AD-treated HCC cells relative to their untreated counterparts (Fig. 4A). Gene Ontology Enrichment Analysis revealed that the pharmacological inhibition of PTPMT1 by AD modulated a plethora of genes pivotal to mitochondrial metabolism and function, encompassing areas such as mitochondrial transport, mitochondrial membrane organization, mitochondrial RNA metabolic process (Fig. 4B). A heatmap of illustrating differential transcript levels between untreated and AD-treated cells underscored the central role of PTPMT1 in the mitochondrial TCA cycle and ETC activity (Fig. 4C). To further explore the pharmacological mechanism of AD, proteomics assay was performed in our work. The results indicated that the protein levels of 57 genes were upregulated and 104 genes were downregulated in the AD-treated HCC cells compared with untreated cells (Fig. 4D). Similar to RNA-seq results, the Gene Ontology Enrichment Analysis indicated that the pharmacological inhibition of PTPMT1 by AD affected the protein levels of genes pivotal to mitochondrial function, such as mitochondrial gene expression, mitochondrial translation and mitochondrial respiratory chain complex assembly (Fig. 4E). In addition, we also noticed that some functional proteins in mitochondrial TCA cycle and ETC activity were suppressed by AD treatment in HCC cells (Fig. 4F). Differently, the transcription of some genes (e.g. MPC1 and PCK2) were enhance by AD in RNA-seq data, while the protein levels of those genes didn’t show obvious difference between AD group and Ctrl group in proteomics analysis. Therefore, multi-omics assay is essential for us to fully understand the pharmacological mechanisms of AD. Additionally, it’s possible that AD-induced PTPMT1 degradation is also associated with mitochondria, and the accurate mechanism still needs further investigation. The raw data of RNA-seq and proteomics assay have been deposited in Entrez Molecular Sequence Database System (Citation accession: PRJNA1193844) and ProteomeXchange (Citation accession: PXD058606), respectively.

### AD treatment perturbs mitochondrial morphology and functionality

**Fig. 5:**
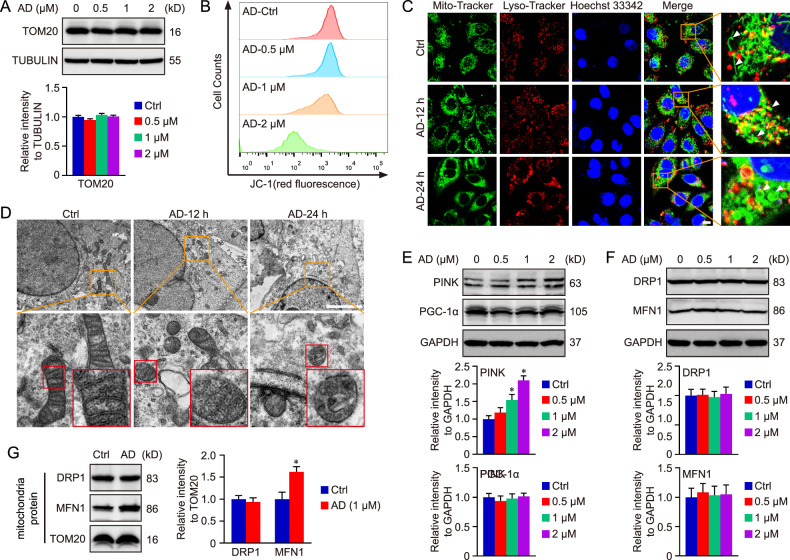
AD treatment perturbs mitochondrial morphology and functionality. Following exposure to AD at concentrations of 0, 0.5, 1, and 2 μM for 24 h, cells were collected for immunoblot analysis (**A**). Concurrently, the mitochondrial membrane potential (MMP) was assessed via JC-1 staining (**B**). In this study, the localization of mitochondria and lysosomes was ascertained using Mito-Tracker Green and Lyso-Tracker Red staining, respectively. The ramifications of 1 μM AD treatment on HCC cells were documented at specific intervals (0, 12, and 24 h) (**C**, scale bar = 10 μm), and the mitochondrial morphology was also evaluated via transmission electron microscopy assay (**D**, scale bar = 2 μm). In addition, mitophagy-associated protein PINK and mitochondrial biogenesis-associated protein PGC-1α were tested using western blot (**E**). Finally, mitochondrial fission-associated protein DRP1 and mitochondrial fusion-associated protein MFN1 were measured in total cell lysate and mitochondria, respectively (**F**, **G**). Data are presented as mean ± SD, and a *P* value less than 0.05 was considered statistically significant. *:*P* < 0.05 compared with Ctrl group.

Guided by RNA-seq and proteomics assay results, we subsequently assessed the impact of AD treatment on mitochondria within HCC cells. Initially, the total mitochondrial content was evaluated in HCC cells post-AD treatment. Our findings revealed that AD did not alter the protein level of TOM20, a constitutive protein in the protein import machinery of the mitochondrial outer membrane (Fig. 5A). Concurrently, mitochondrial content across all groups was quantified via Mito-Tracker staining. Similar to immunoblot results, Mito-Tracker signaling did not exhibit a notable alteration in HCC cells treated with varying concentrations of AD (Fig. S5A). Consequently, AD treatment-induced PTPMT1 inhibition did not affect the overall mitochondrial content in HCC cells. The mitochondrial membrane potential (MMP) was further assessed in each group utilizing JC-1 staining, revealing that AD administration diminished the MMP of HCC cells in a dose-dependent manner (Fig. 5B). Given that previous studies have unveiled the crosstalk between mitochondria and lysosomes [44, 45], we further explored the impact of AD on lysosomes in HCC. The data indicated that AD treatment did not exert a conspicuous influence on lysosome quantity nor on the co-localization of mitochondria (green) and lysosomes (yellow) (Figs. S5B, C, and 5C). However, both confocal microscopy assay (Fig. 5C) and transmission electron microscopy assay (Fig. 5D) showed that mitochondrial morphology transitioned from a linear to a swelling or donut shape following AD treatment. The impact of AD on mitophagy and mitochondrial biogenesis was assessed in HCC cells, and the results showed that AD treatment increased the level of mitophagy-associated protein (PINK), but did not influence the expression of PGC-1α, a mitochondrial biogenesis maker (Fig. 5E). In addition, mitochondria serve as dynamic organelles and engage in the coordinated cycles of fission and fusion. Therefore, both mitochondrial fission-associated protein DRP1 and mitochondrial fusion-associated protein MFN1 were measured to evaluate mitochondrial dynamics. We found that the levels of neither DRP1 nor MFN1 showed significant difference in the total cell lysate of AD-treated and AD-untreated cells (Fig. 5F). Differently, AD administration obviously increased the protein level of MFN1 in mitochondria but not in total lysate, indicating the impact of AD on mitochondrial fusion in HCC (Figs. 5G and S5D).

### Pharmacological targeting of PTPMT1 enhances the metabolic transition from succinate to fumarate in the mitochondrial TCA cycle

**Fig. 6:**
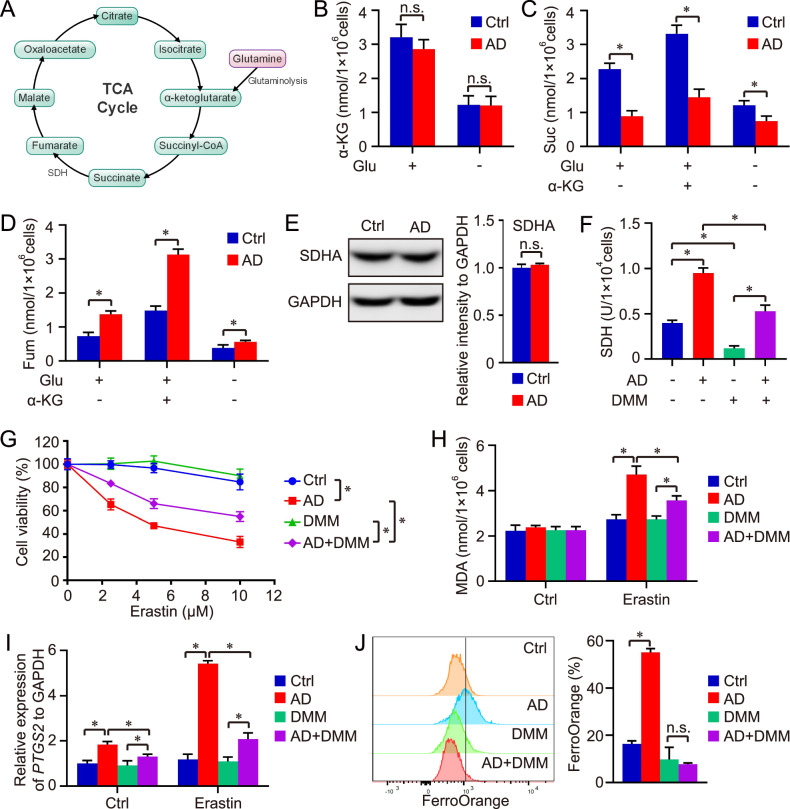
Pharmacological targeting of PTPMT1 enhances the metabolic transition from succinate to fumarate in mitochondrial TCA cycle. An overview of the mitochondrial TCA cycle is presented in (**A**). Cells treated with AD (1 μM) were co-administered with glutamine (Glu) deprivation or α-Ketoglutarate (α-KG, 5 mM) for 24 h. The levels of α-KG (**B**), succinate (Suc, **C**), and fumarate (Fum, **D**) were quantified within each group. Simultaneously, the protein levels of succinate dehydrogenase subunit A (SDHA) were determined by western blot (**E**), and the SDH activity was assessed in both AD-treated (1 μM for 24 h) and AD-untreated cells (**F**). Furthermore, dimethyl malonate (DMM, 5 mM) was applied in the ferroptosis model to corroborate the role of SDH in our study. Initially, cell viability was assessed in each group using CCK-8 (**G**). Subsequently, the levels of MDA (**H**) and the expression of *PTGS2* (**I**) were quantified using the Lipid Peroxidation (MDA) Assay Kit and Real-time qPCR, respectively, to ascertain the occurrence of ferroptosis in HCC. At last, the effect of DMM and AD treatment on free Fe^2+^ was further determined via FerroOrange staining (**J**). Data are presented as mean ± SD, and the *P* value less than 0.05 was considered statistically significant. *:*P* < 0.05 compared between two groups.

Numerous studies have underscored the pivotal role of the mitochondrial TCA cycle in regulating ferroptosis [22, 27, 28]. Consequently, we sought to elucidate the impact of AD-induced PTPMT1 inhibition on the mitochondrial TCA cycle (Fig. 6A). In this context, we initially quantified the downstream metabolites of glutaminolysis. Our findings revealed that AD treatment did not alter the basal levels of α-Ketoglutarate (α-KG) under normal conditions or in the absence of glutamine (Fig. 6B). However, succinate (Suc) levels diminished post-AD treatment. While α-KG supplementation elevated cellular Suc levels, the disparity in Suc levels between control and AD-treated groups became more pronounced (Fig. 6C). We then assessed fumarate (Fum), a downstream metabolite of Suc known to influence ferroptosis sensitivity [46]. Contrarily, AD treatment augmented Fum levels under various conditions (normal, α-KG supplementation, and glutamine absence), highlighting AD’s significant role in the conversion of Suc to Fum (Fig. 6D). Consequently, we examined succinate dehydrogenase (SDH), the enzyme mediating the Suc to Fum transition in the TCA cycle. Even though AD treatment did not alter SDH protein levels (Fig. 6E), the pharmacological inhibition of PTPMT1 by AD enhanced SDH activity, facilitating the metabolic conversion from Suc to Fum (Fig. 6F). SDH consists of four subunits: SDHA, SDHB, SDHC, and SDHD, which are encoded by different genes. Therefore, it’s hard to establish SDH knockout cell line. To further validate the centrality of SDH in AD’s modulation of ferroptosis, we employed dimethyl malonate (DMM), a potent SDH inhibitor [47], in our study. The treatment with DMM blocked AD-induced SDH activation significantly (Fig. 6F). Moreover, AD at low dose (1 μM) didn’t affect the MDA content in HCC cells, and single DMM treatment couldn’t affect MDA level as well. Under the condition of Erastin exposure, DMM rescued the influence of AD on HCC cell death, and reduced the sensitivity of AD-treated HCC cells to Erastin-induced ferroptosis (Fig. 6G, I). Besides, the effect of DMM on free Fe^2+^ was further determined via FerroOrange staining. Similar to previous results, AD-induced pharmacological inhibition of PTPMT1 increased the level of free Fe^2+^ and contributed to the labile iron pool in HCC. The function could be abolished by DMM treatment, indicating the key role of SDH in the regulation of AD on HCC ferroptosis (Fig. 6J).

**Fig. 7:**
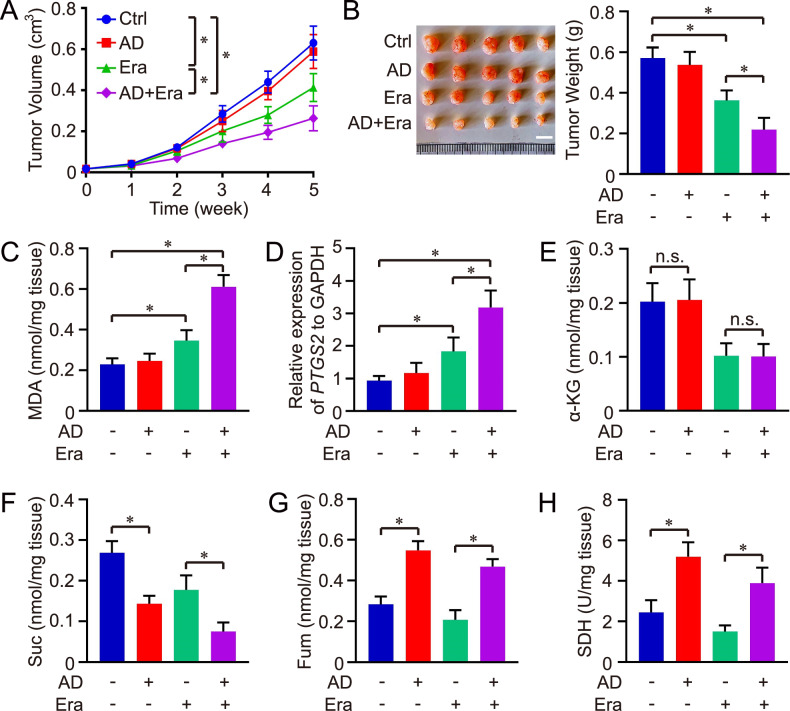
Pharmacological targeting of PTPMT1 amplifies anti-tumor efficacy of erastin in xenograft mouse model. Hep 3B cells were injected into NOG mice, which were subsequently treated with Erastin (Era) and AD. Tumor volume was monitored throughout the treatment period (**A**). Following a 5-week treatment regimen, mice were sacrificed, and tumor weights were quantified for each group (**B**). Concurrently, the level of MDA (**C**) and *PTGS2* expression (**D**) in tumor tissues was assessed using the Lipid Peroxidation (MDA) Assay Kit and qPCR, respectively, to evaluate ferroptosis. Lastly, the levels of α-KG (**E**), succinate (Suc, **F**), fumarate (Fum, **G**), and the activity of SDH (**H**) were measured across different groups. Results are expressed as mean ± SD, and the *P* value less than 0.05 was considered statistically significant. *:*P* < 0.05 compared between two groups.

Furthermore, we assessed the therapeutic efficacy of combining Erastin and AD against HCC in vivo. Notably, the combined regimen of Erastin and AD exhibited superior therapeutic outcomes compared to either agent alone, with both tumor volume and weight being significantly reduced in the combination group relative to the individual AD or Erastin groups (Fig. 7A, B). Echoing in vitro findings, the MDA levels and *PTGS2* expression were elevated in the combination group compared to the individual AD or Erastin groups, suggesting that pharmacological targeting of PTPMT1 enhances the sensitivity of HCC to cystine deprivation-induced ferroptosis in vivo (Fig. 7C, D). Moreover, the influence of AD on the mitochondrial TCA cycle was also investigated in HCC tissues. Our data revealed that AD treatment augmented SDH activity in HCC tissues and facilitated the metabolic transition from Suc to Fum, indicating the intricate interplay between PTPMT1 and the mitochondrial TCA cycle in the HCC ferroptosis model (Fig. 7E–H)

## Discussion

PTPMT1, a mitochondrial protein tyrosine phosphatase, is instrumental in modulating cardiac and skeletal muscle functions. For instance, PTPMT1 is pivotal for sustaining mitochondrial adaptability. Its deficiency disrupts mitochondrial carbohydrate and lipid utilization, culminating in muscle atrophy and heart failure [48]. Moreover, PTPMT1 can shield cardiomyocytes from γ-ray irradiation-induced necroptosis by mitigating mitochondrial damage [49]. Furthermore, PTPMT1 is integral to embryonic cardiac cardiolipin biosynthesis, which is determinative for mitochondrial morphogenesis and heart development [4]. Recently, the pharmacological targeting of PTPMT1 has emerged as a promising strategy in oncology, given the critical role of mitochondrial function and metabolism in tumorigenesis [10, 11]. Some researchers have identified that AD-mediated PTPMT1 inhibition instigates mitochondrial demise in pancreatic cancer cells through the modulation of the SLC25A6-NDUFS2 signaling pathway [8]. In our investigation, we corroborated the therapeutic potential of targeting PTPMT1 in HCC. Our findings demonstrated that AD treatments exceeding 2 μM exhibited pronounced cytotoxicity across various HCC cell lines (BEL-7404, MHCC-97H, Hepa1-6, and Hep 3B). In contrast, normal liver cells (BNL CL.2) and hepatic stellate cells (JS-1) manifested reduced susceptibility to AD-induced cytotoxicity, underscoring AD’s selective targeting in oncology (Fig. S6A, B). Concurrently, we observed that AD treatment augmented ROS production and intensified lipid peroxidation (Fig. S6C–E). Thus, high-dose AD-mediated pharmacological inhibition of PTPMT1 could effectively induce ferroptosis in HCC cells, even in the absence of other ferroptosis inducers. We also tested the level of cysteine as well as GSH in AD-treated cells. Our results indicated that AD treatment decreased the level of cysteine as well as GSH in a dose-dependent manner (Fig. S6F, G), indicating that cysteine metabolism is involved in PTPMT1 inhibition-induced ferroptosis. However, we noticed that the AD at a concentration of 1 μM only caused tiny decrease of cysteine, while AD at same concentration sensitized hepatocellular carcinoma to Erastin-mediated ferroptosis obviously (Figs. 1A, and S1A–E). Therefore, AD-caused cysteine downregulation might not play the major role in the regulation of HCC sensitivity to cystine deprivation-induced ferroptosis. In addition, our current work mainly investigated the effect of AD treatment on the sensitivity of HCC cells to ferroptosis. To avoid the toxicity of high-dose AD, the concentration of AD we used in xenograft model is much lower than the reference [10] and does not significantly inhibit the growth of xenograft tumors, so that it will be more convincing to evaluate the chemotherapy sensitization function of AD.

In this study, we primarily demonstrated that AD-mediated pharmacological inhibition of PTPMT1 heightened the sensitivity of HCC to ferroptosis by modulating the mitochondrial TCA cycle and the labile iron pool. AD treatment concomitantly reduced the protein levels of both PTPMT1 and FTL. Several researchers have highlighted the centrality of mitochondria in governing the labile iron pool. For instance, ROS derived from mitochondria facilitate ferritinophagy through the activation of the AMPK-ULK1 axis, thereby influencing the labile iron pool [50]. Additionally, autophagy sustains mitochondrial metabolism by orchestrating iron homeostasis [51]. Thus, it is plausible that AD’s impact on the labile iron pool is intricately linked to its regulation of mitochondrial metabolism. In alignment with this, our data revealed that FTL exhibited pronounced degradation after AD treatment (Fig. 3B) and PTPMT1 overexpression directly elevated FTL protein levels (Fig. S2A), signifying a dependent association between PTPMT1 and FTL. However, AD-induced PTPMT1 inhibition didn’t suppress the transcription of FTL gene (Fig. 2H), and PTPMT1 couldn’t directly bind with FTL in immunoprecipitation and immunofluorescence assay as well (Fig. S2C–D). Therefore, it’s possible that the effect of AD-induced PTPMT1 inhibition on FTL mainly depends on some indirect mechanism, and the precise mechanisms interlinking PTPMT1 inhibition and the labile iron pool warrant comprehensive exploration.

The intricate interplay between the mitochondrial TCA cycle and ferroptosis has been highlighted by numerous studies [29, 31, 52, 53]. yet the underlying mechanisms remain to be fully elucidated. The pivotal role of fumarate in ferroptosis regulation was first unveiled in 2018. Researchers discovered that C93 of GPX4 undergoes post-translational modification by fumarate, which inhibits GPX4 activity, thereby facilitating ferroptosis in hereditary leiomyomatosis and renal cell cancer [46, 54]. In our investigation, we discerned that AD-mediated pharmacological inhibition of PTPMT1 augments cysteine deprivation-induced ferroptosis by amplifying SDH activity, which in turn accelerates fumarate production in the mitochondrial TCA cycle. Intriguingly, AD treatment did not heighten the susceptibility of HCC cells to RSL3 (a GPX4 inhibitor)-induced cytotoxicity (Fig. S1H–J). It is conceivable that RSL3 and AD target analogous domains in GPX4, rendering AD treatment ineffective in further potentiating HCC’s sensitivity to RSL3-induced ferroptosis. In addition, the expression of multiple mitochondrial genes was suppressed by AD treatment. Based on the critical role of mitochondria in metabolic reprogramming, it’s possible various biological process, such as glucose mechanism, glycolysis, as well as fatty acid and amino acid oxidation are involved in AD influence on HCC sensitivity to ferroptosis. The complex regulatory networks underlying AD pharmacological function still need more detailed investigation.

In our study, we observed a shift in mitochondrial morphology from elongated to either swollen or donut-shaped configurations following AD treatment. Researchers have previously established distinctions between swollen and donut-shaped mitochondria, with swollen mitochondria being targeted for selective removal through autophagy, while donut-shaped mitochondria engage in fission and fusion cycles for potential reintegration. In their research, it was demonstrated that under conditions of serum starvation, mitochondria underwent distinct topological transformations, giving rise to swollen and donut-shaped mitochondria. MMP dissipation and PRKN recruitment played pivotal roles in the selective elimination of swollen mitochondria. In contrast, donut-shaped mitochondria maintained MMP and resisted mitophagy by suppressing the recruitment of autophagosome receptors CALCOCO2/NDP52 and OPTN [38]. Similarly, pharmacological suppression of the mitochondrial phosphatase PTPMT1 with AD also induced the formation of both swollen and donut-shaped mitochondria (Fig. 5C, D). However, AD treatment did not appear to affect cellular autophagy in HCC (Figs. 2E–G and S2B). Donut-shaped mitochondria have been observed in various tissues following events such as hypoxia-reoxygenation or changes in osmotic pressure [55–58]. Nevertheless, the precise functional roles of these distinct mitochondrial morphologies remain elusive. Importantly, further investigations are needed to determine which type of mitochondria contributes to the enhanced mitochondrial TCA cycle and sensitivity to ferroptosis.

Beyond its role in ferroptosis, several studies have highlighted the association between SRSF1-regulated PTPMT1 splice switching and cell apoptosis, both in vitro and in vivo [11]. In our study, we examined the impact of pharmacological inhibition of PTPMT1 on a Cisplatin-induced cell apoptosis model. Our findings indicated that AD treatment potentiated the vulnerability of HCC cells to Cisplatin-induced cytotoxicity. Concurrently, there was a dose-dependent decline in the expression of B-cell CLL/lymphoma 2 (BCL2), a known apoptosis inhibitor, in AD-treated HCC cells. This suggests a potential mechanism underpinning AD’s pro-apoptotic effect (Fig. S7A, B). Consequently, the pharmacological targeting of PTPMT1 with AD presents significant therapeutic potential across diverse cancer chemotherapy paradigms. In addition, the protein level of cysteinyl aspartate specific proteinase-3 (CASPASE3) wasn’t affected by AD treatment (Fig. S7B). Activated CASPASE3 degrades intracellular structural proteins as well as other functional proteins, and finally induces cell death. Therefore, the pro-apoptotic function of AD could be independent of CASPASE3 signaling pathway. Furthermore, PTPMT1 occupies a crucial role in immune cells, modulating immune responses effectively. For instance, the absence of PTPMT1 attenuates T cell responsiveness to antigens, attributed to the cardinal role of cardiolipin in T cell activation. Moreover, PTPMT1-mediated cardiolipin synthesis is indispensable during memory T cell differentiation [9]. Collectively, these insights position PTPMT1 as a promising therapeutic target in tumor immunotherapy.

**Fig. 8:**
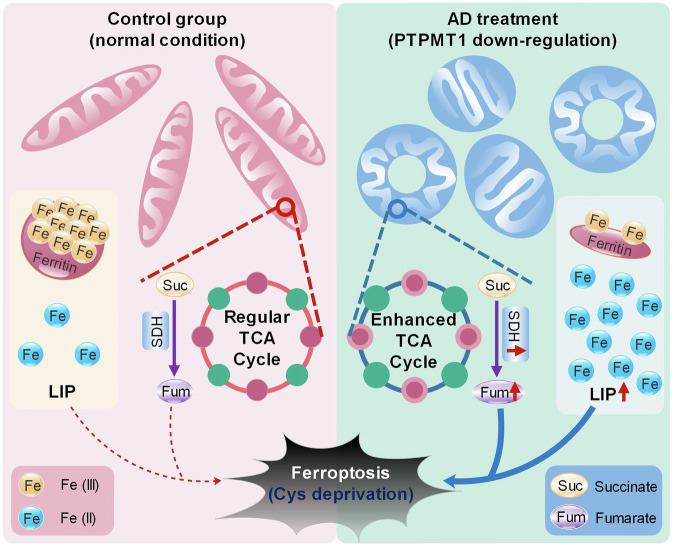
Proposed mechanistic model delineating the role of PTPMT1 in cysteine deprivation-induced ferroptosis. The pharmacological inhibition of PTPMT1 by alexidine dihydrochloride (AD) augments the susceptibility of HCC to ferroptosis. On one front, AD treatment facilitates the conversion of ferritin-bound Fe^3+^ to free Fe^2+^, enriching the labile iron pool (LIP) in the cytoplasm. Concurrently, pharmacological targeting of PTPMT1 not only induces the emergence of both swollen and donut-shaped mitochondria but also amplifies the metabolic transition from succinate (Suc) to fumarate (Fum) within the mitochondrial TCA cycle. Collectively, these alterations heighten the sensitivity of HCC cells to cystine deprivation-induced ferroptosis.

In conclusion, our research primarily elucidates the connection between PTPMT1 and ferroptosis in HCC. We determined that AD-mediated pharmacological inhibition of PTPMT1 heightens the susceptibility of HCC to cysteine deprivation-induced ferroptosis by modulating the labile iron pool and the mitochondrial TCA cycle (Fig. 8). While the intricate interplay between PTPMT1, the labile iron pool, and mitochondrial function warrants deeper exploration, our findings offer a promising perspective on therapeutic strategies against human HCC.

## Supplementary information

The uncropped raw images of western blot

Supplementary Materials

## Data Availability

The data are available from the corresponding author upon reasonable request and with the permission of the institution. In addition, both RNA and protein sequence data have been deposited in Entrez Molecular Sequence Database System (Citation accession: PRJNA1193844) and ProteomeXchange (Citation accession: PXD058606) respectively.
