# Supplementary material for: Pharmacological targeting of the mitochondrial phosphatase PTPMT1 sensitizes hepatocellular carcinoma to ferroptosis
Source: Cell Death Dis. 2025 Apr 6;16(1):257. doi: 10.1038/s41419-025-07581-5 (PMC11973169; doi:10.1038/s41419-025-07581-5)

The uncropped raw images of western blot

Figure 2A

GPX4

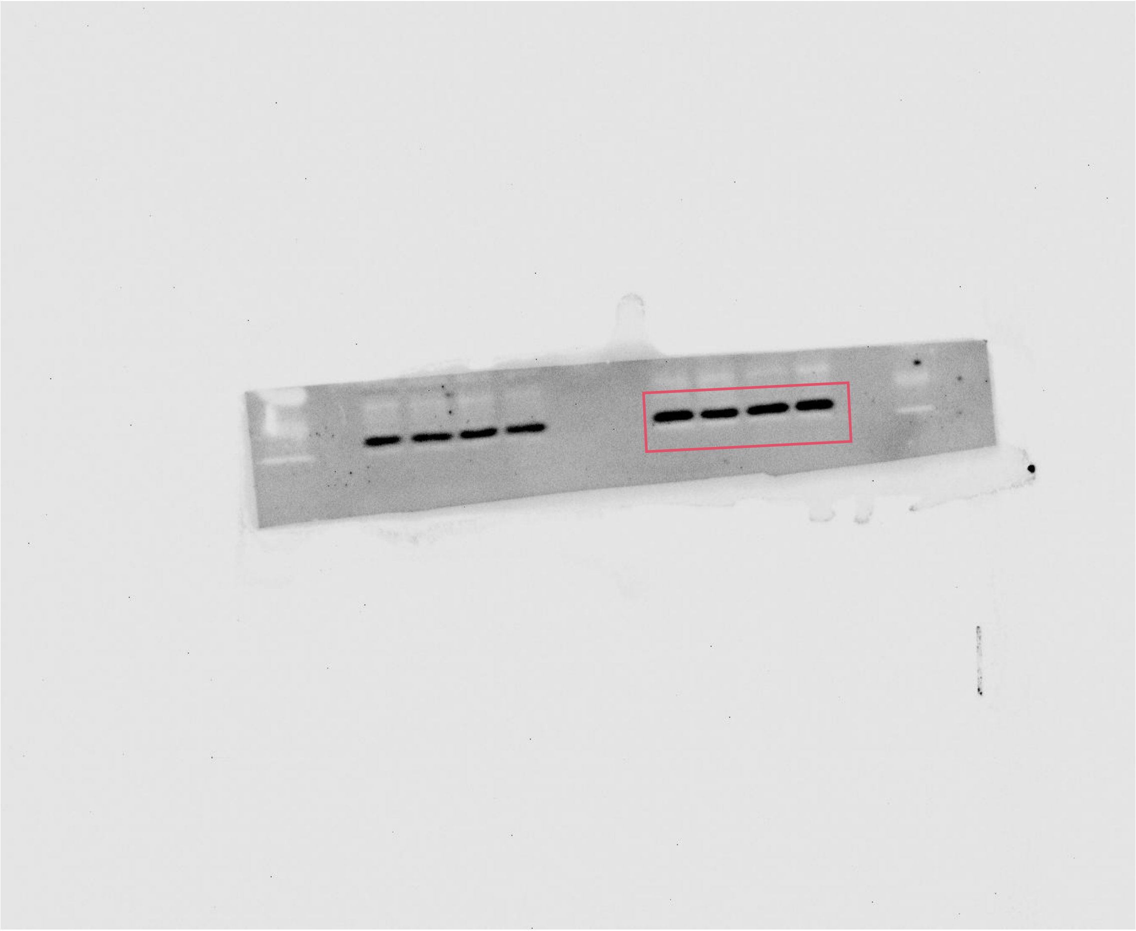

Figure 2A

SLC7A11

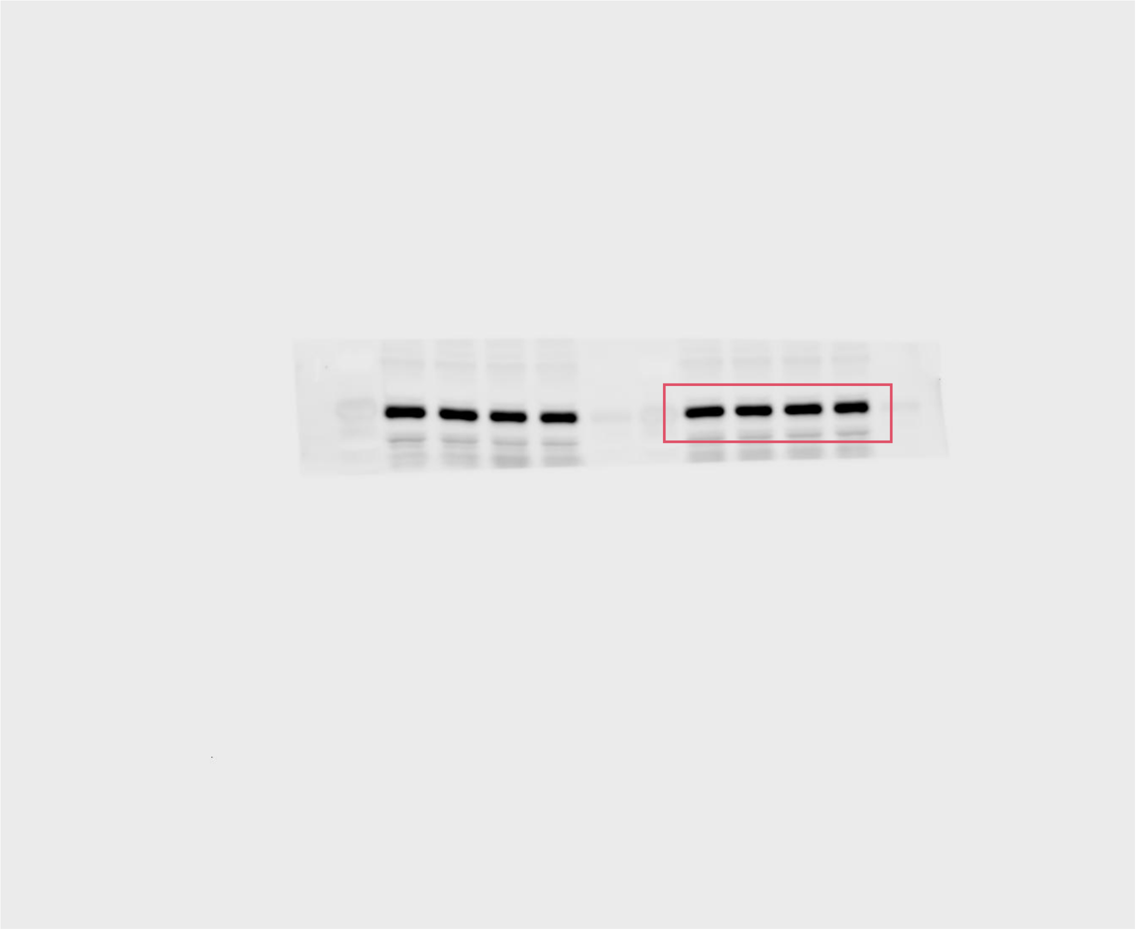

Figure 2A

FSP1

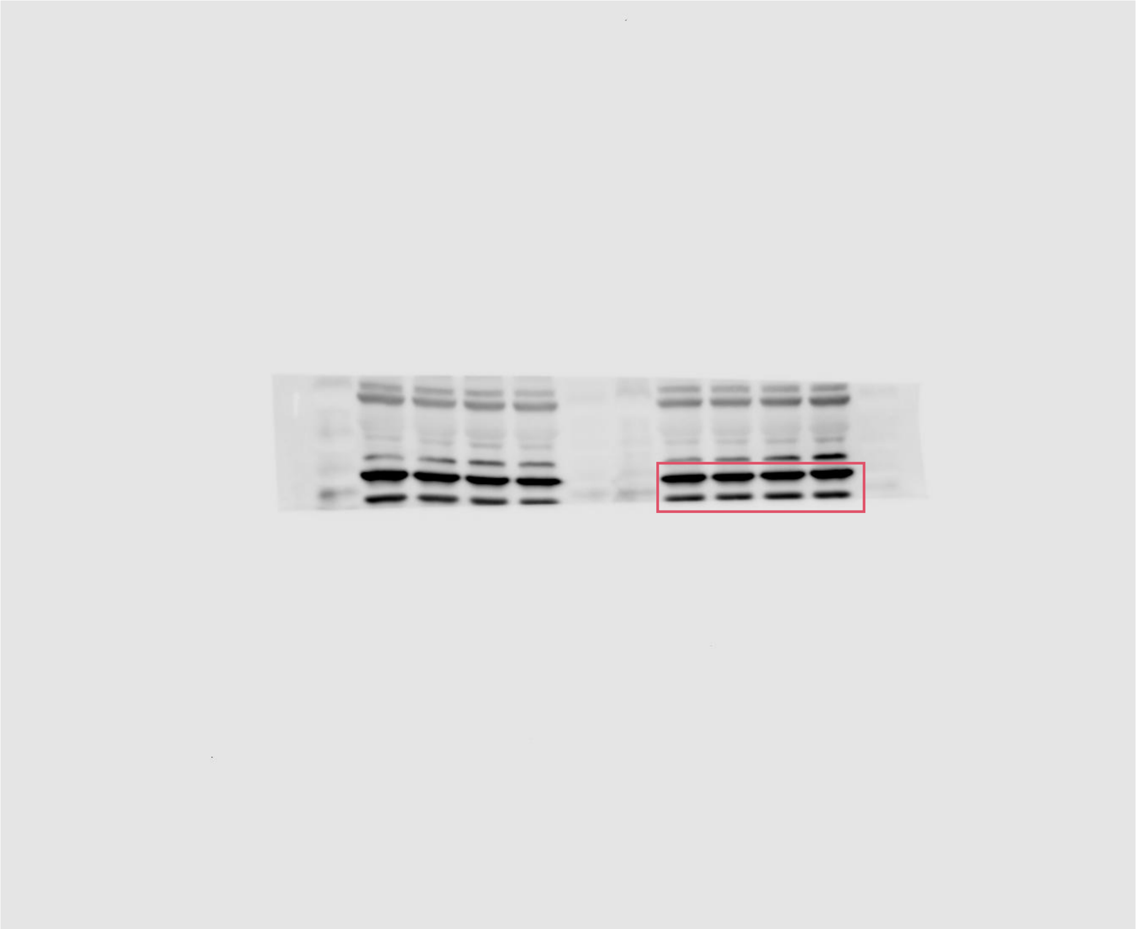

Figure 2A

DHODH

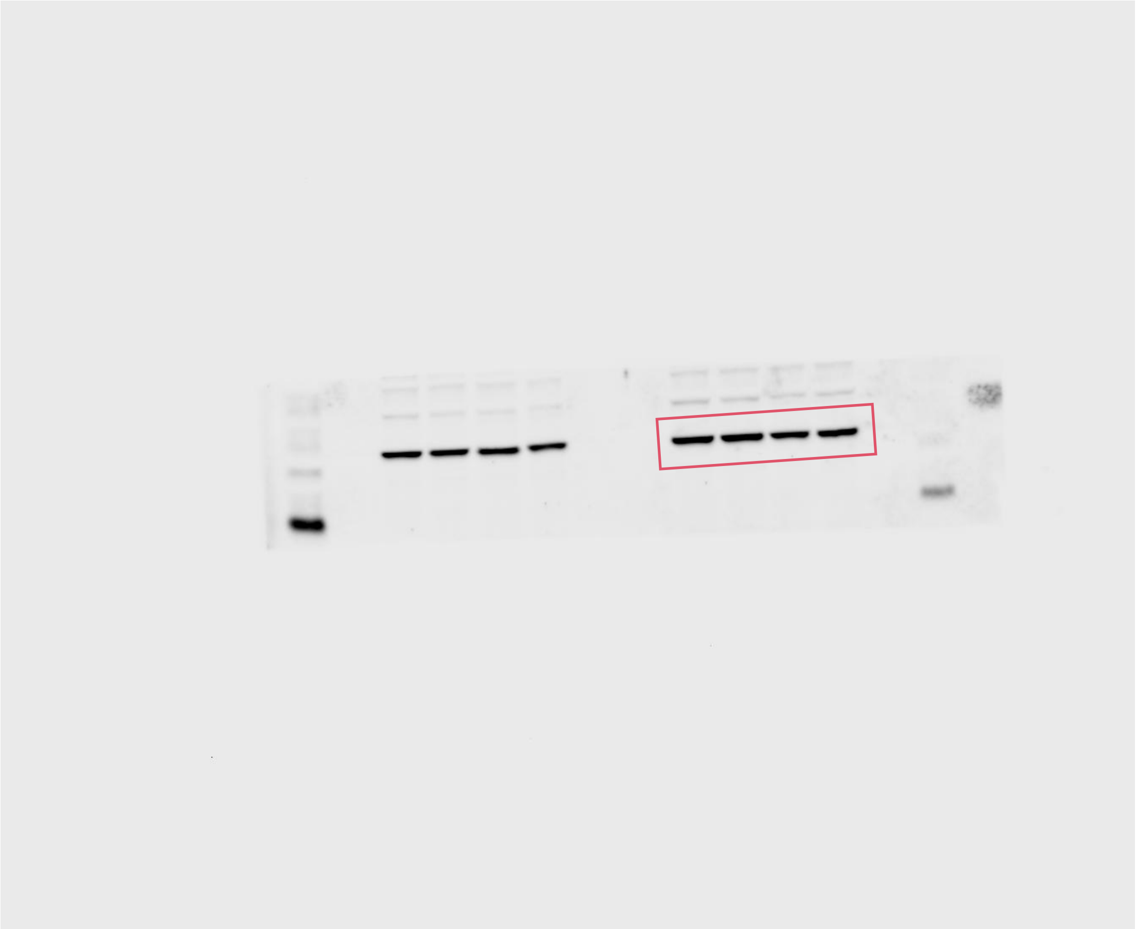

Figure 2A

TUBULIN

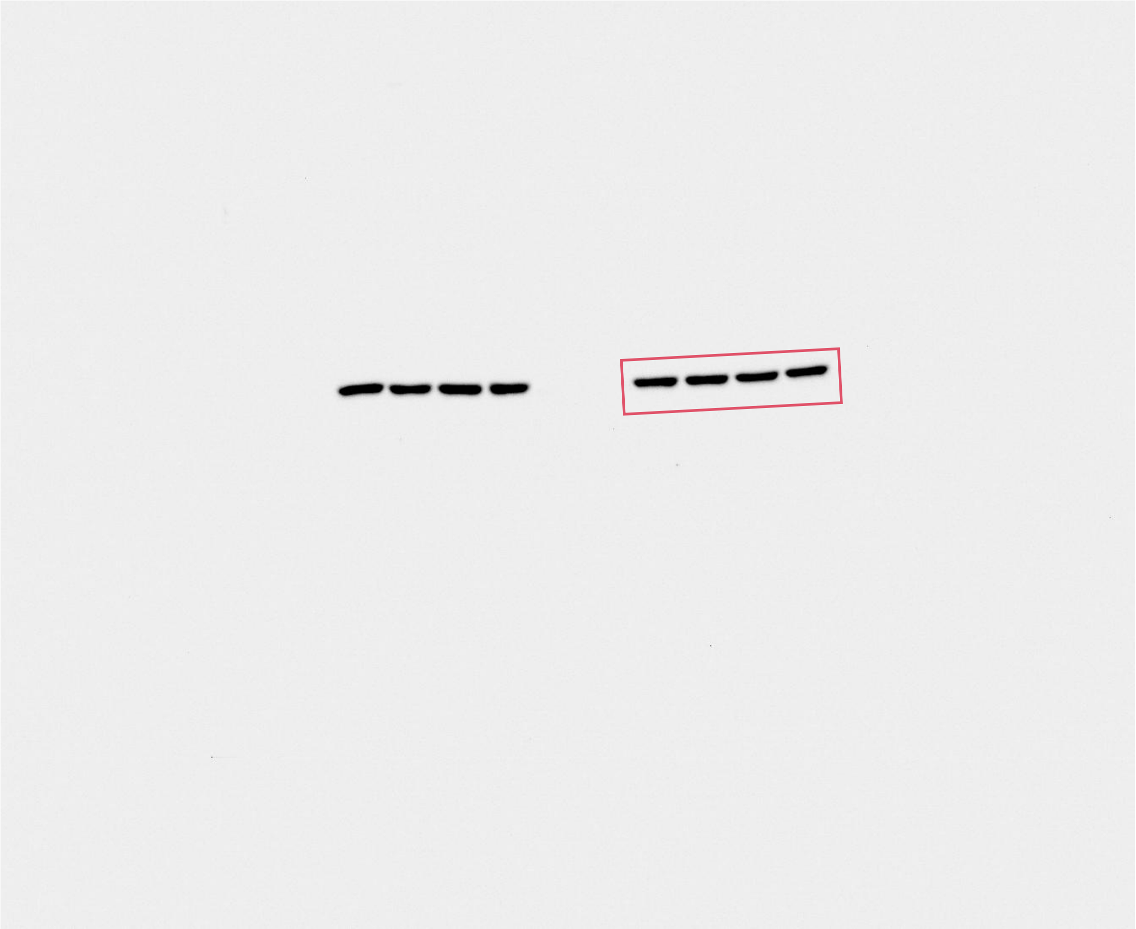

Figure 2D

FTL

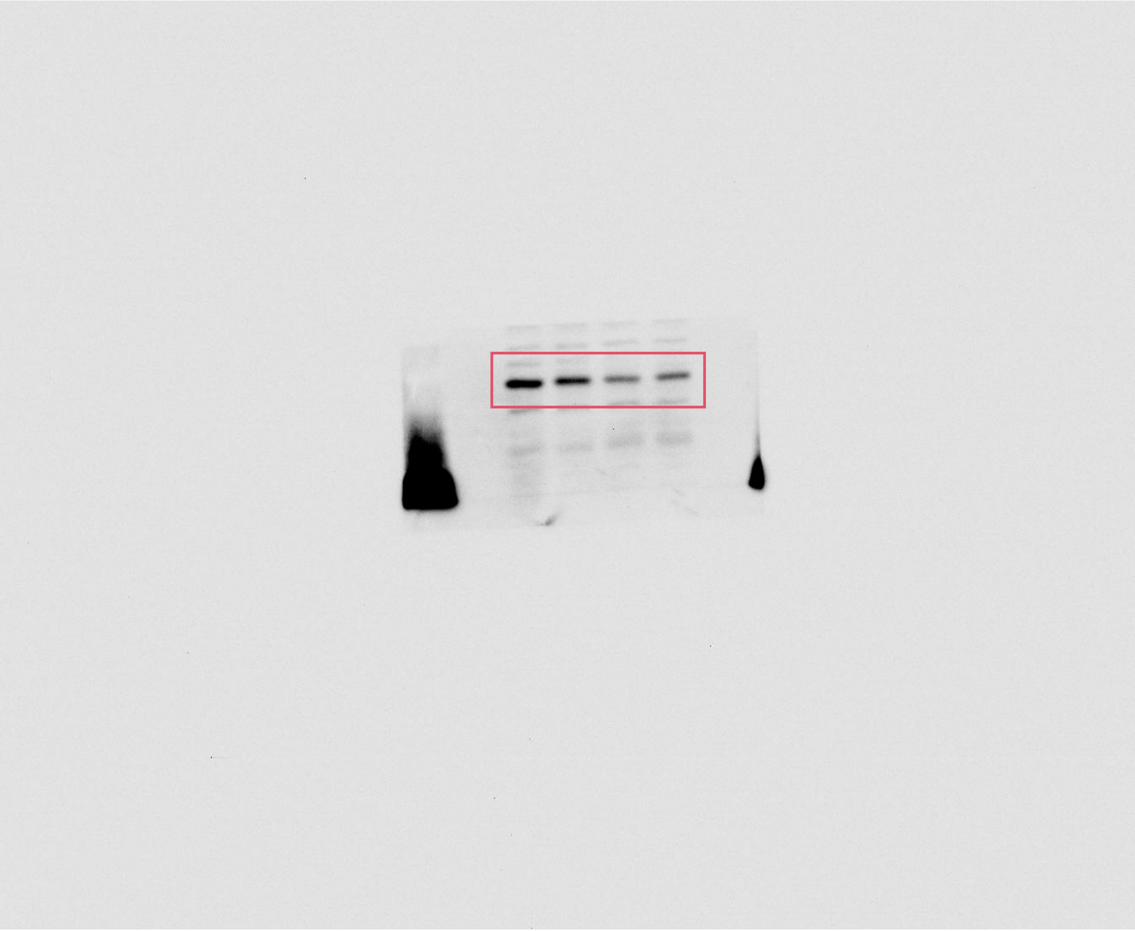

Figure 2D

TUBULIN

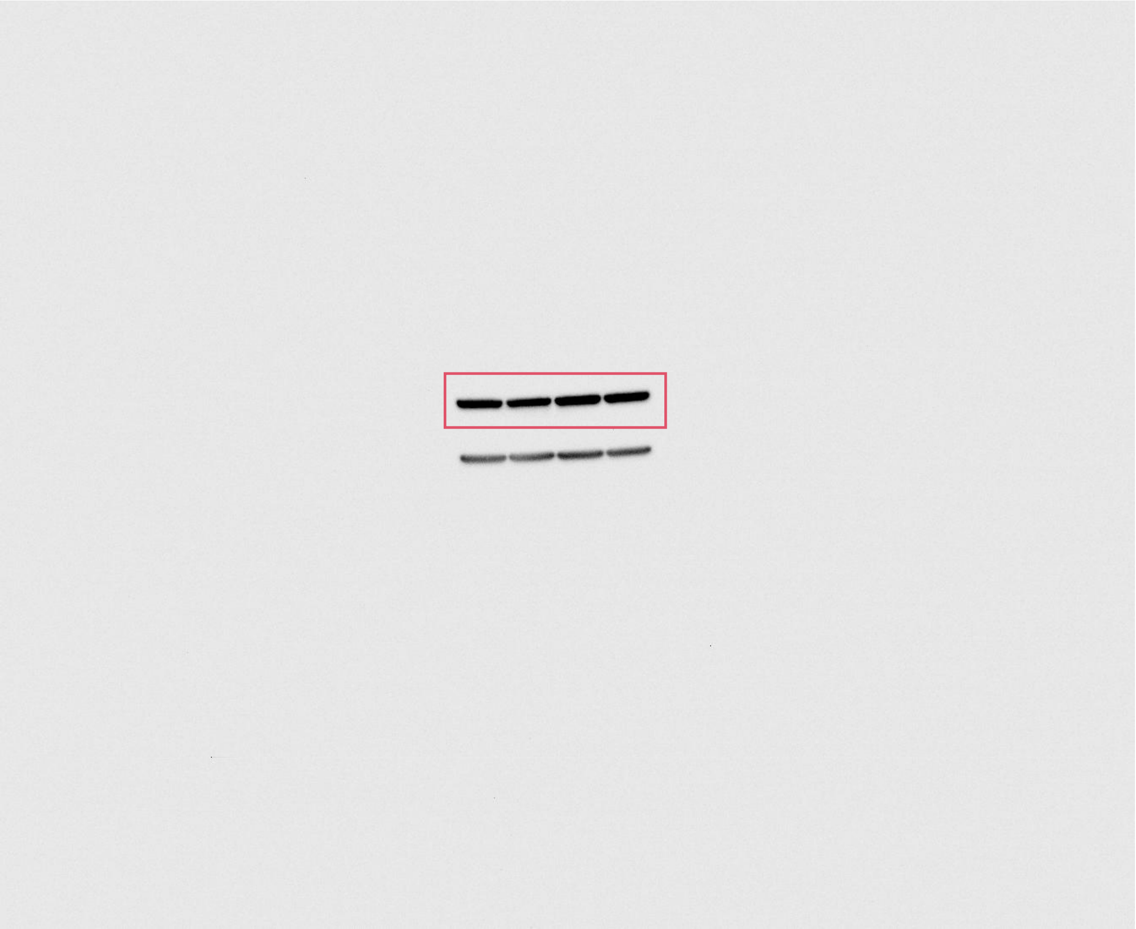

Figure 2E

ATG5

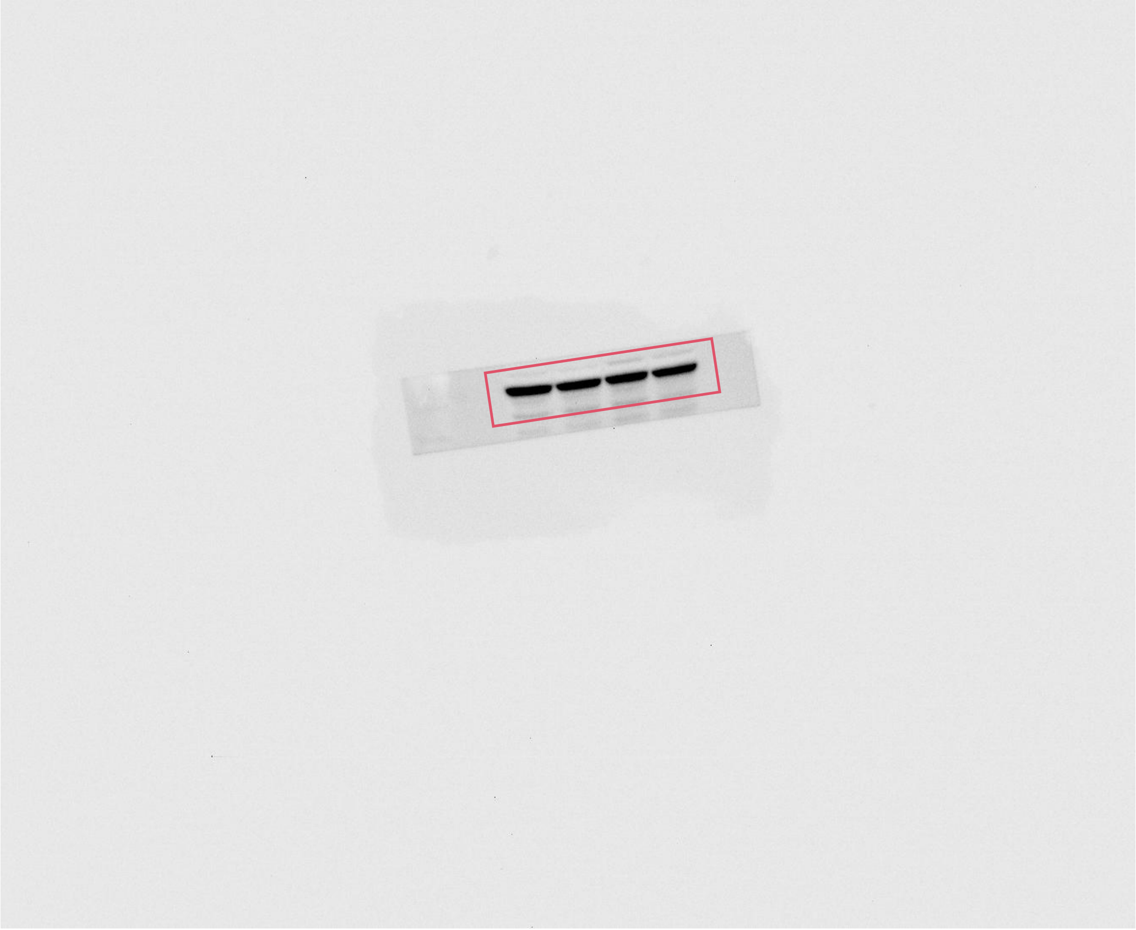

Figure 2E

BECLIN1

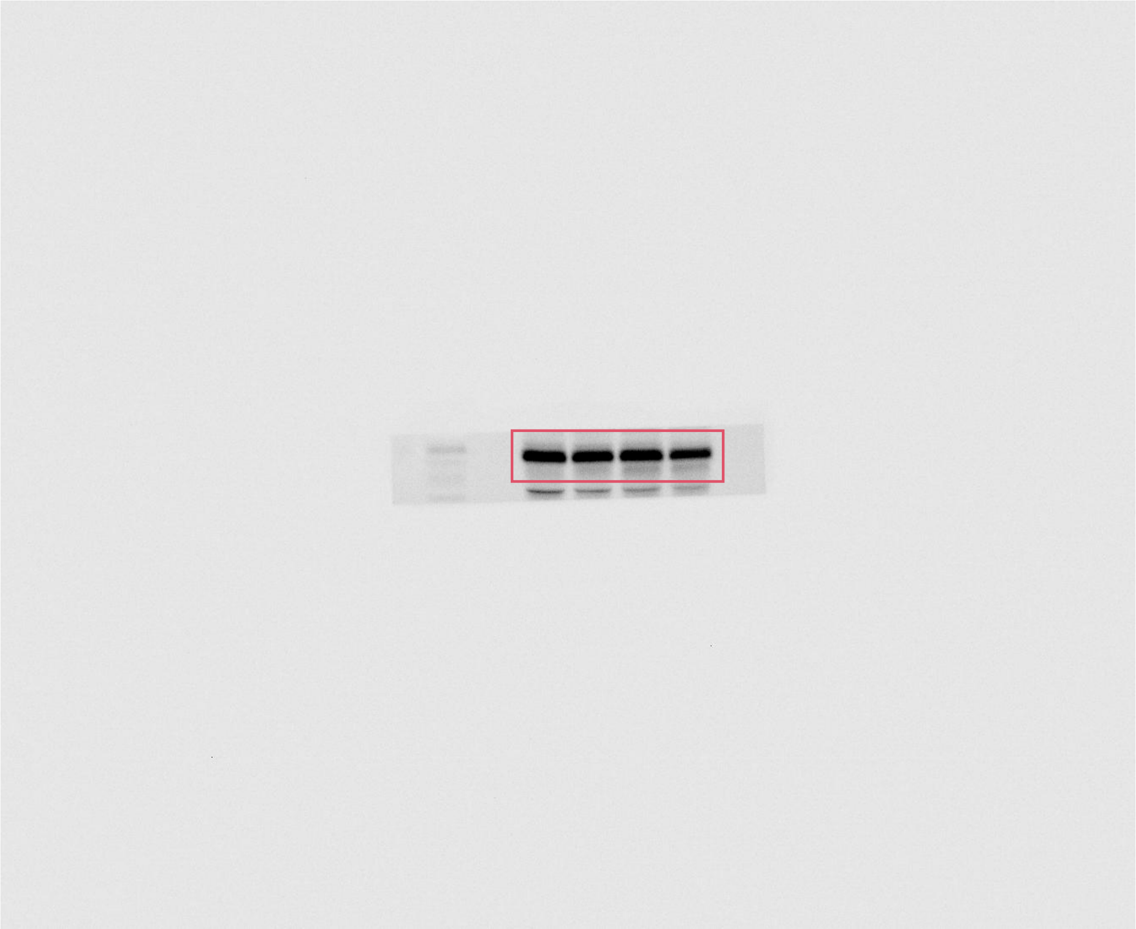

Figure 2E

p62

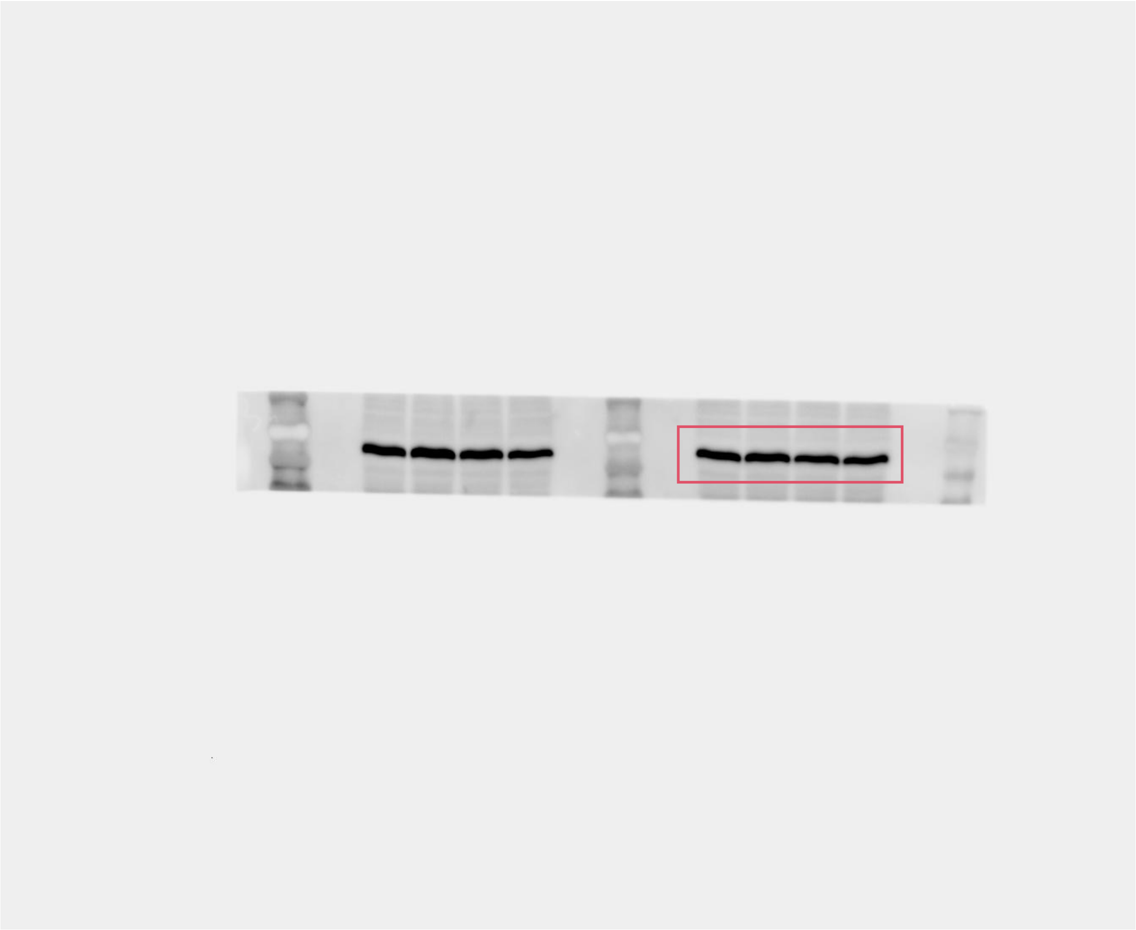

Figure 2E

LC3

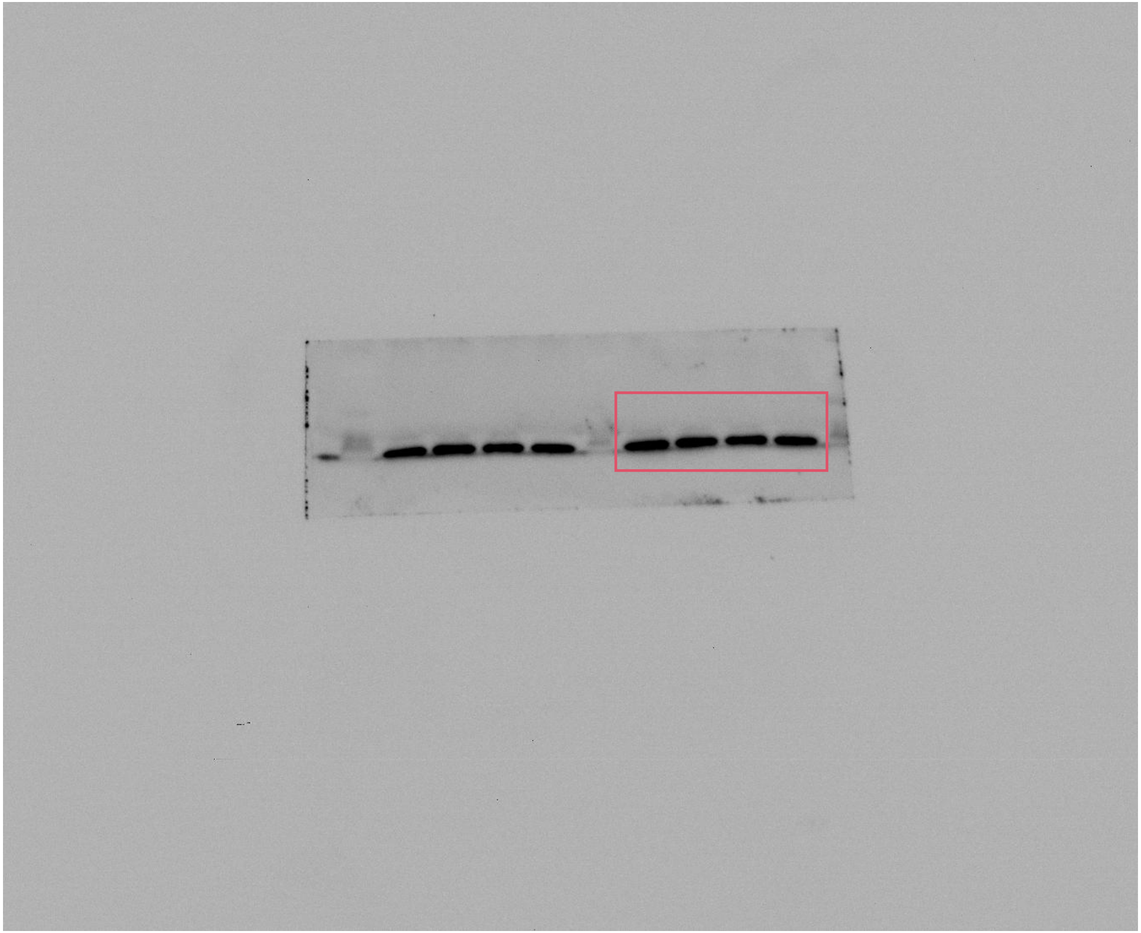

Figure 2E

TUBULIN

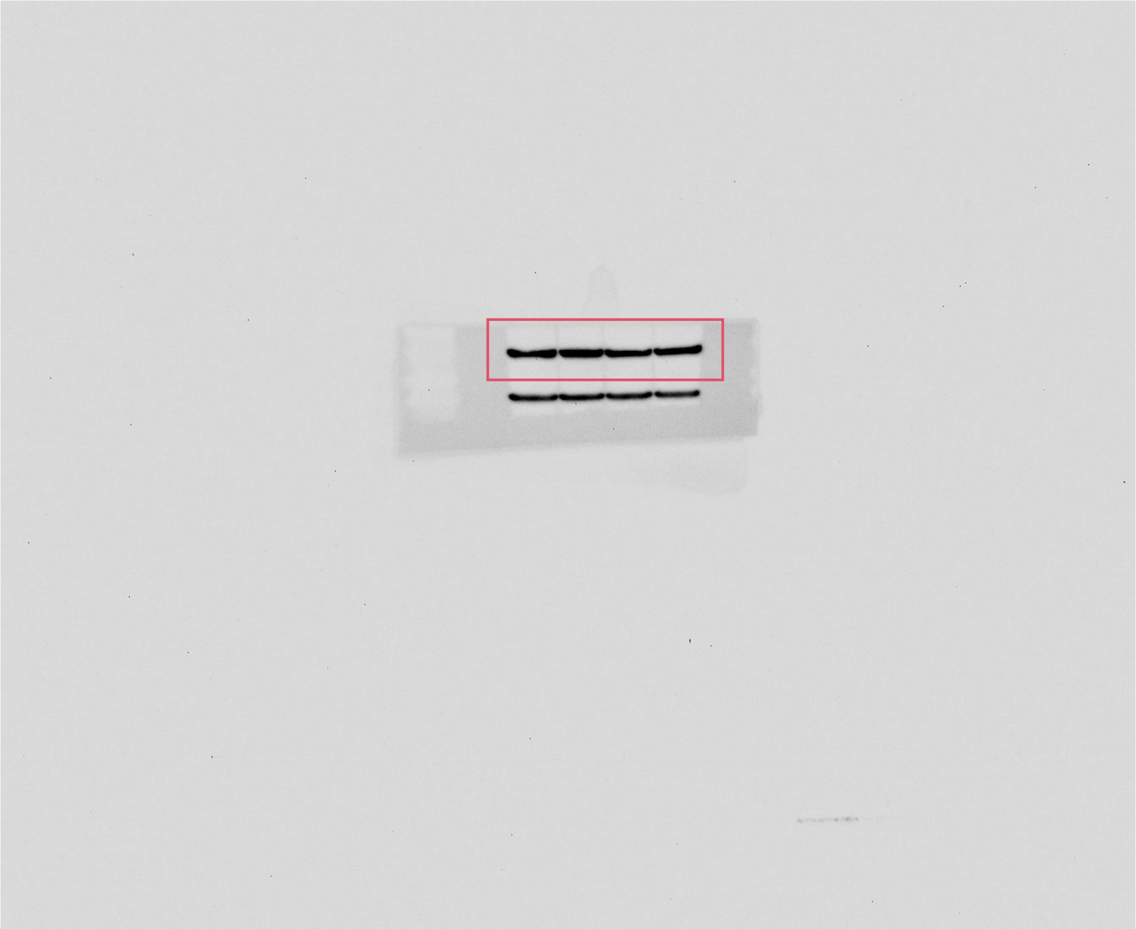

Figure 3A

PTPMT1

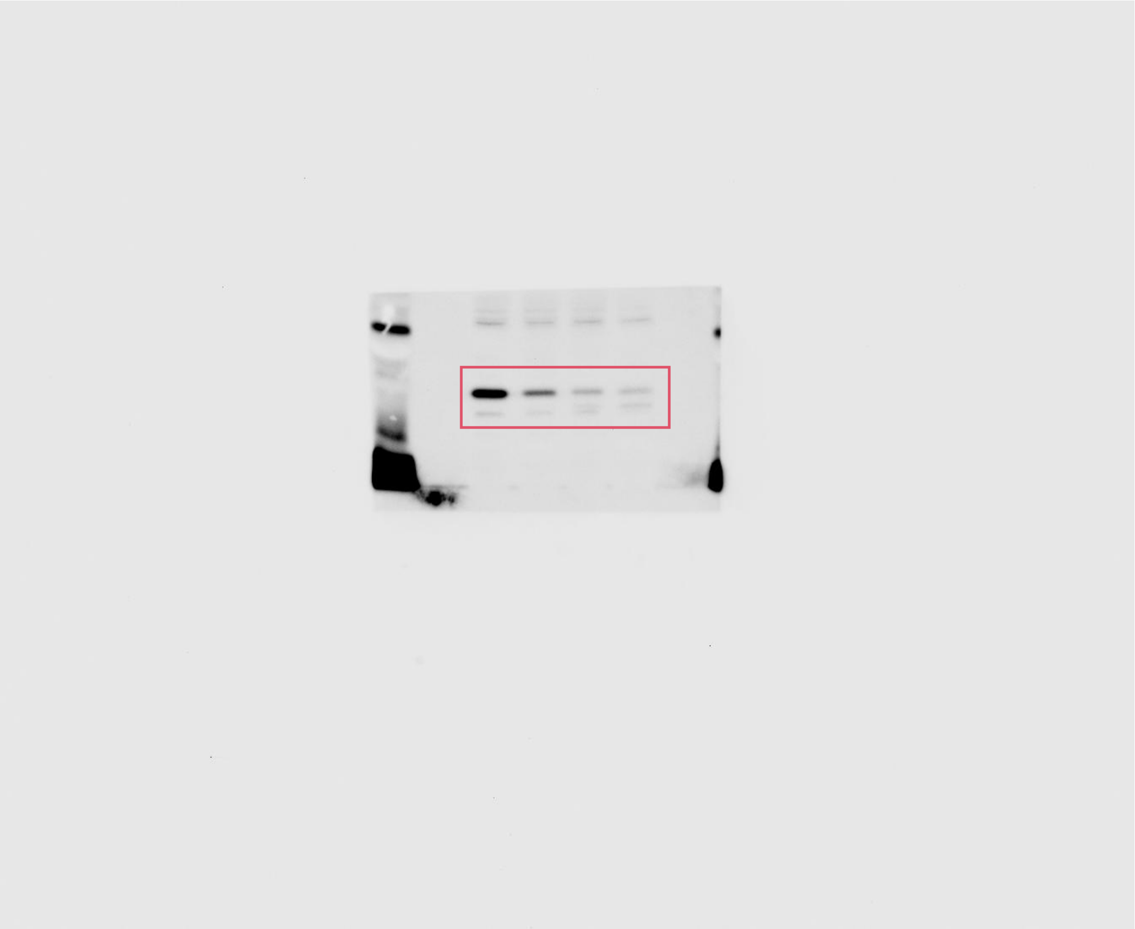

Figure 3A

TUBULIN

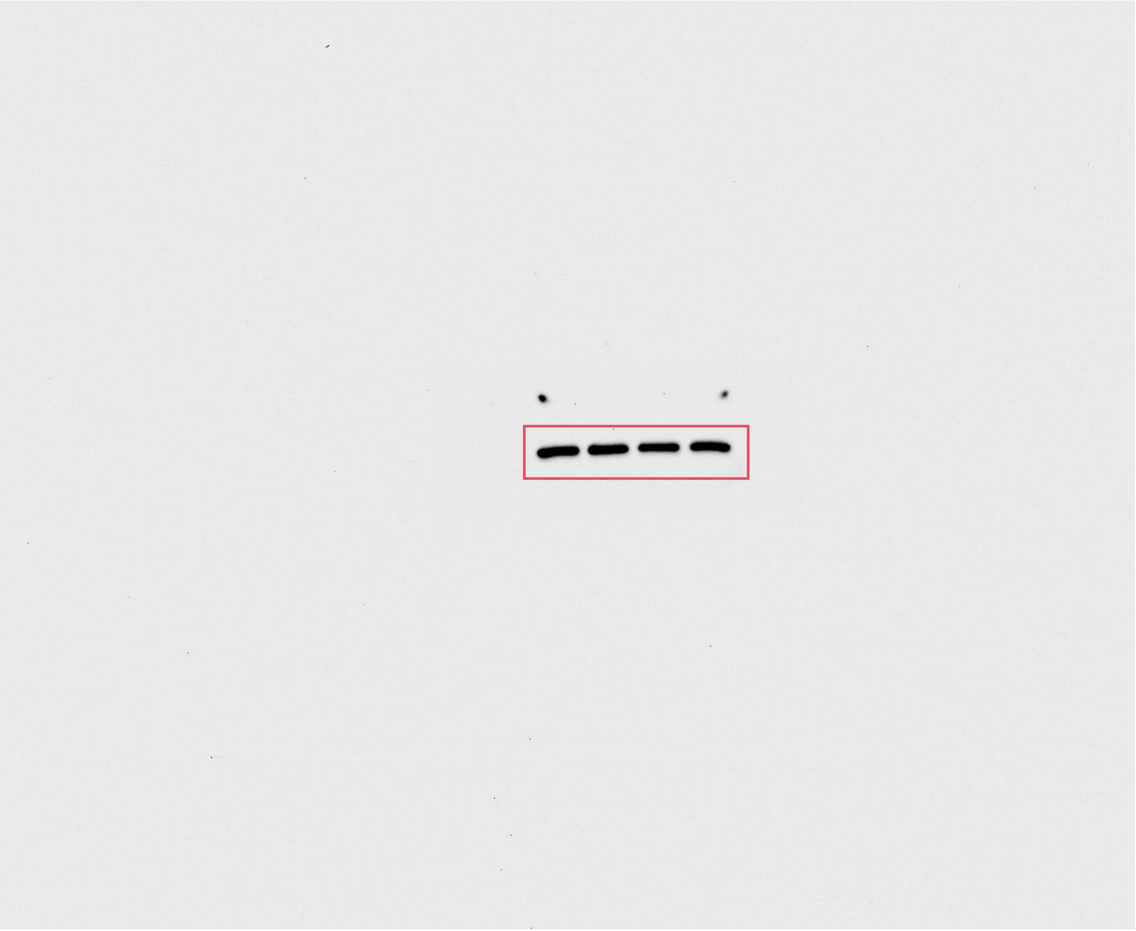

Figure 3B

PTPMT1

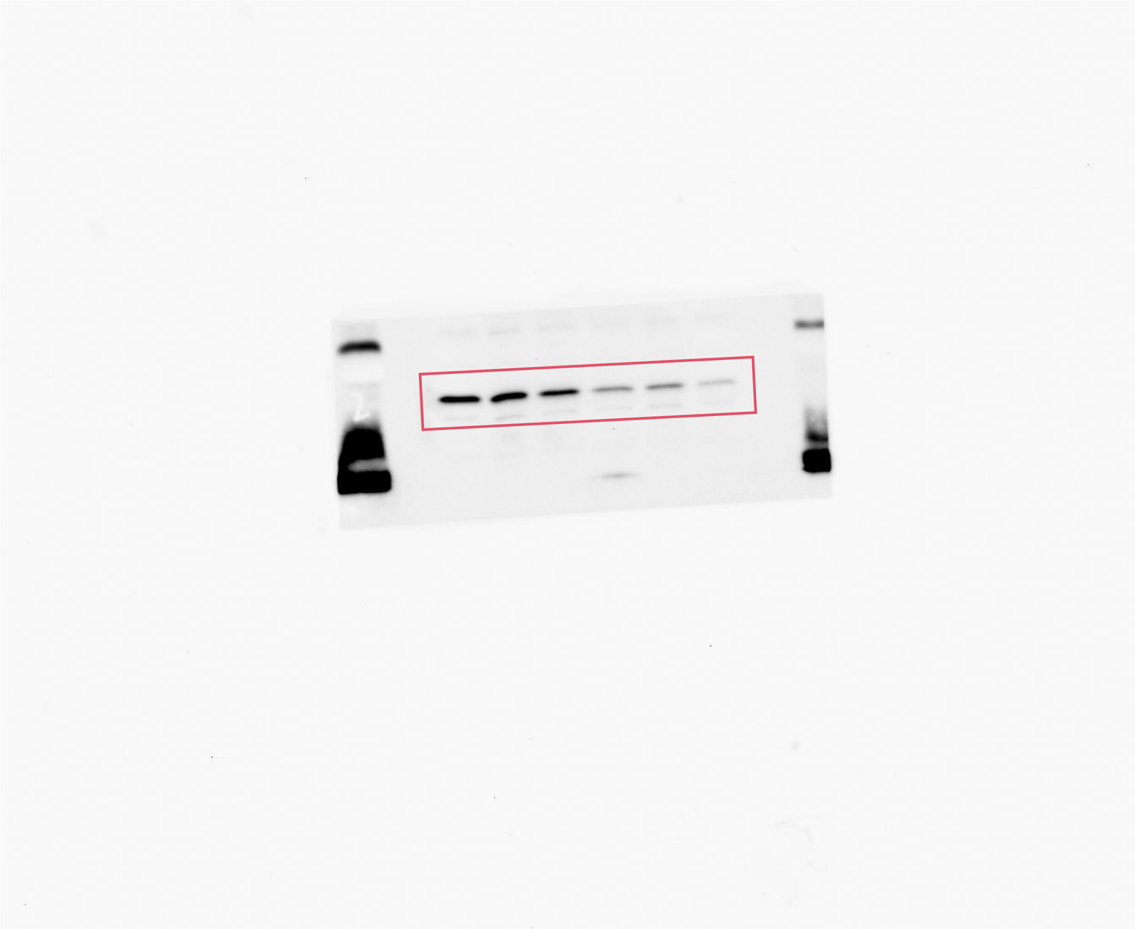

Figure 3B

FTL

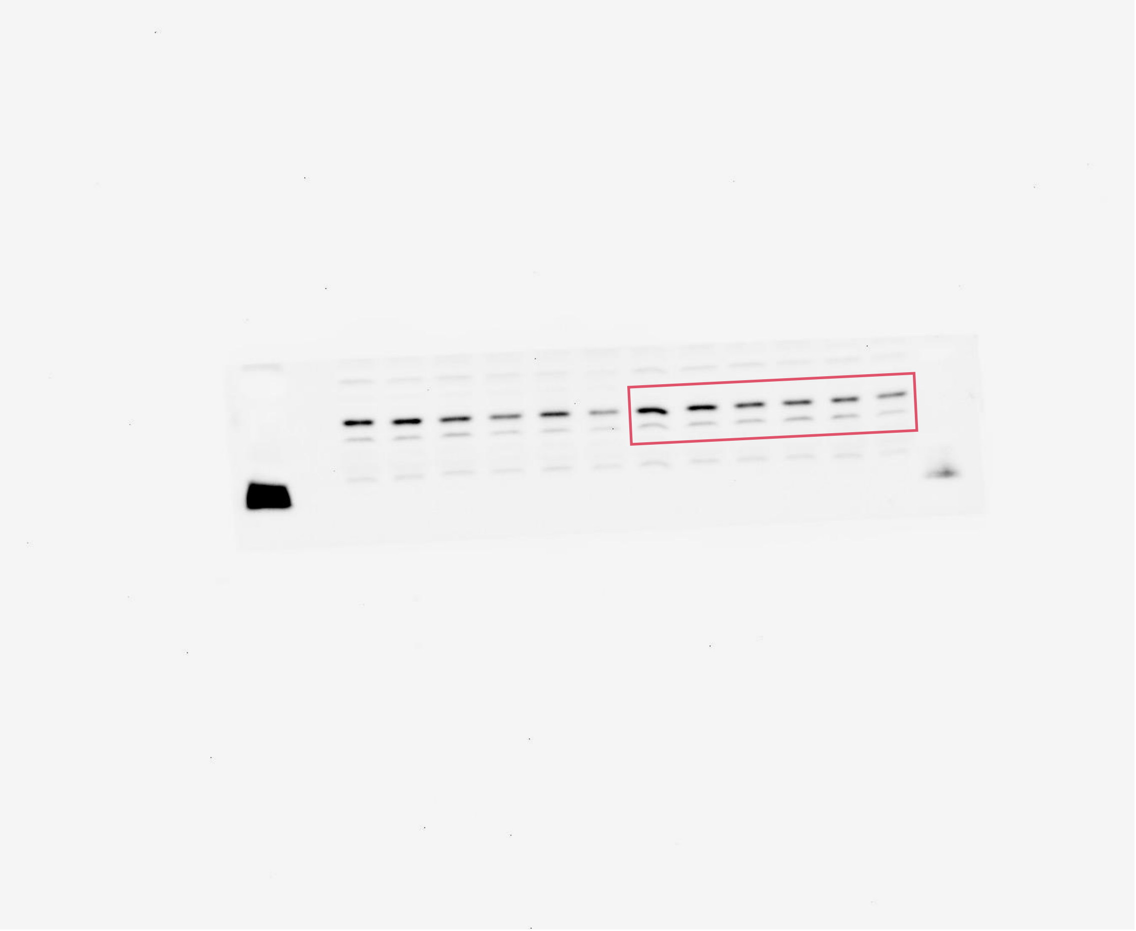

Figure 3B

TUBULIN

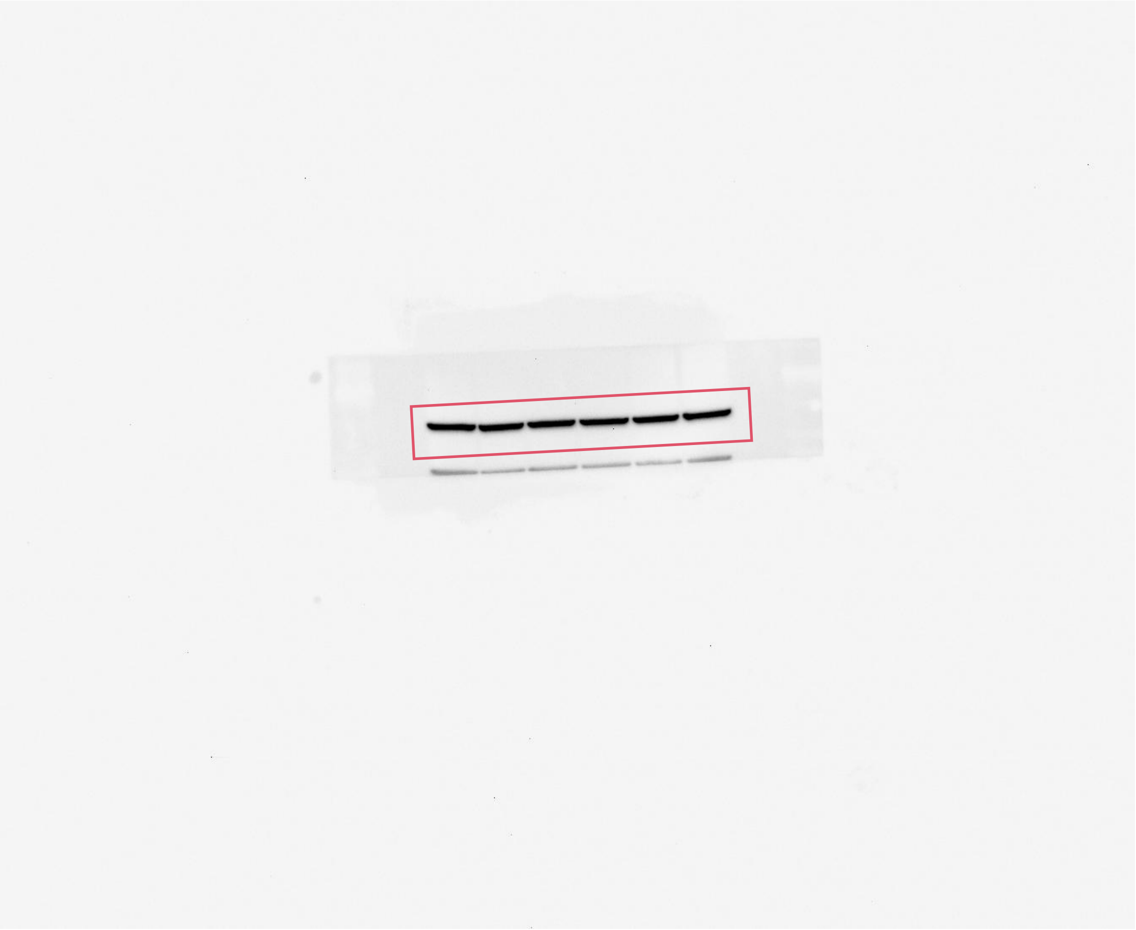

Figure 3C

PTPMT1

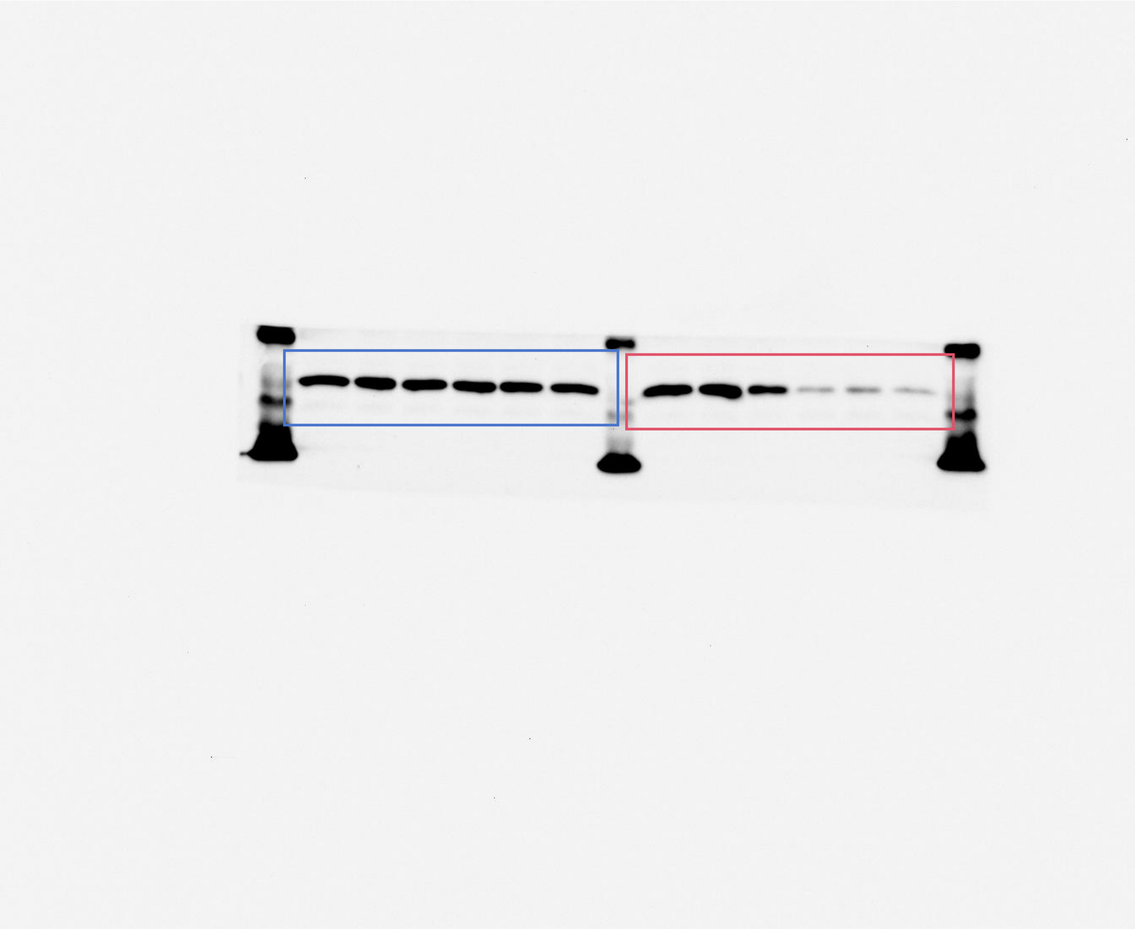

Figure 3C

TUBULIN

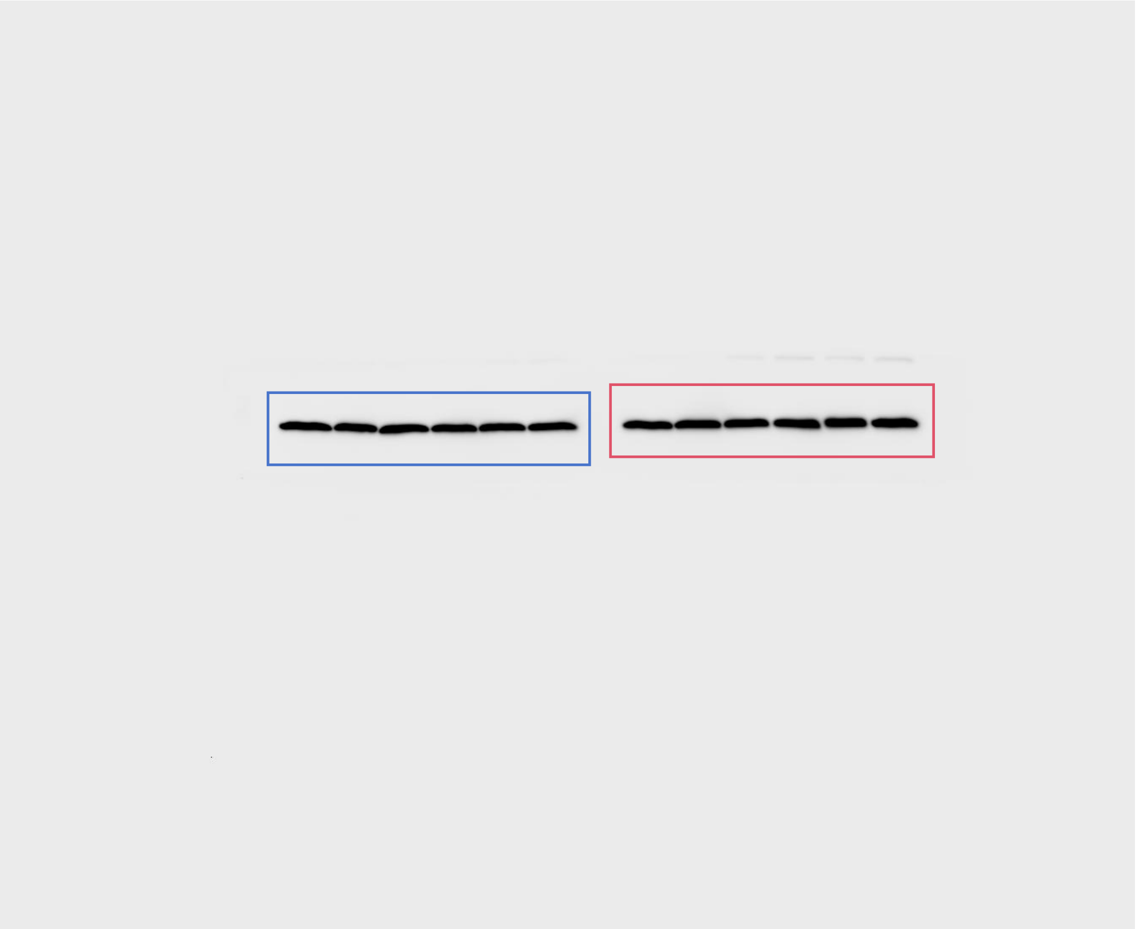

Figure 3E

PTPMT1

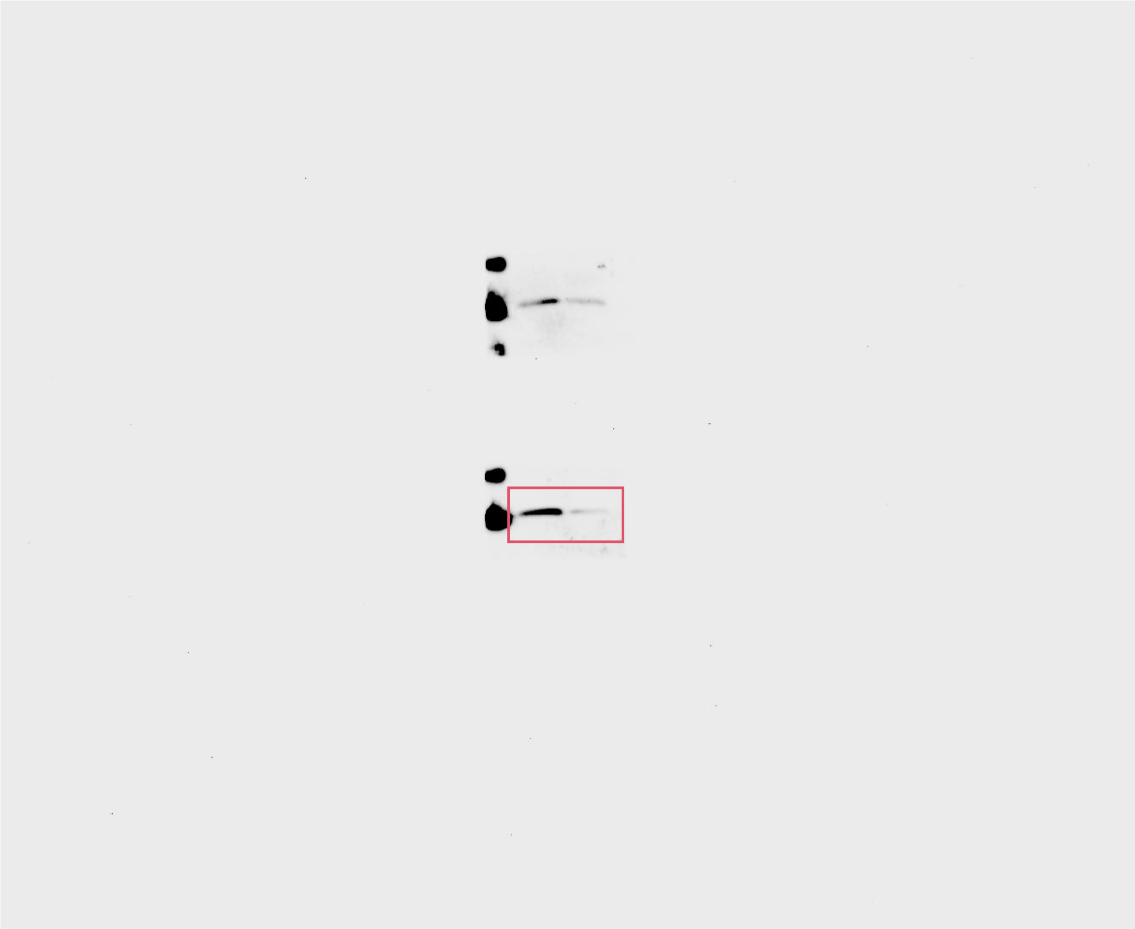

Figure 3E

TUBULIN

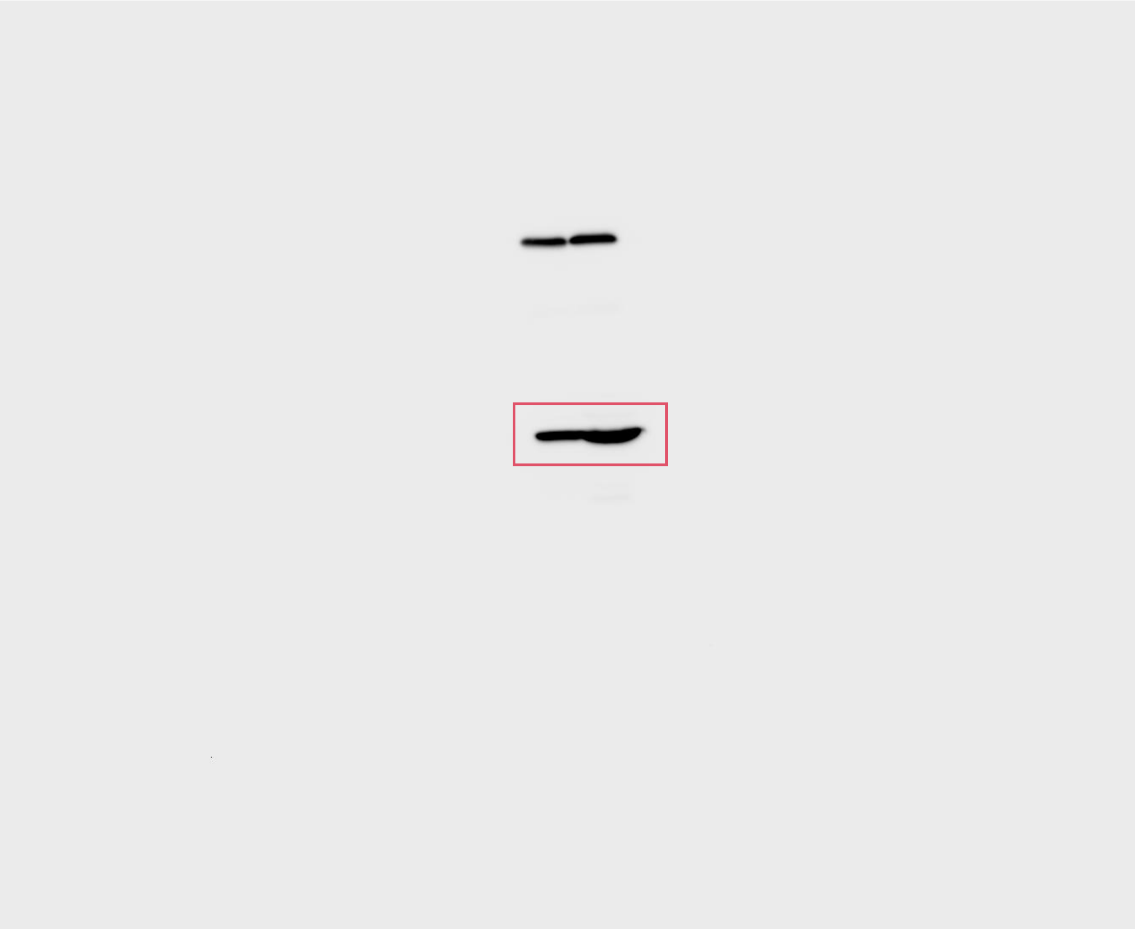

Figure 5A

TOM20

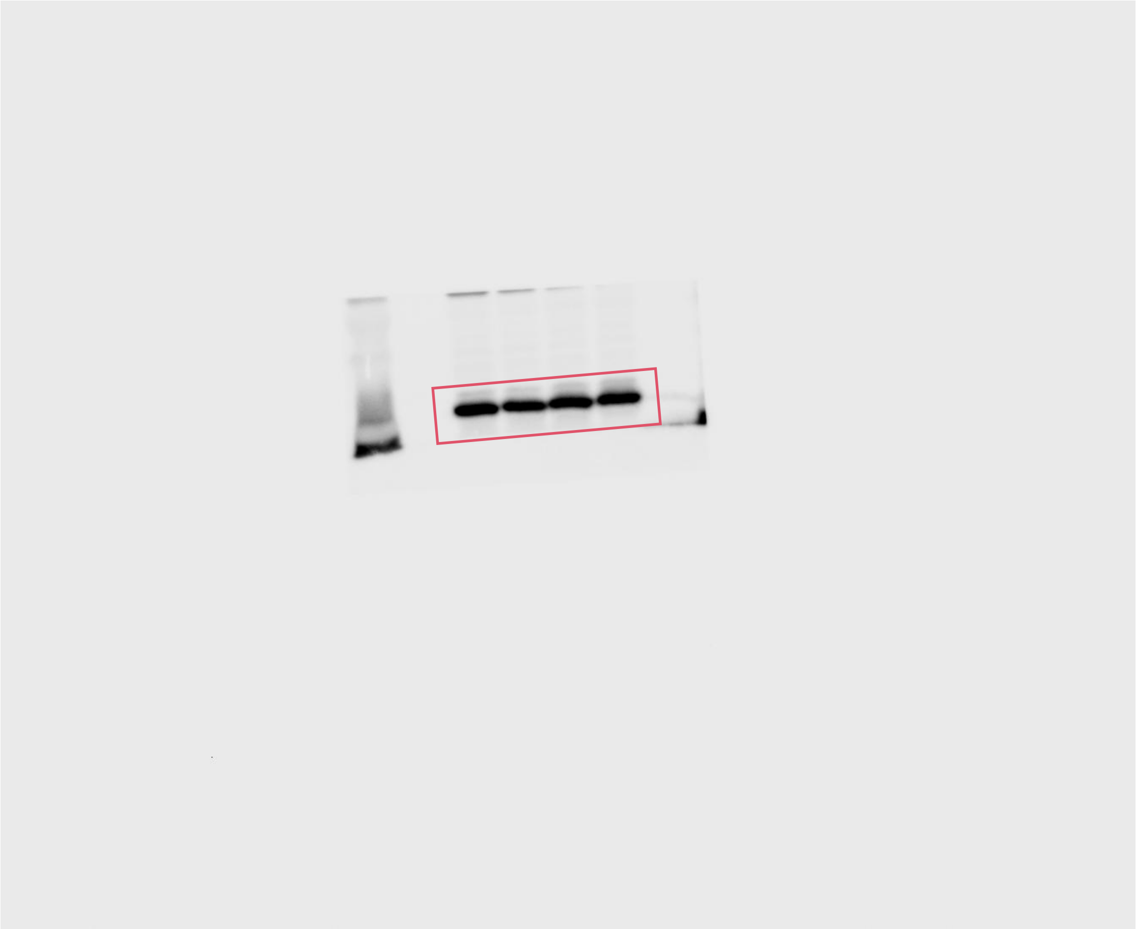

Figure 5A

TUBULIN

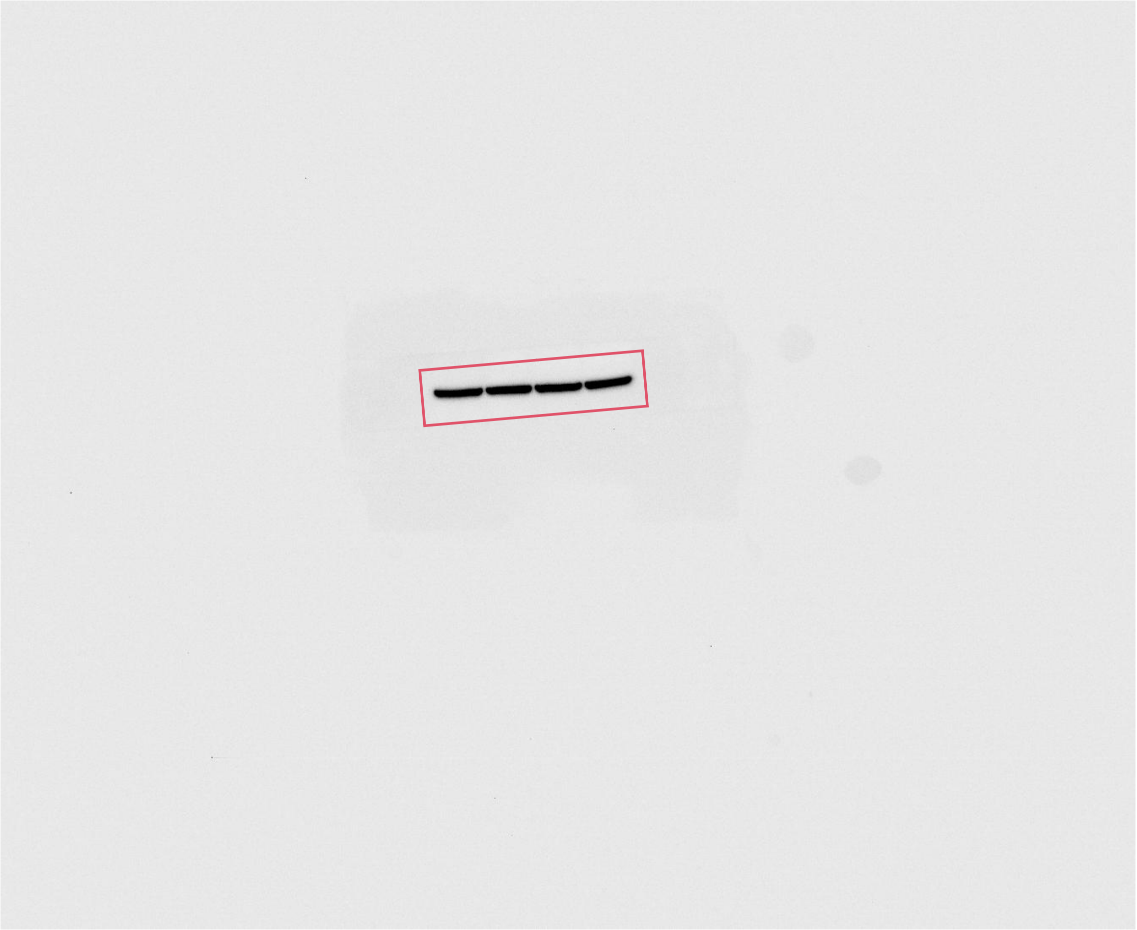

Figure 5E

PINK

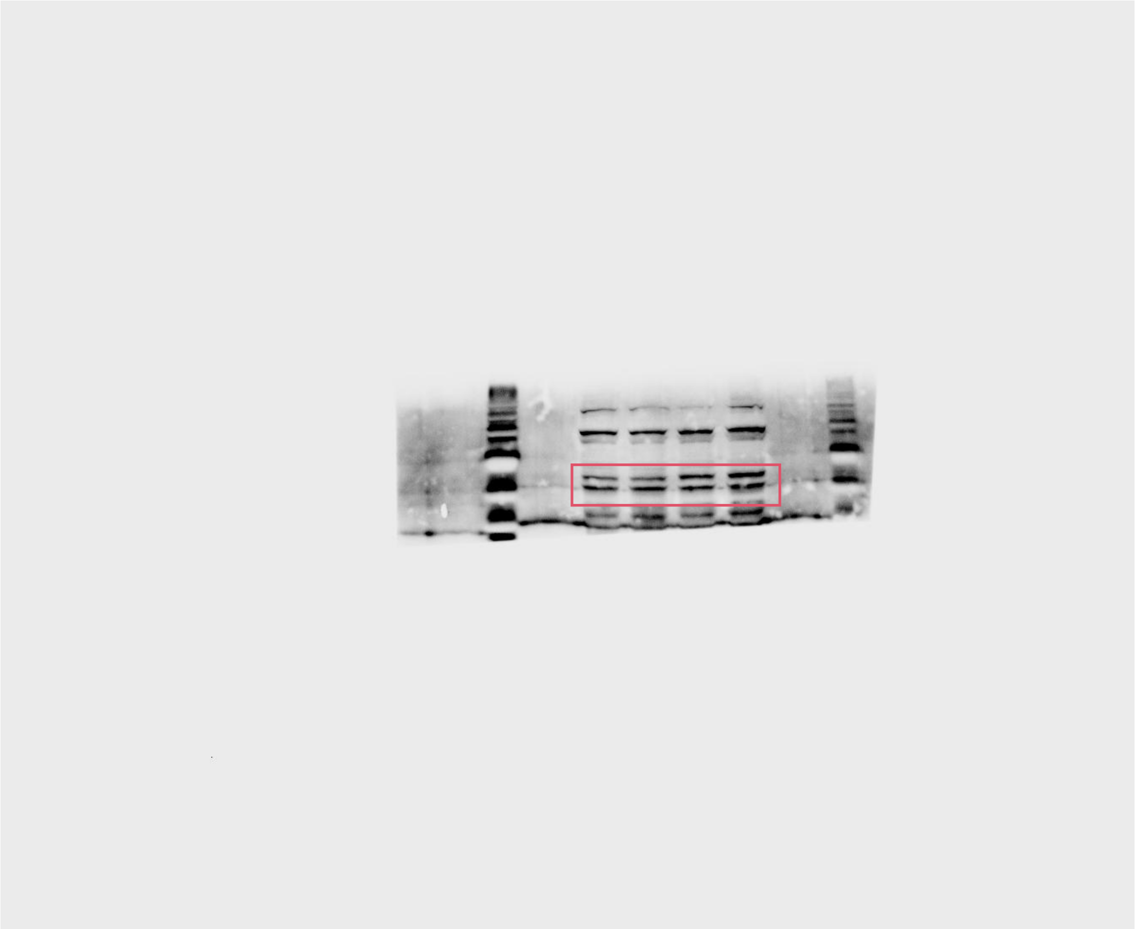

Figure 5E

PGC-1α

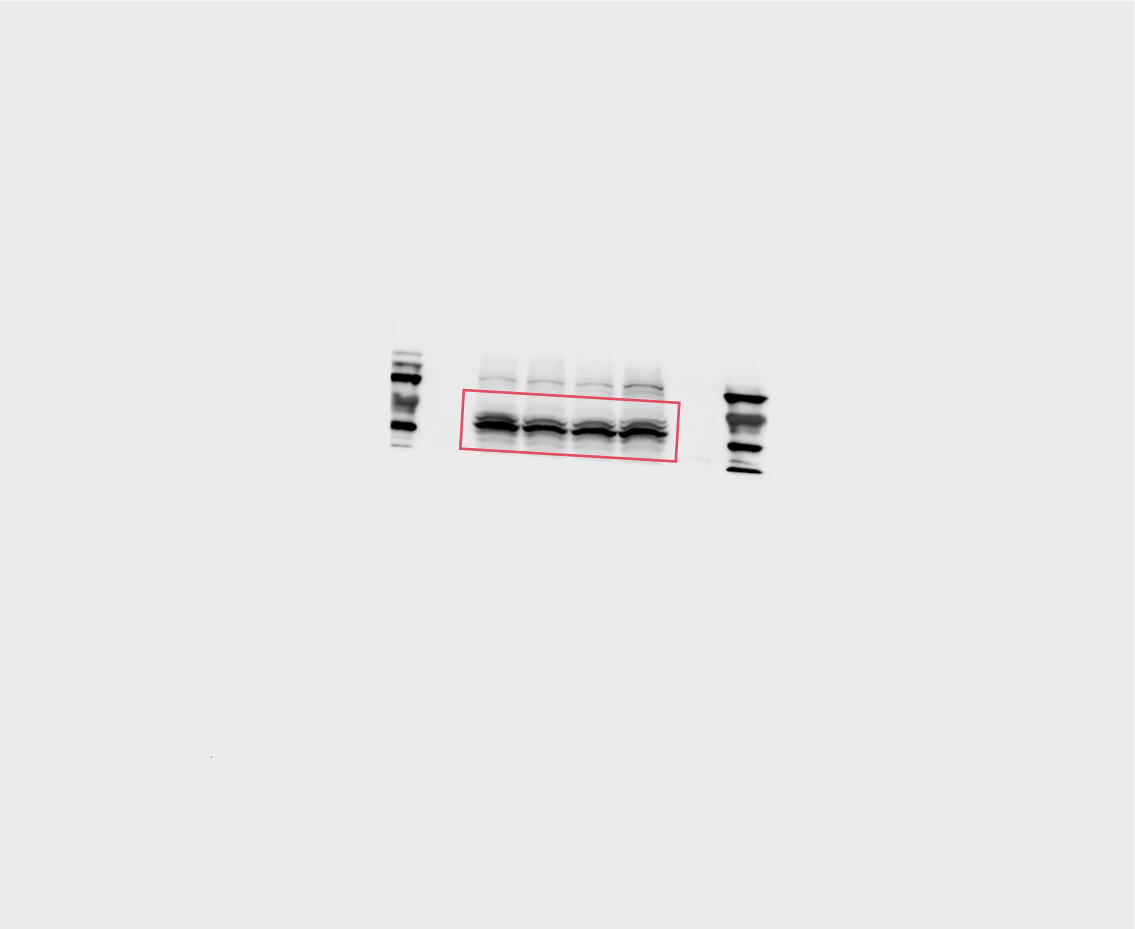

Figure 5E

GAPDH

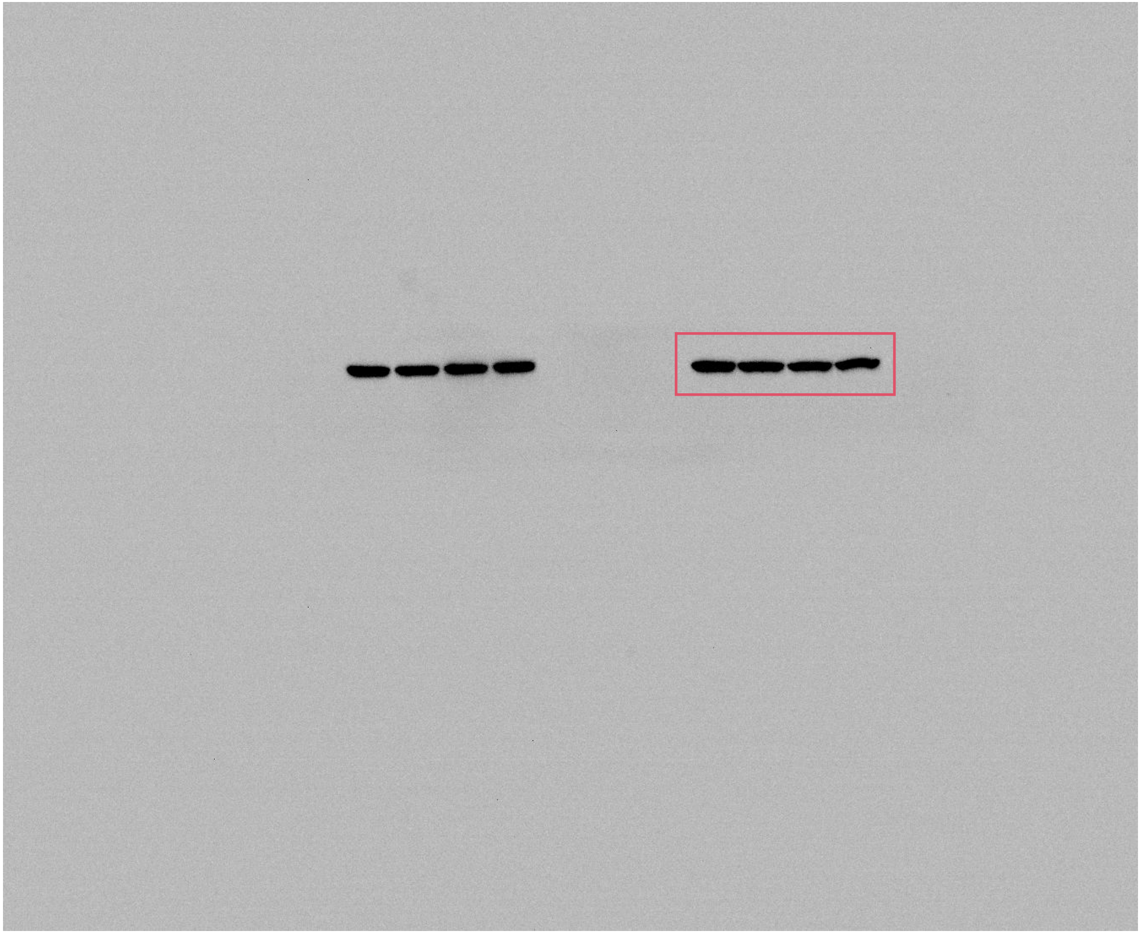

Figure 5F

DRP1

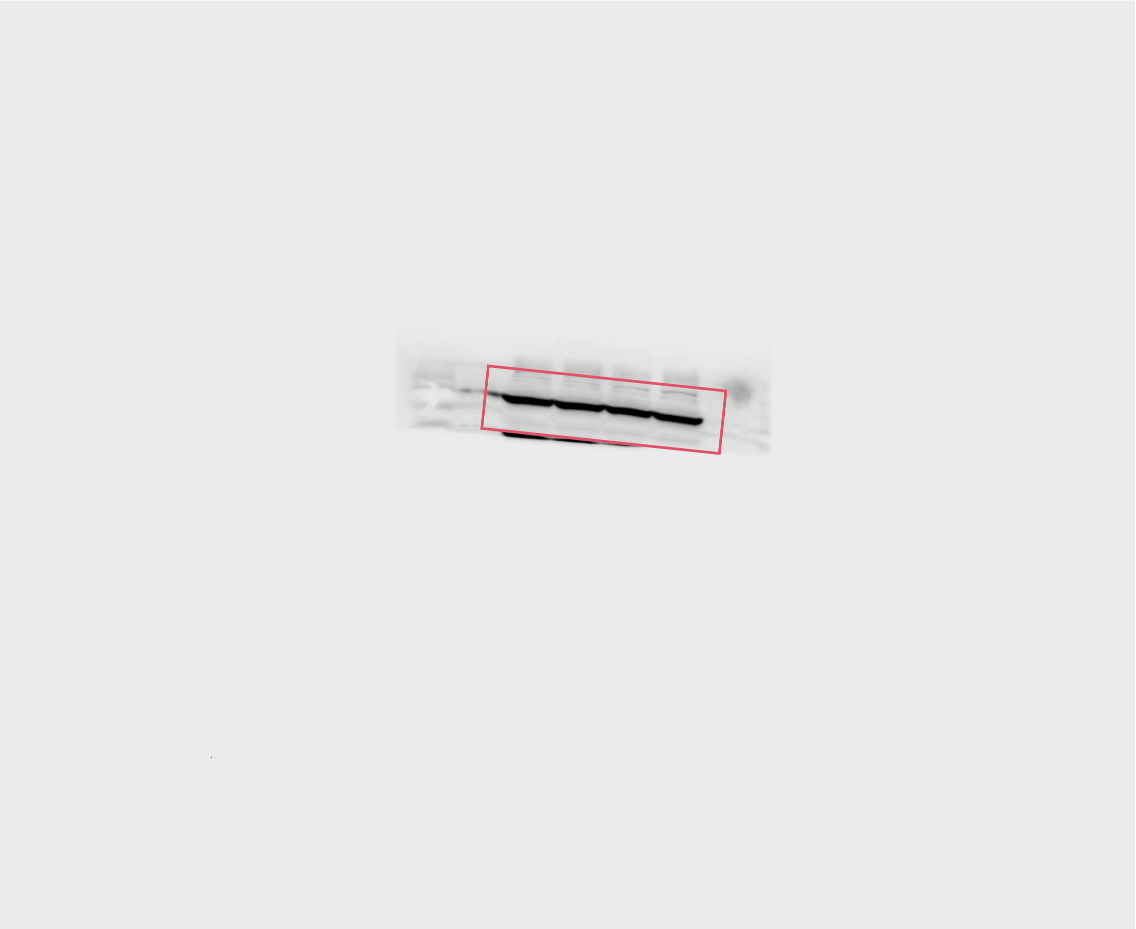

Figure 5F

MFN1

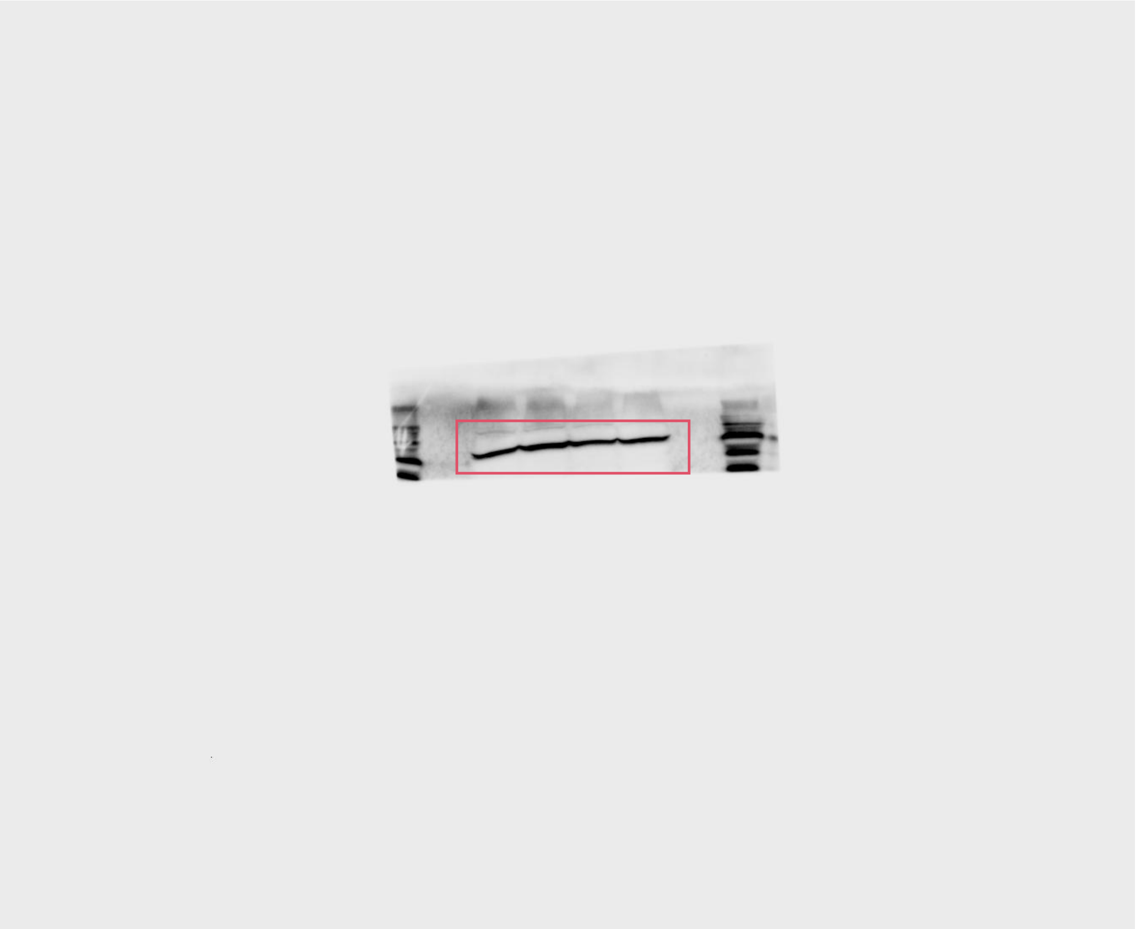

Figure 5F

GAPDH

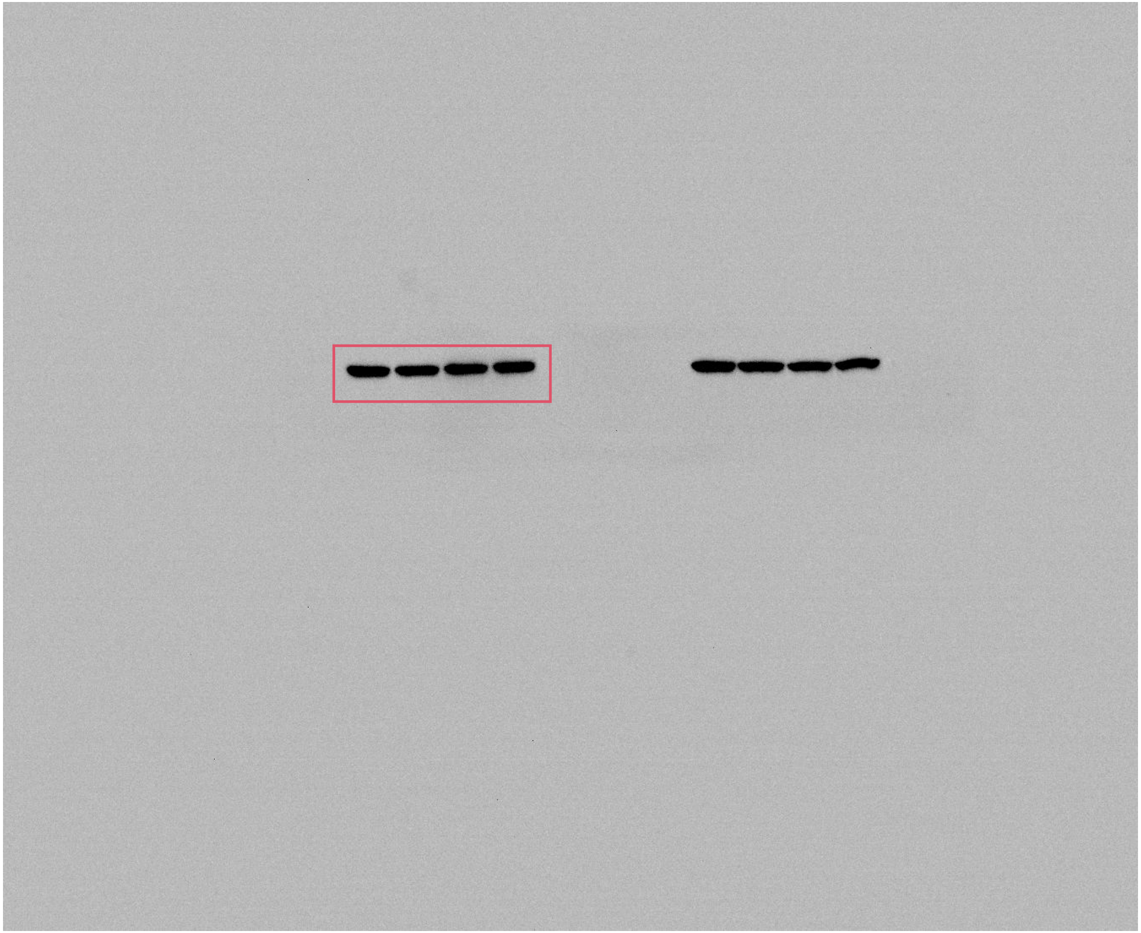

Figure 5G

DRP1

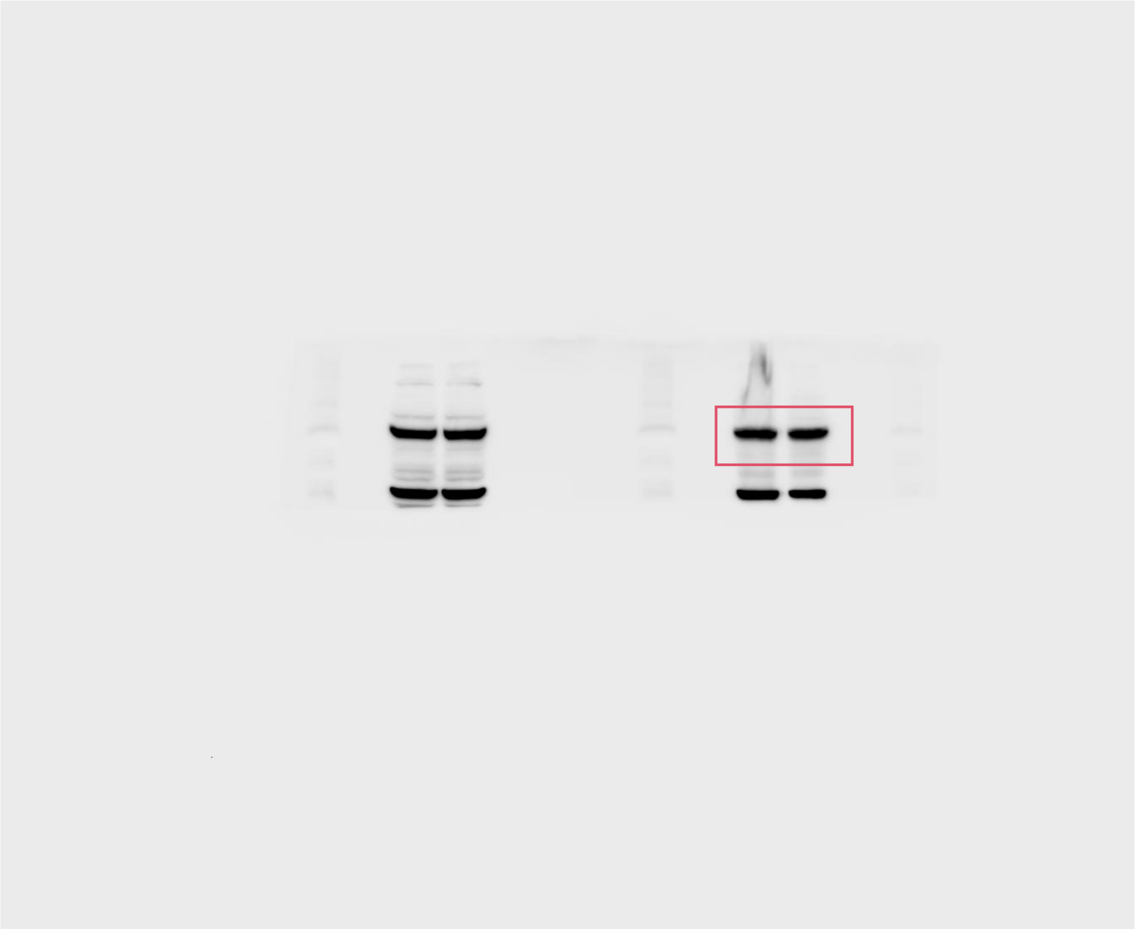

Figure 5G

MFN1

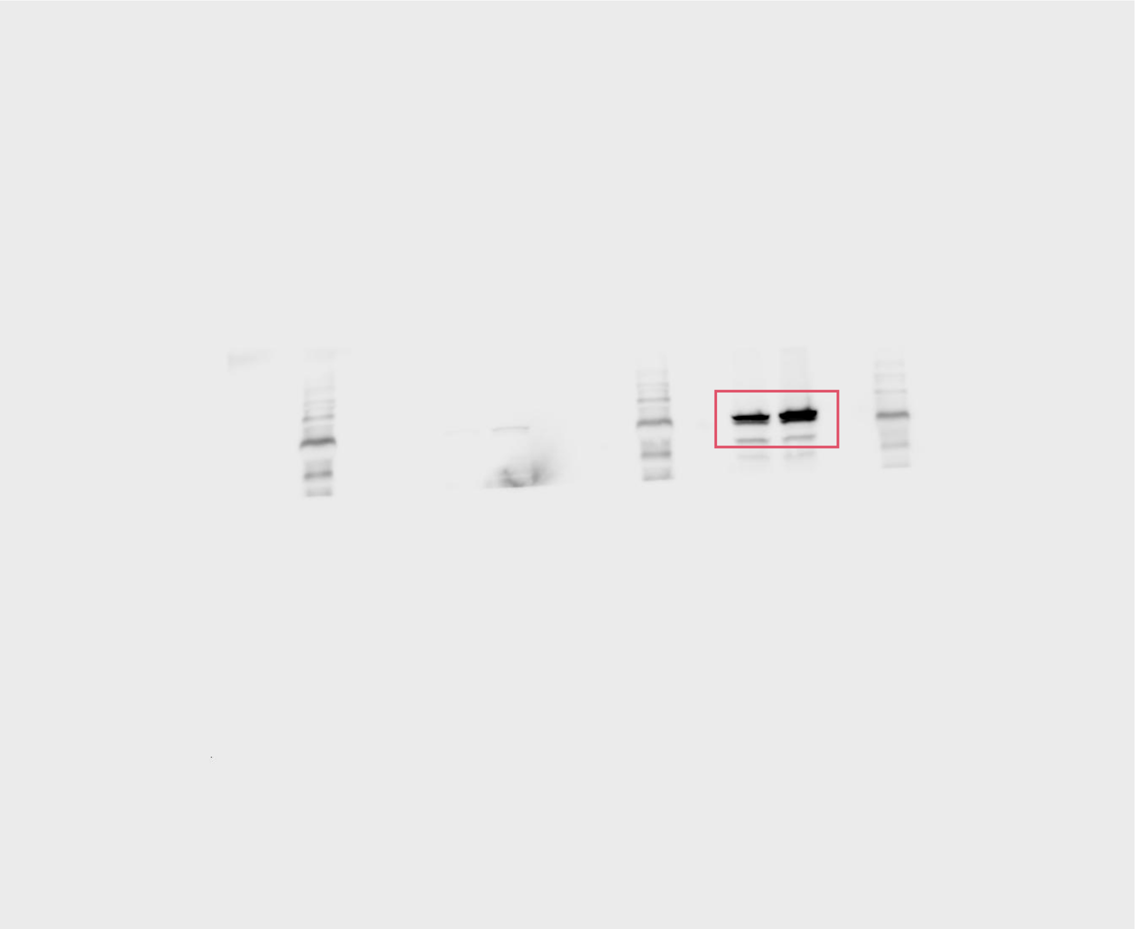

Figure 5G

TOM20

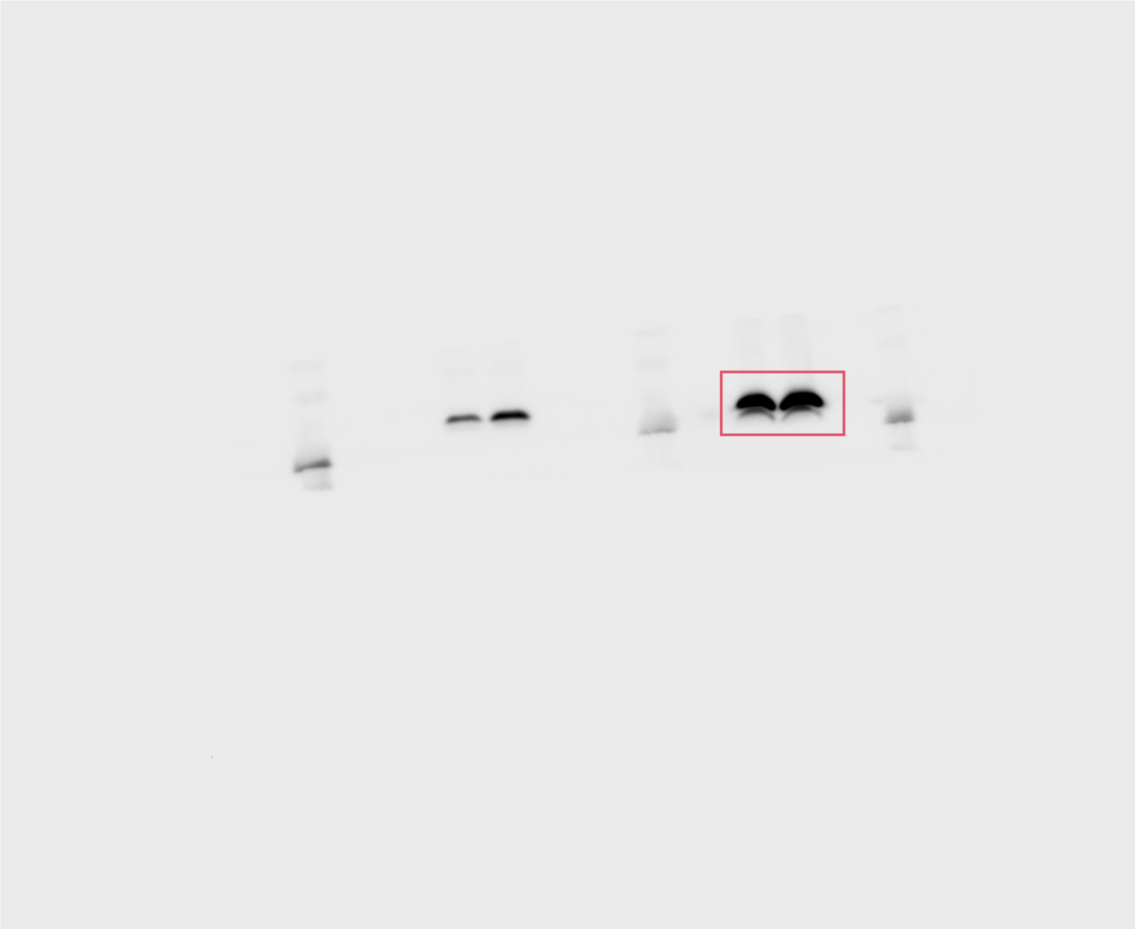

Figure 6E

SDHA

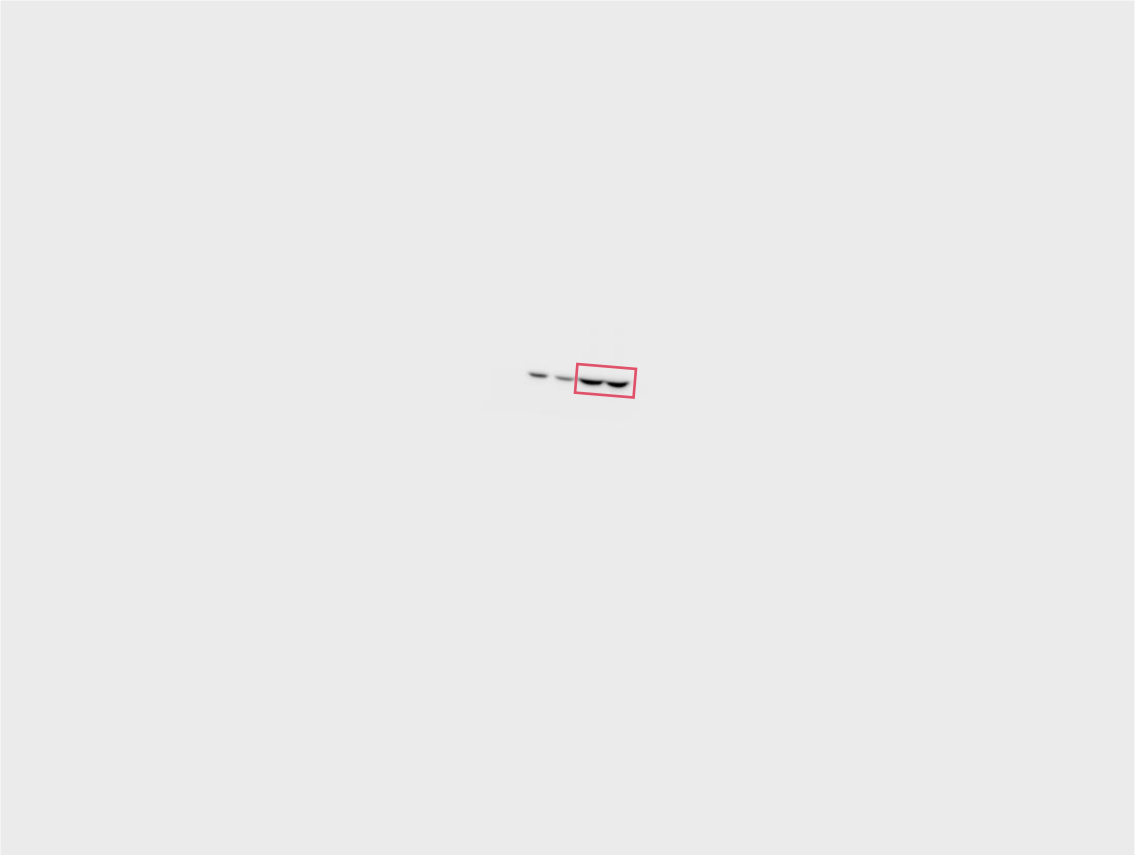

Figure 6E

TUBULIN

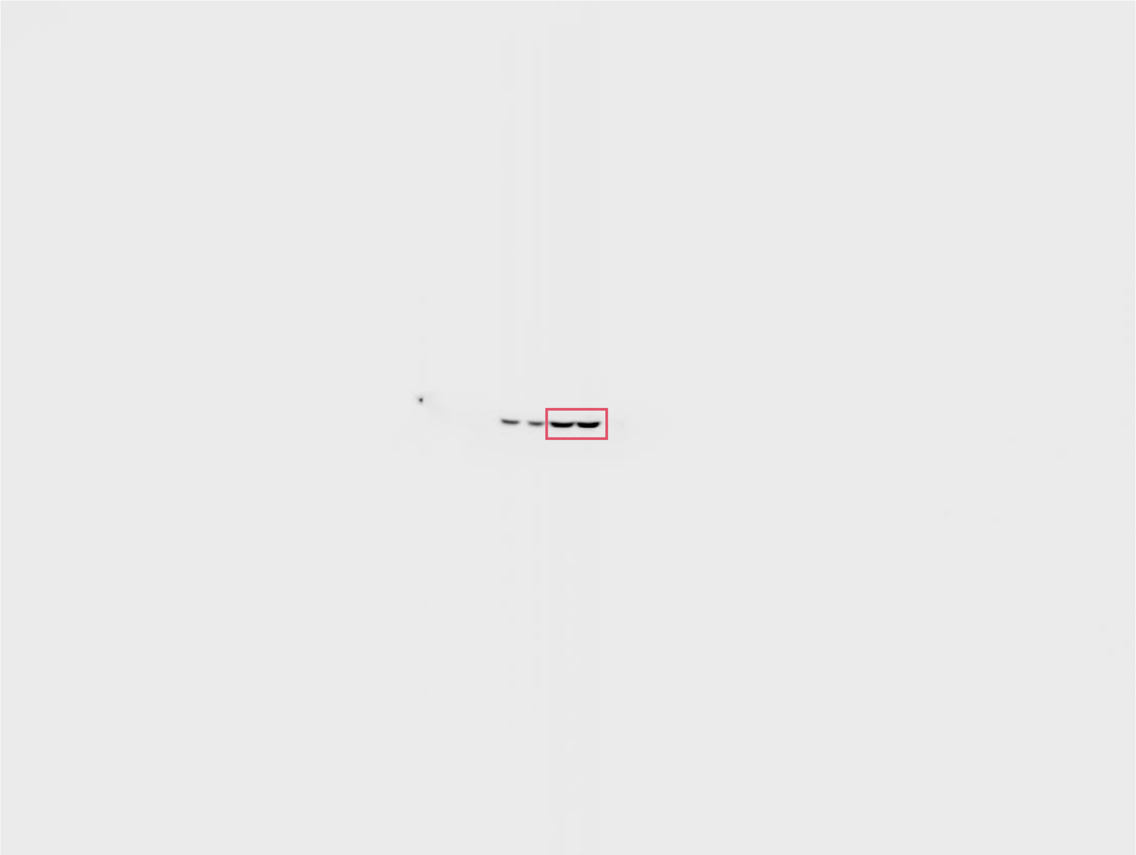

Figure S2A

PTPMT1

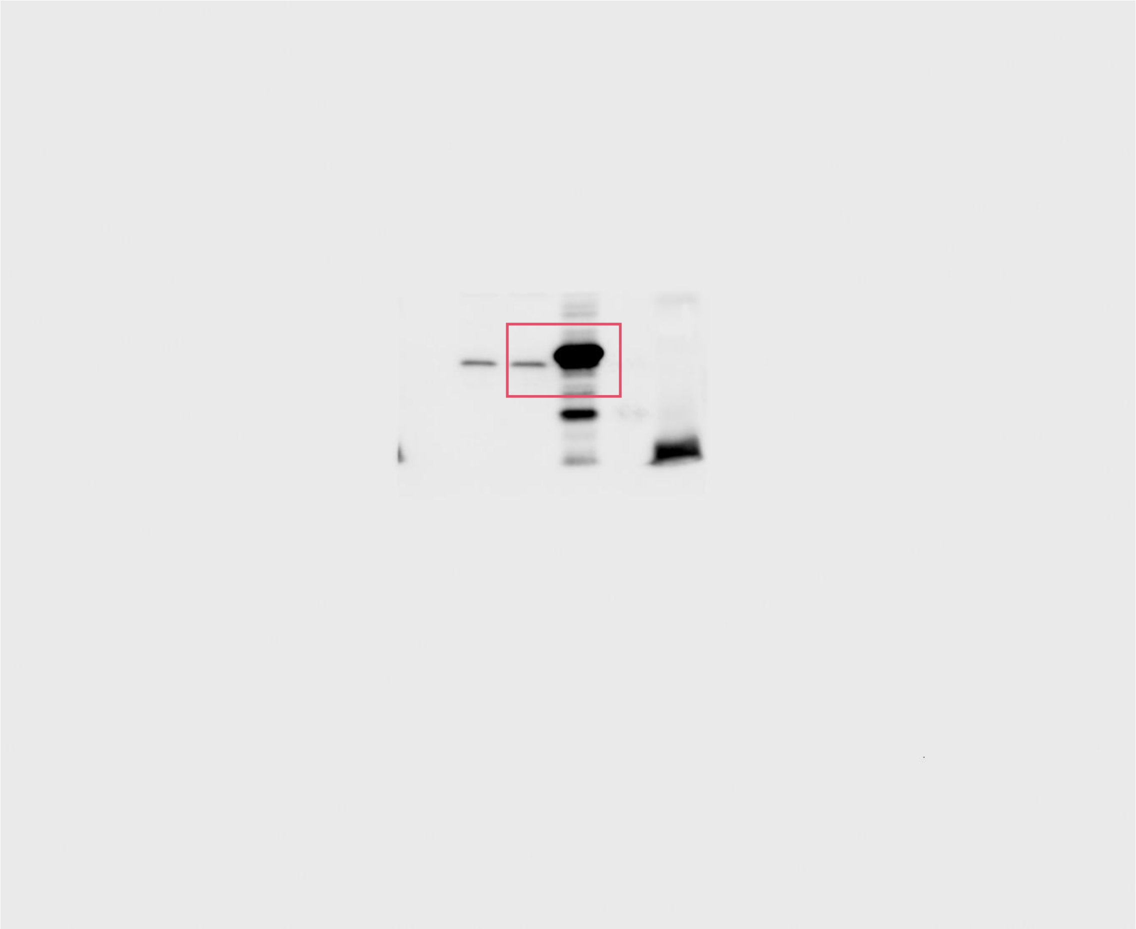

Figure S2A

NRF2

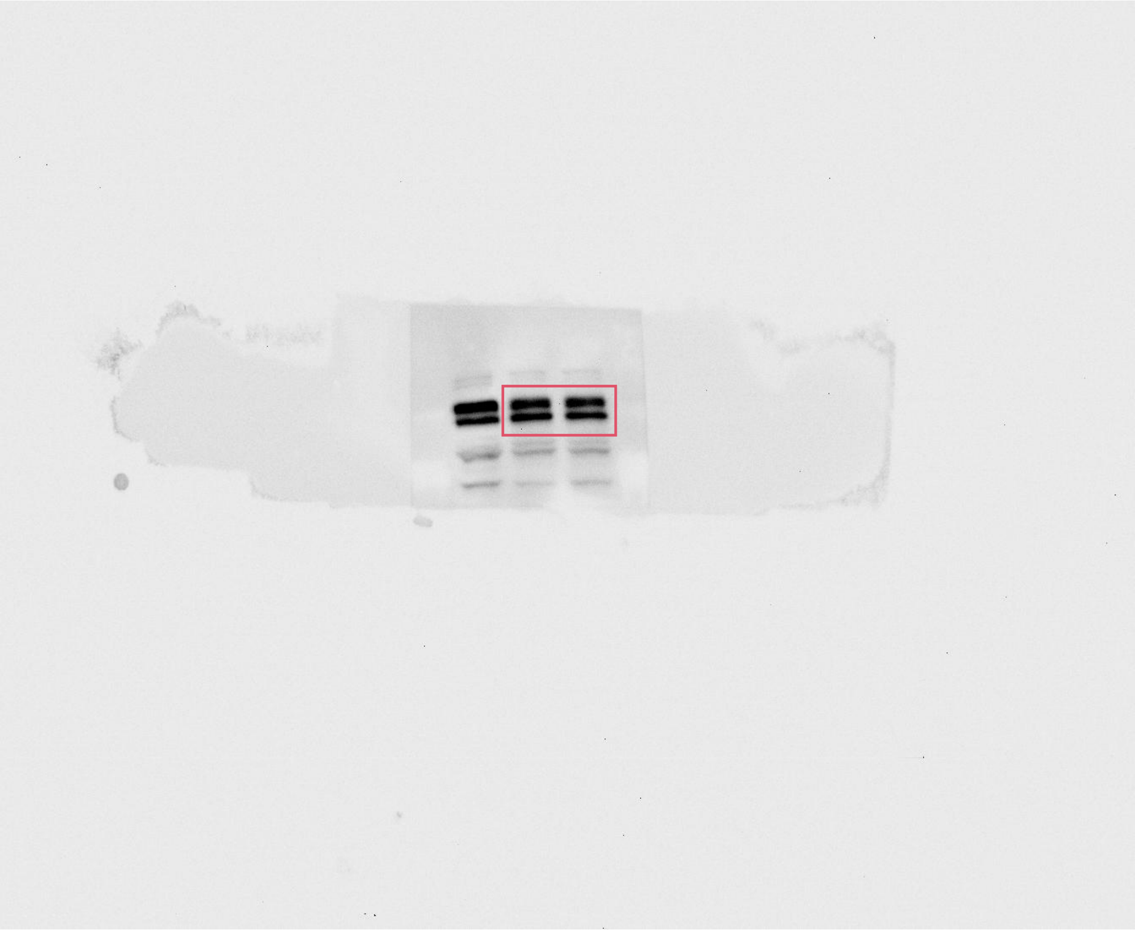

Figure S2A

GPX4

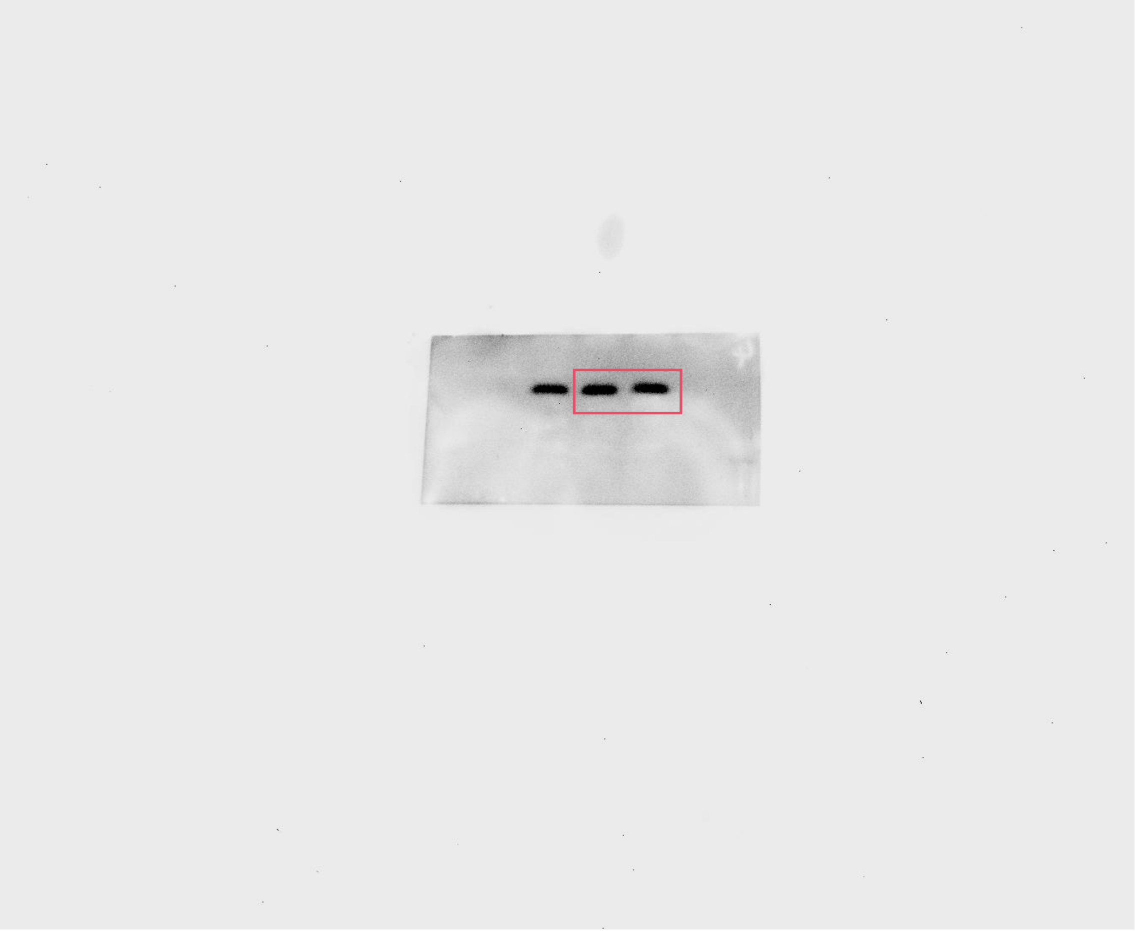

Figure S2A

SLC7A11

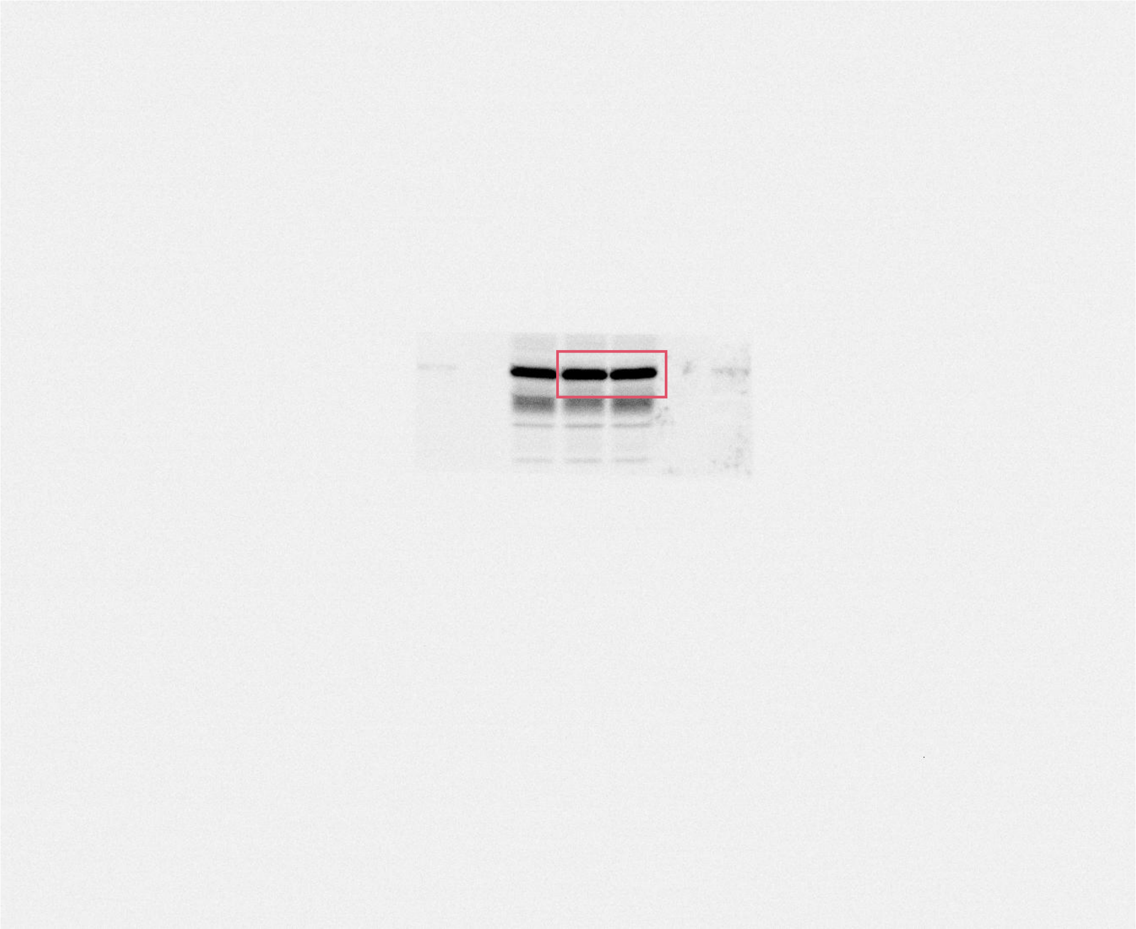

Figure S2A

DHODH

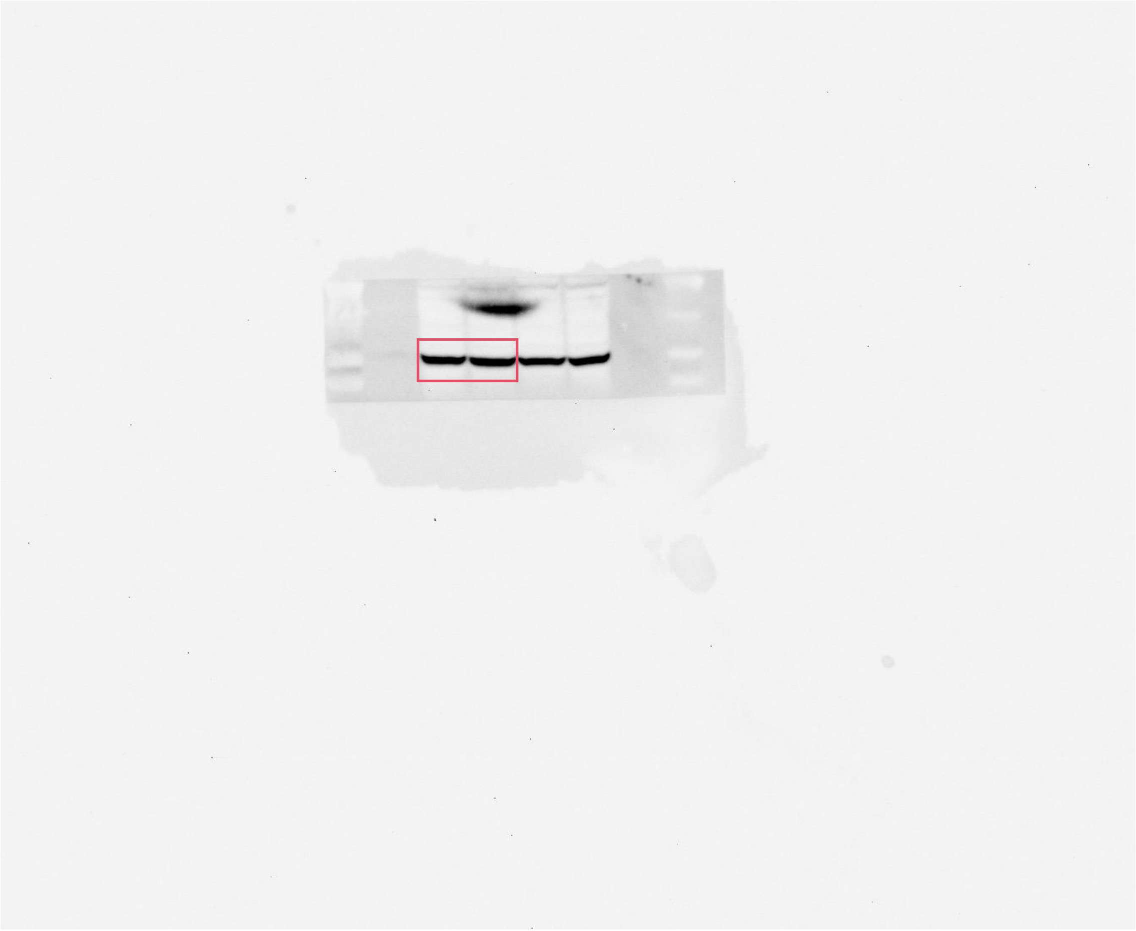

Figure S2A

FTL

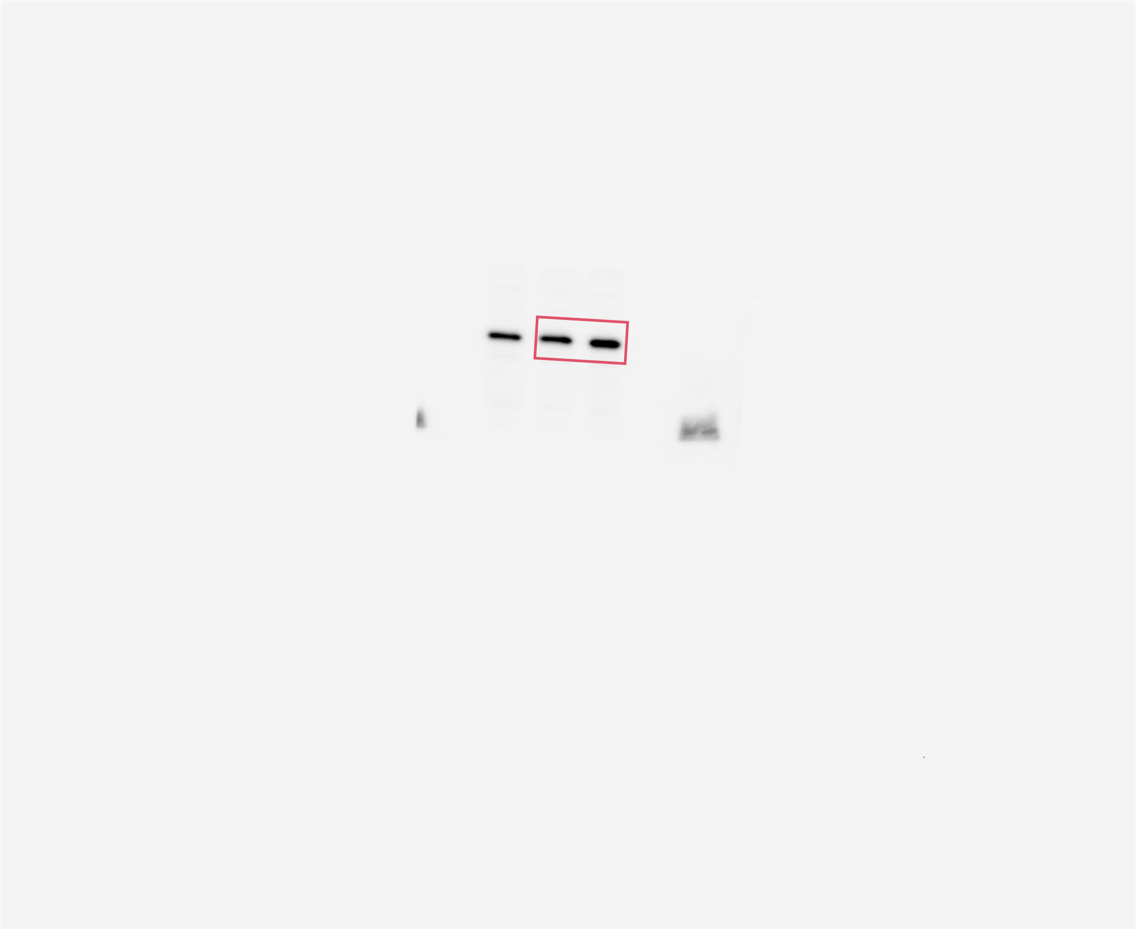

Figure S2A

GAPDH

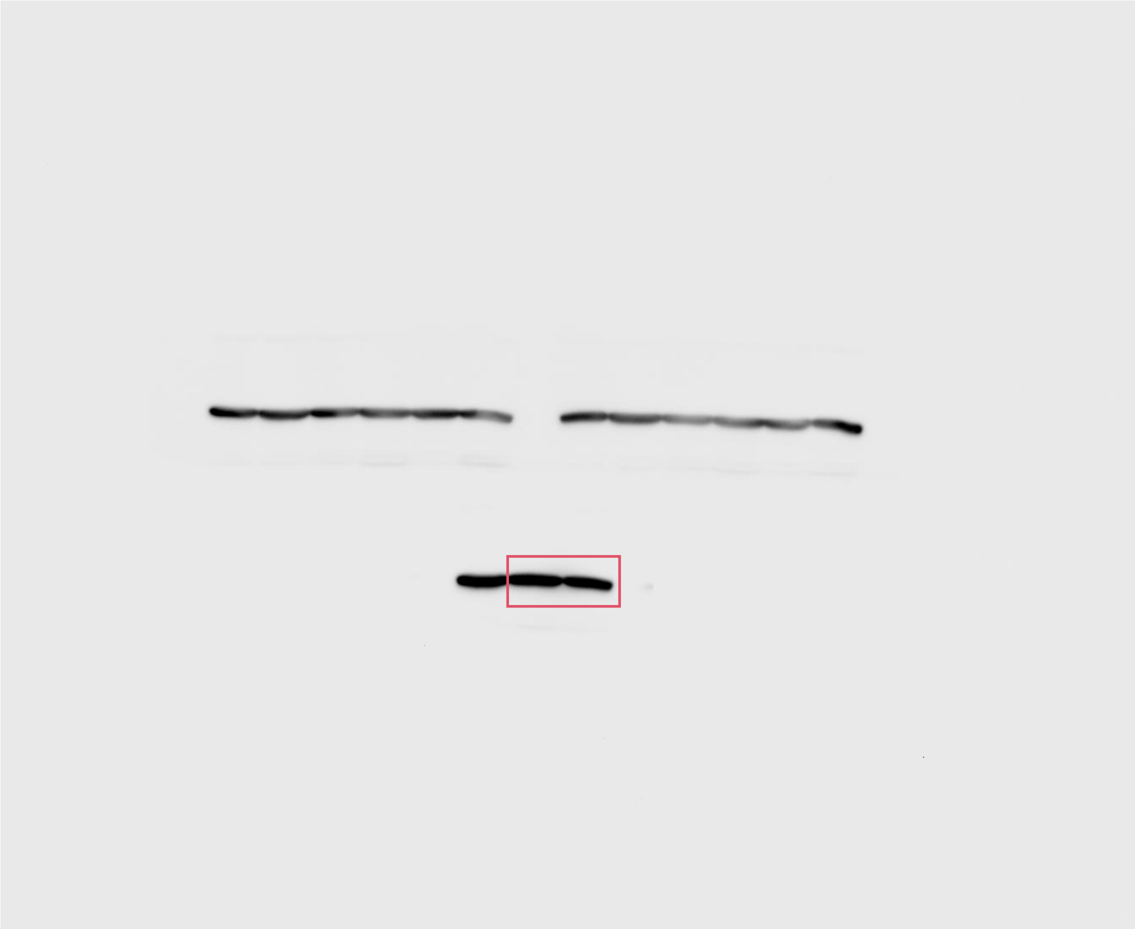

Figure S2B

ATG5

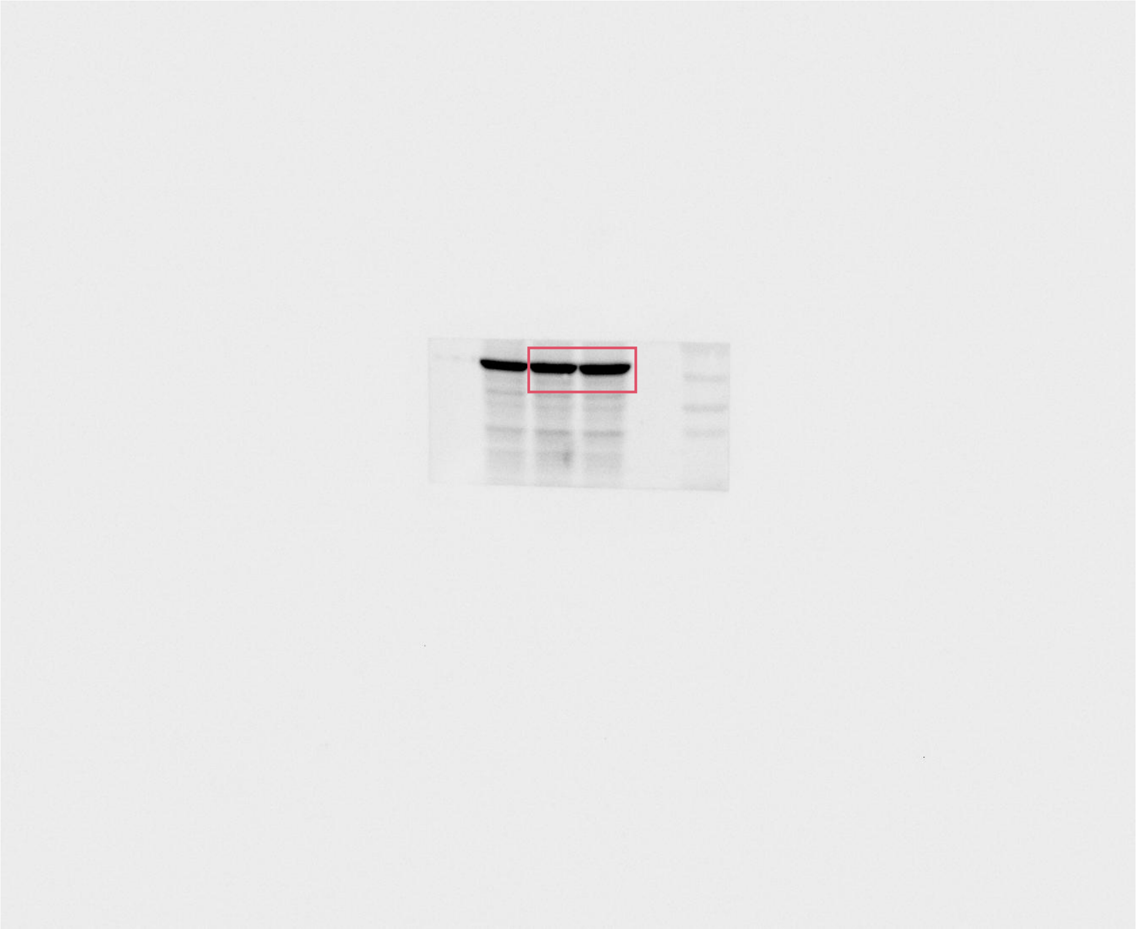

Figure S2B

p62

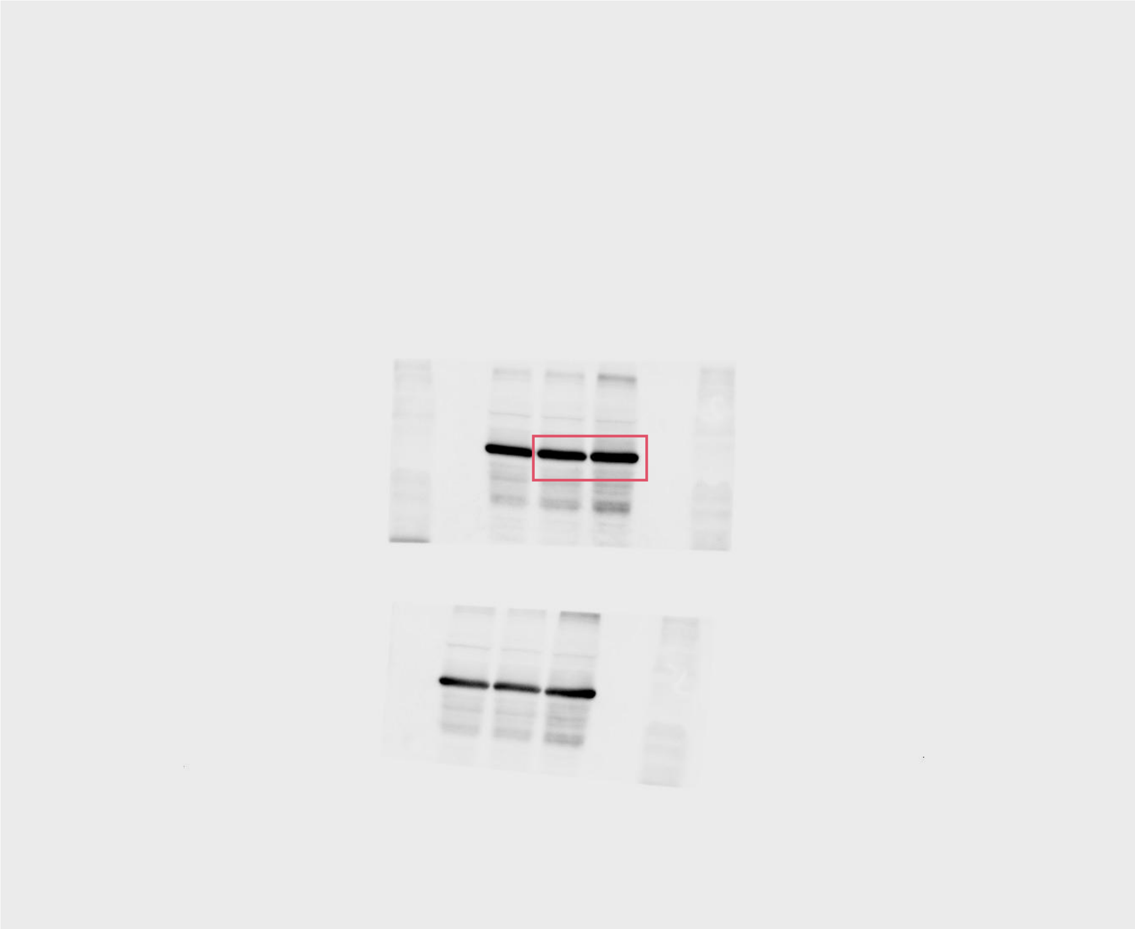

Figure S2B

LC3

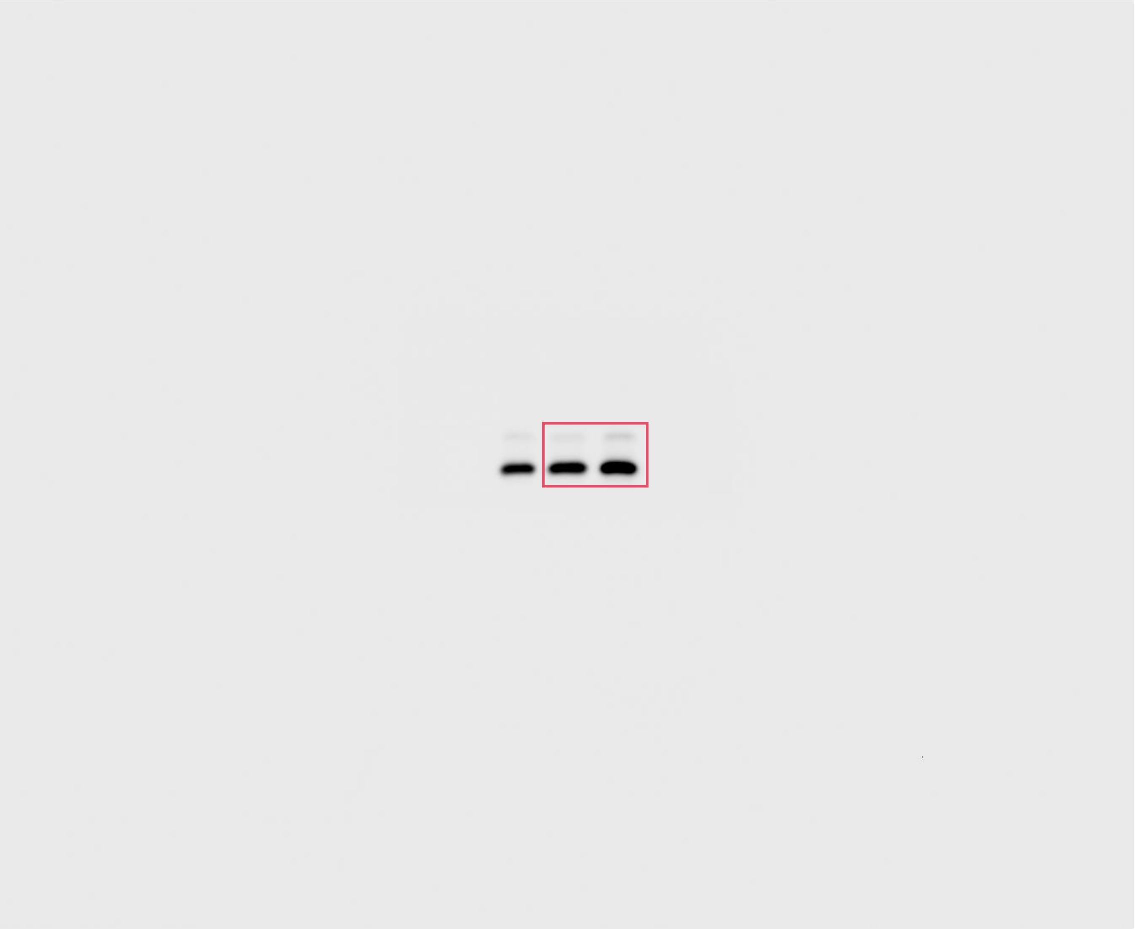

Figure S2B

TUBULIN

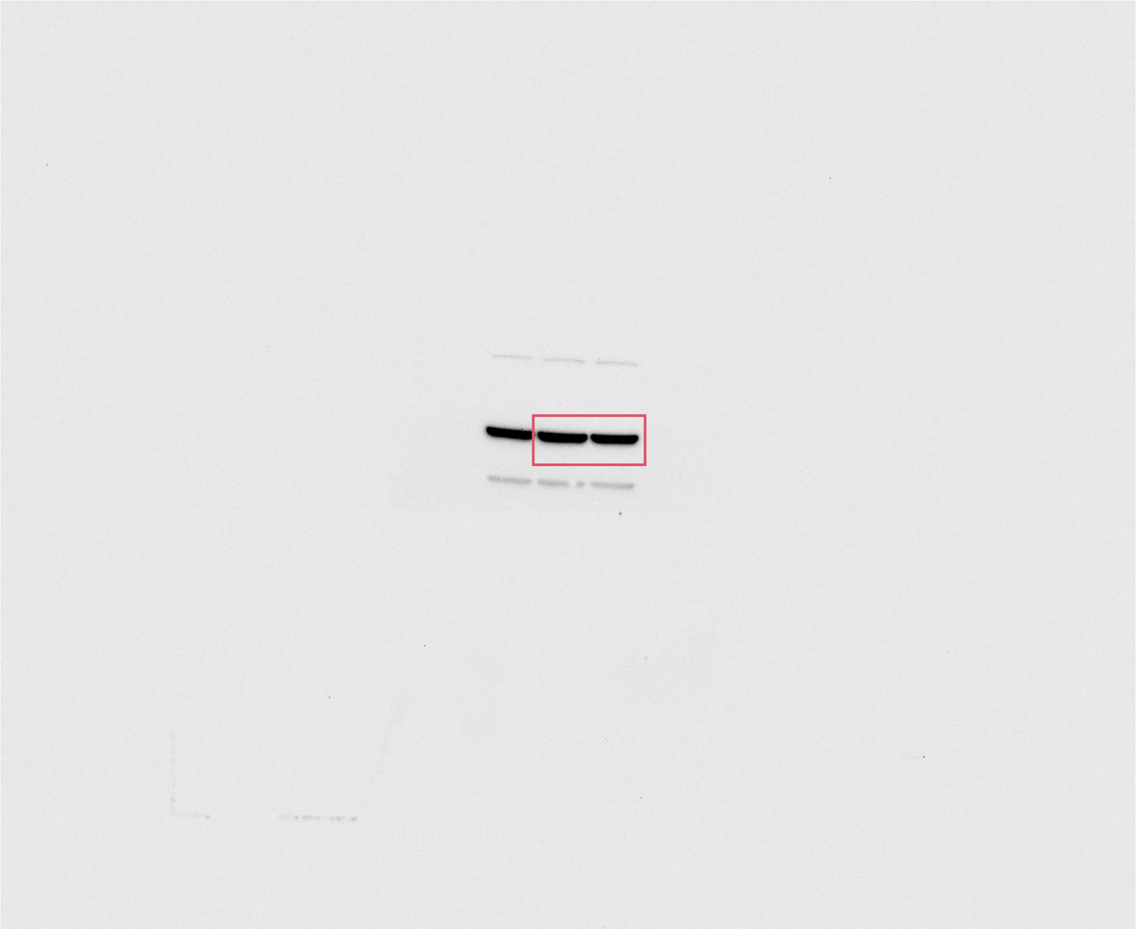

Figure S2C

IB-PTPMT1

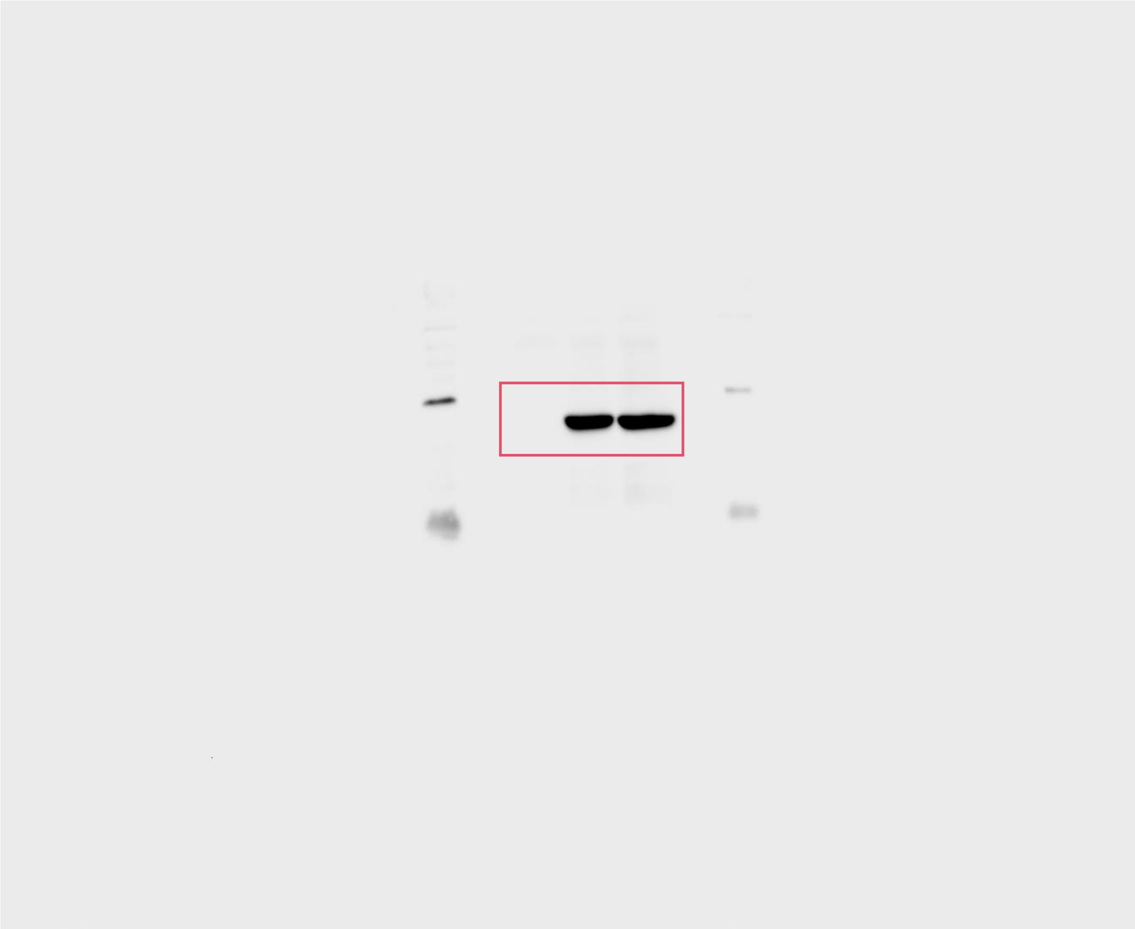

Figure S2C

IB-FTL

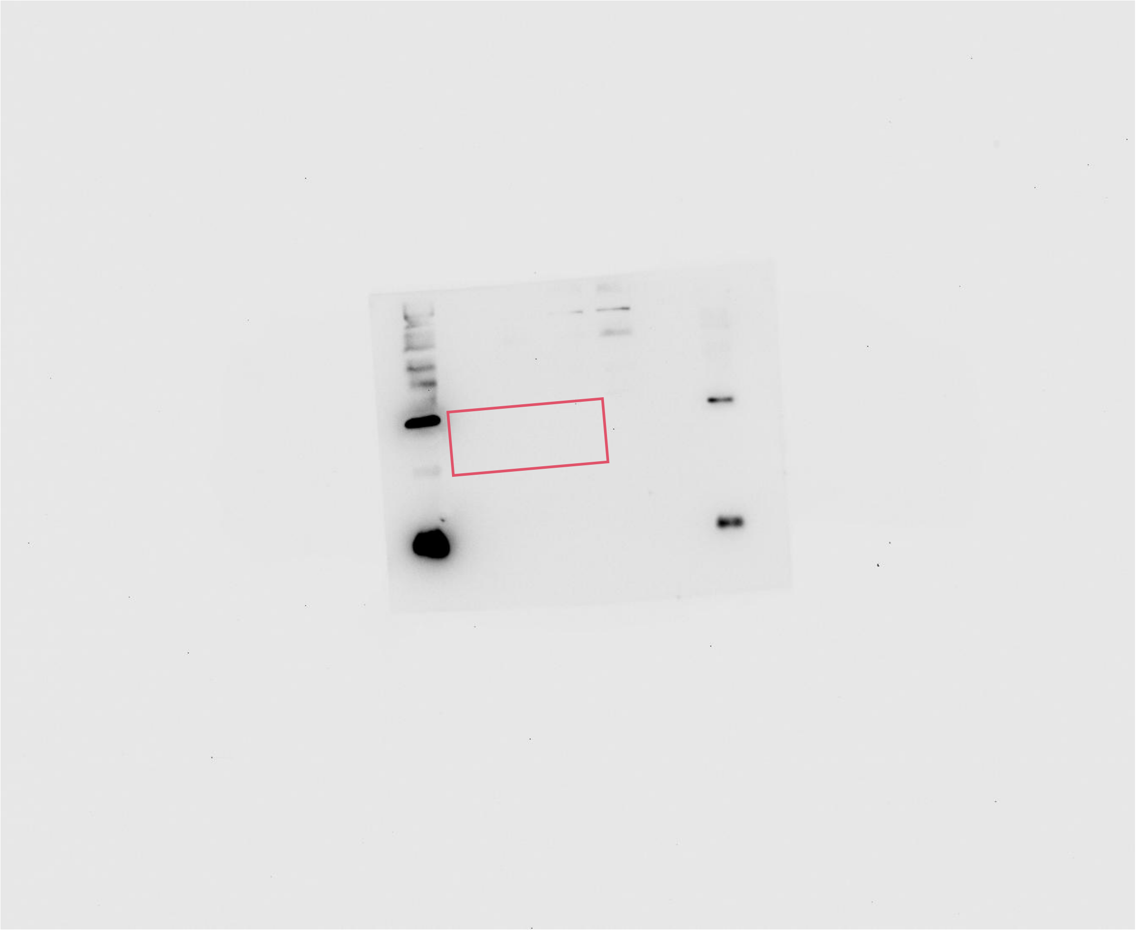

Figure S2C

Total-PTPMT1

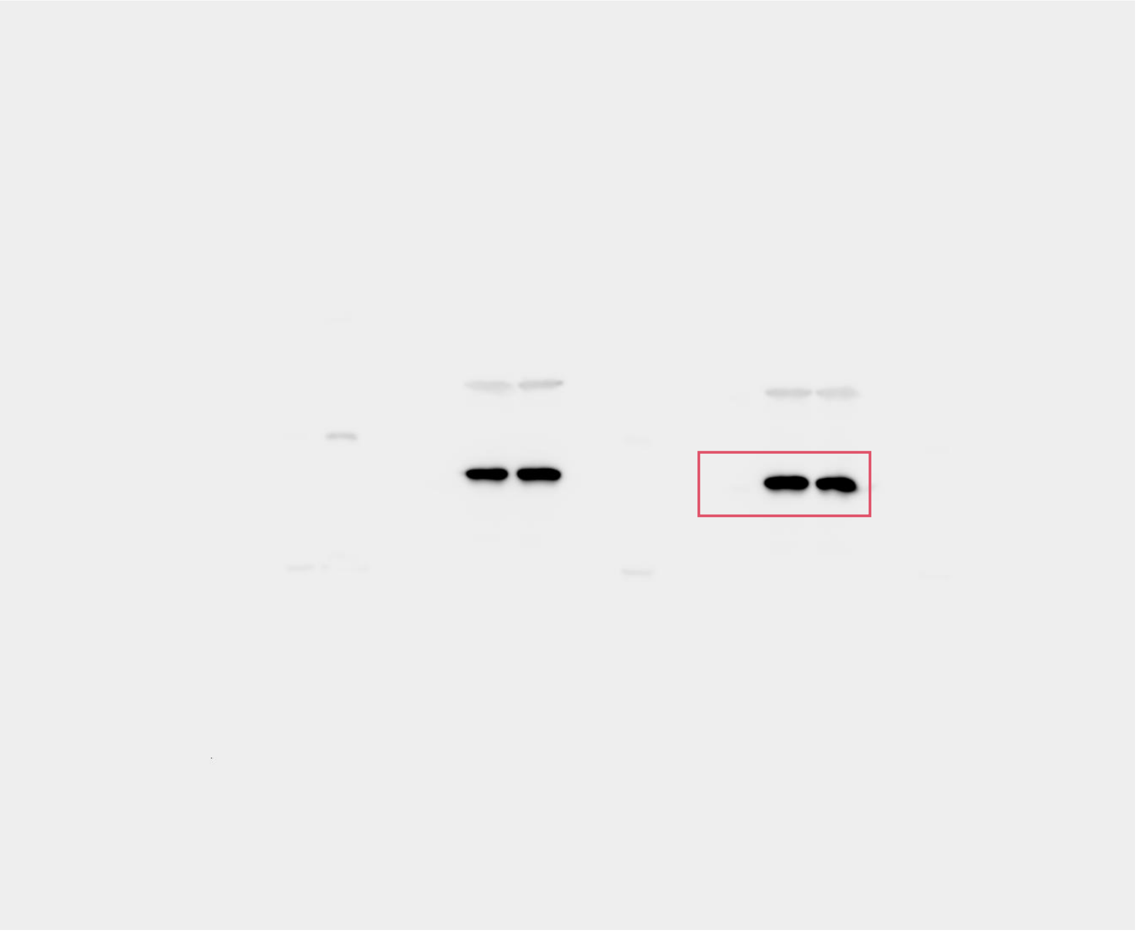

Figure S2C

Total-FTL

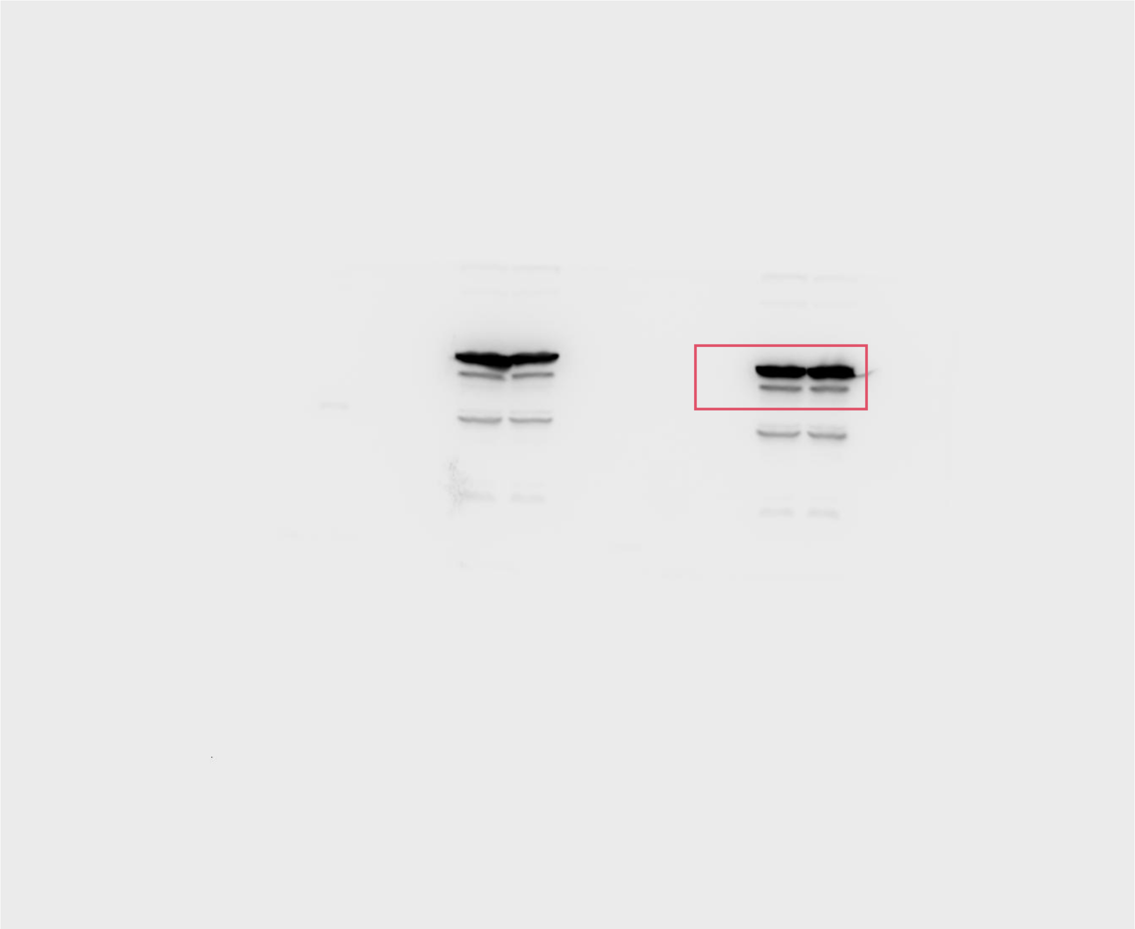

Figure S2C

Total-GAPDH

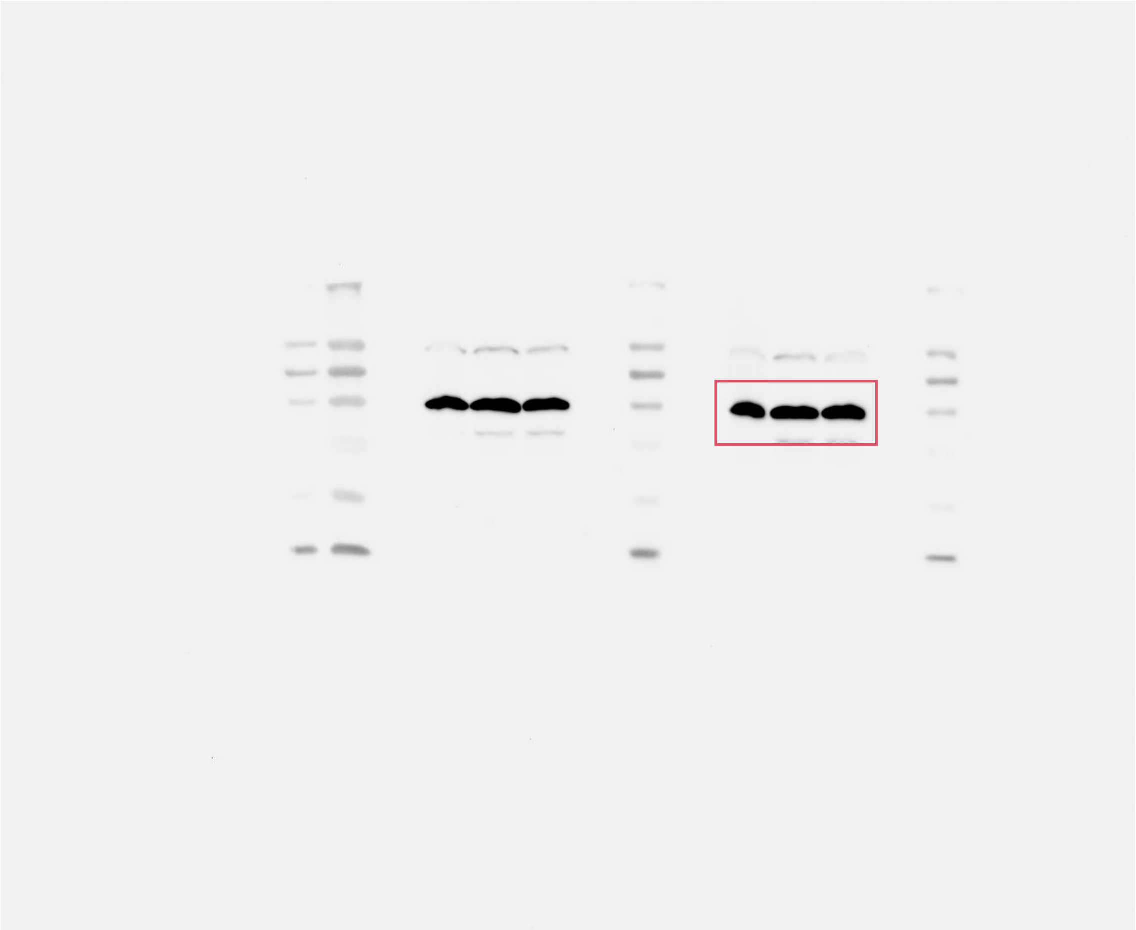

Figure S3A

PTPMT1

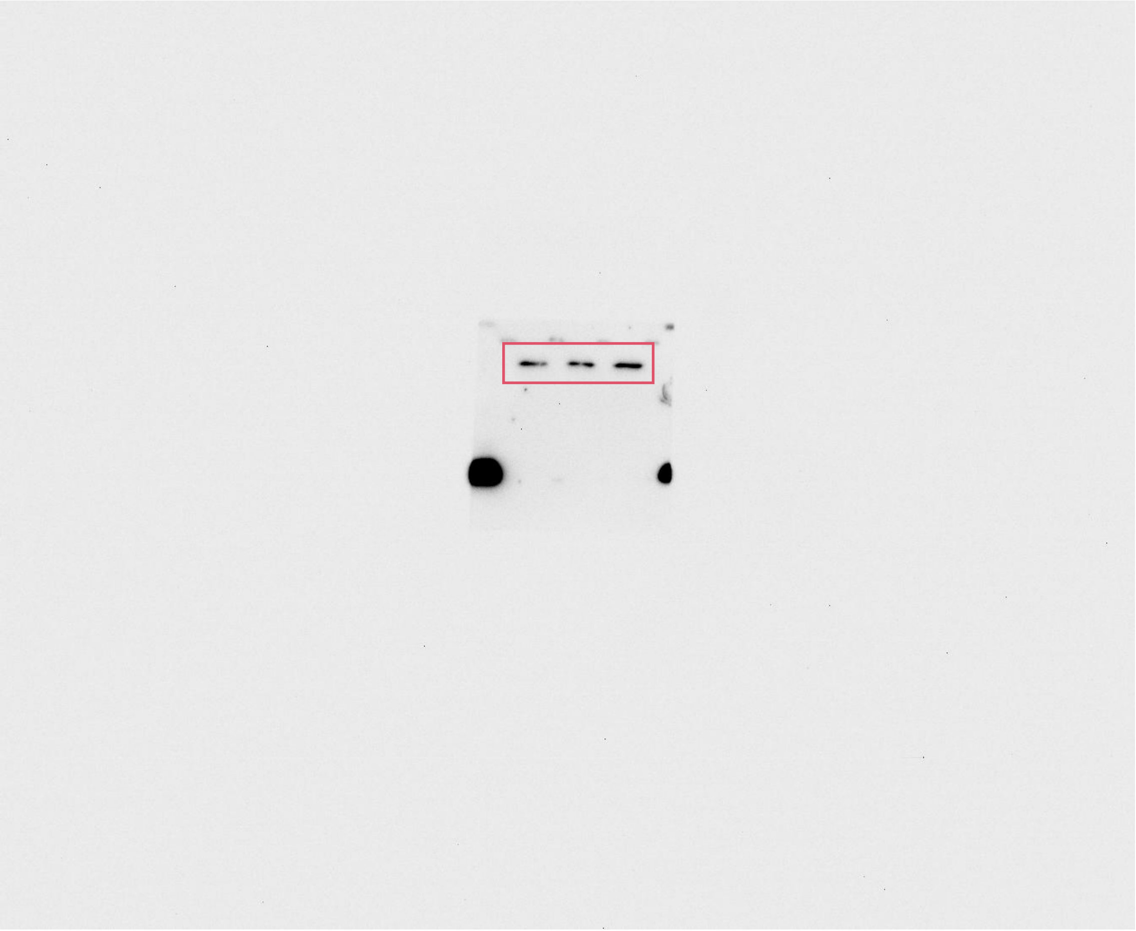

Figure S3A

TUBULIN

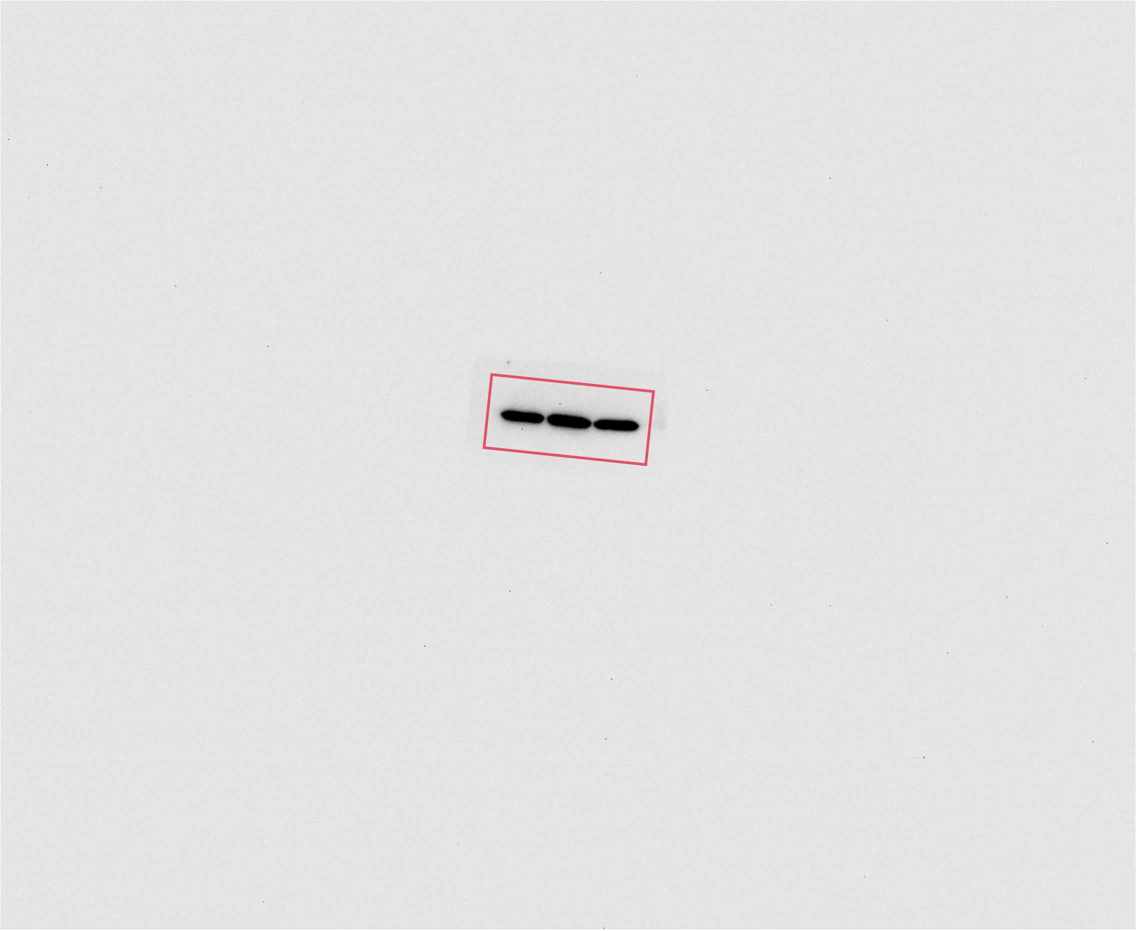

Figure S3B

PTPTM1

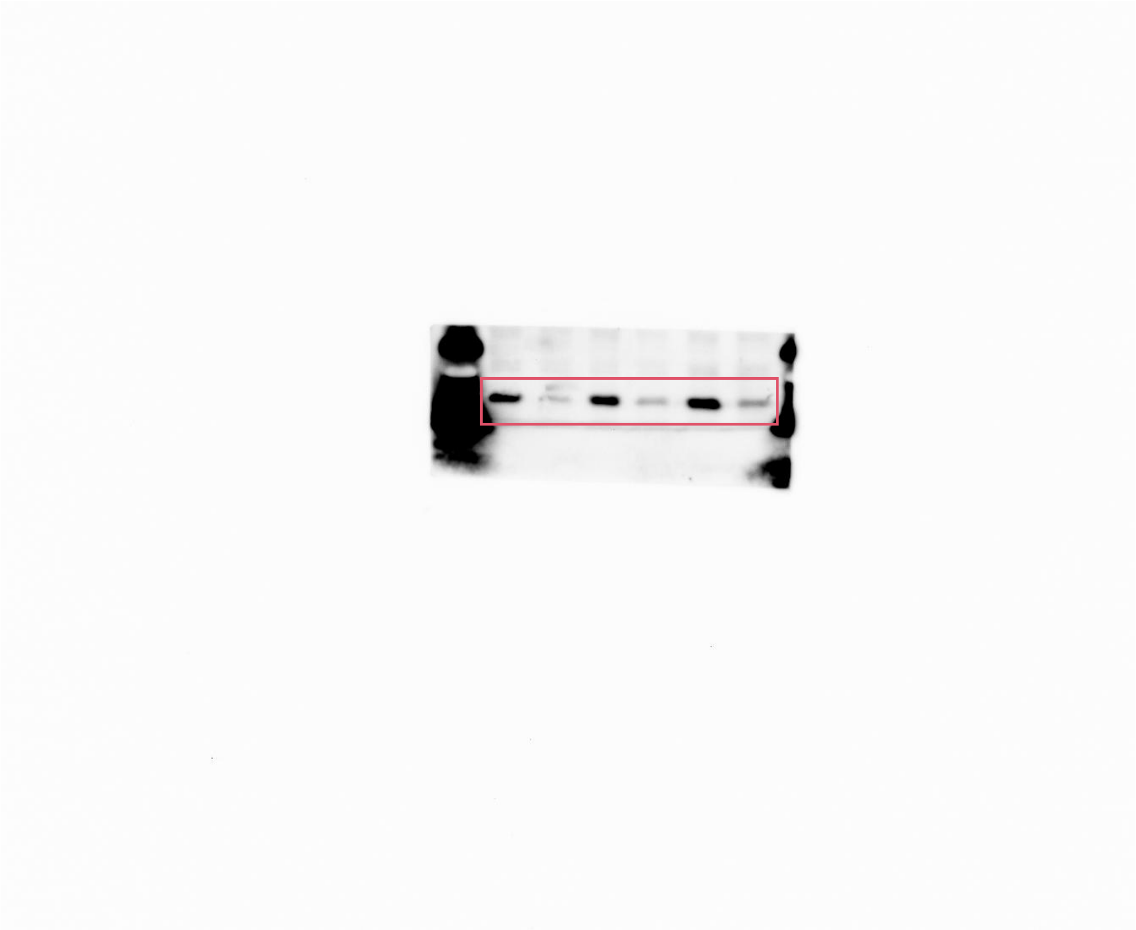

Figure S3B

TUBULIN

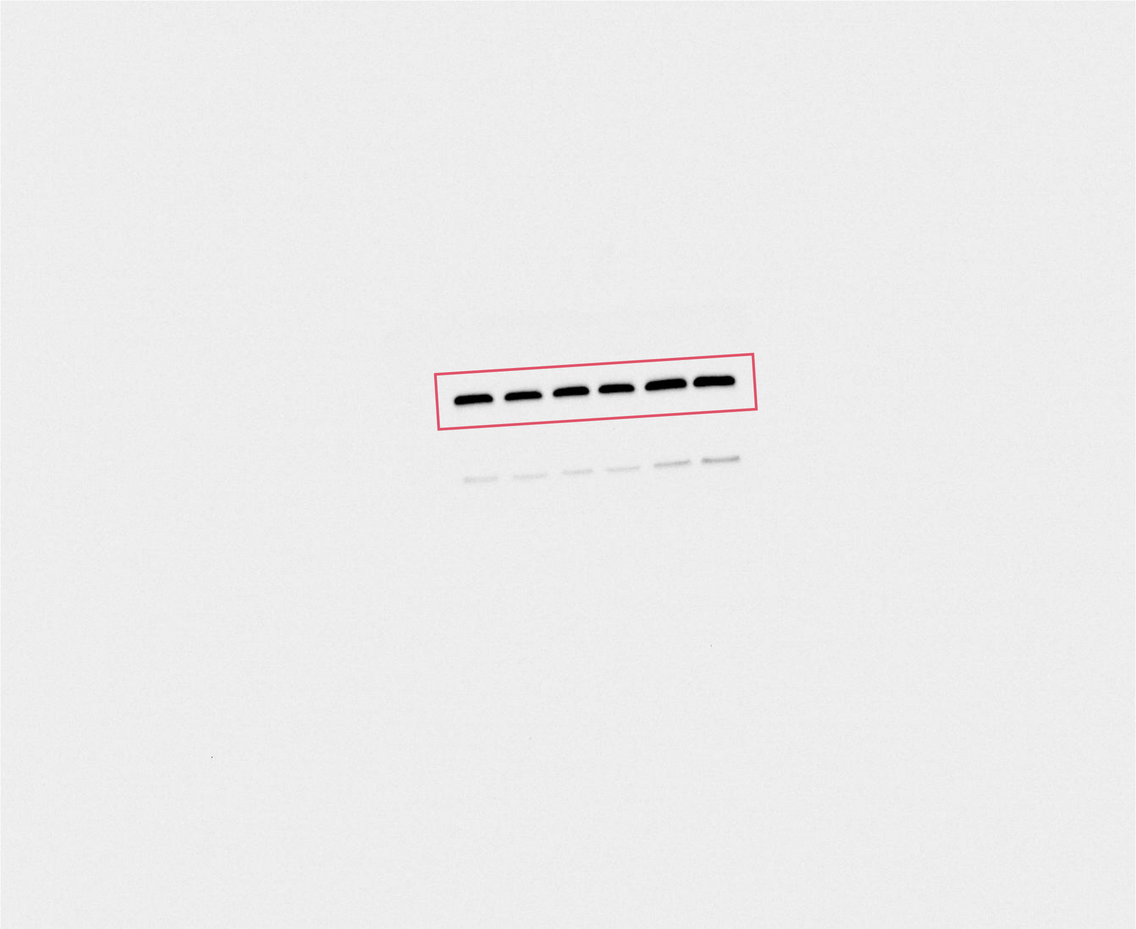

Figure S3C

Ub

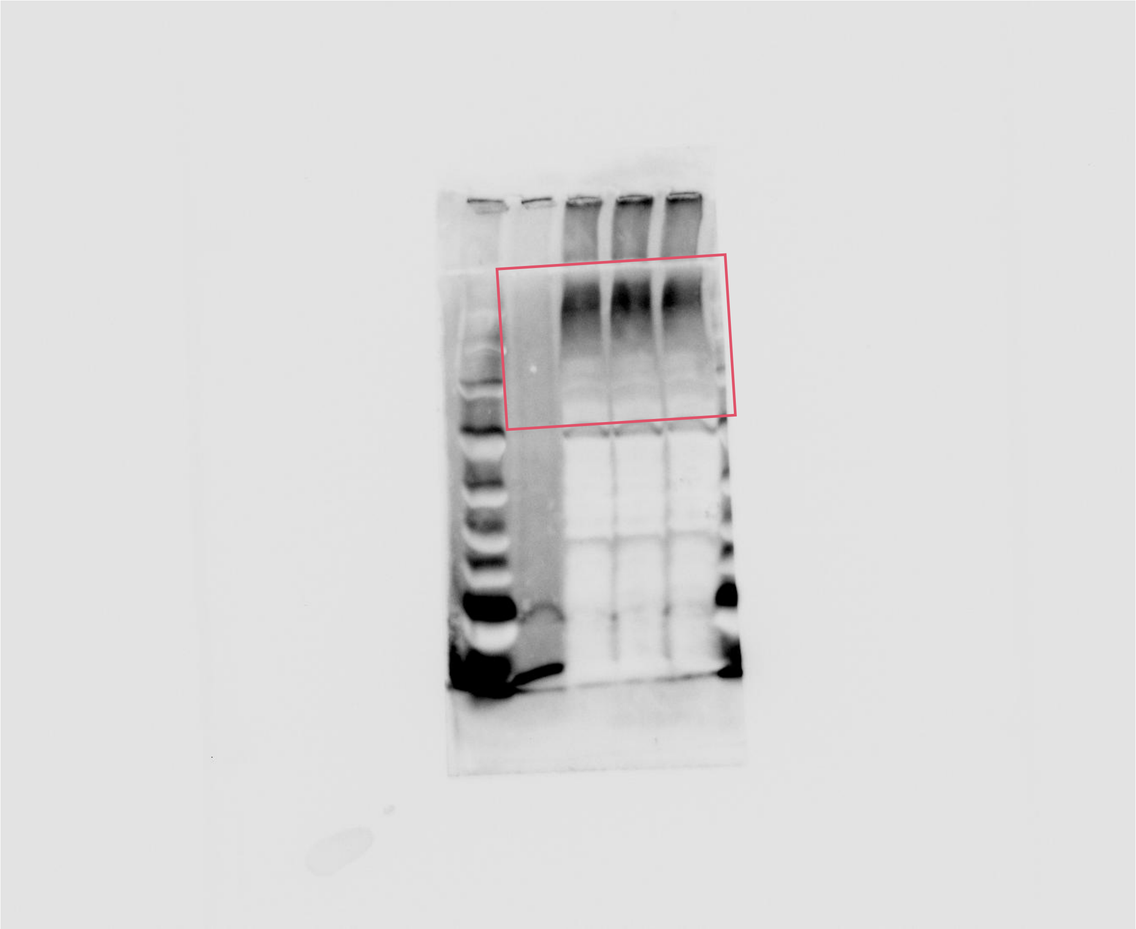

Figure S3C

PTPMT1

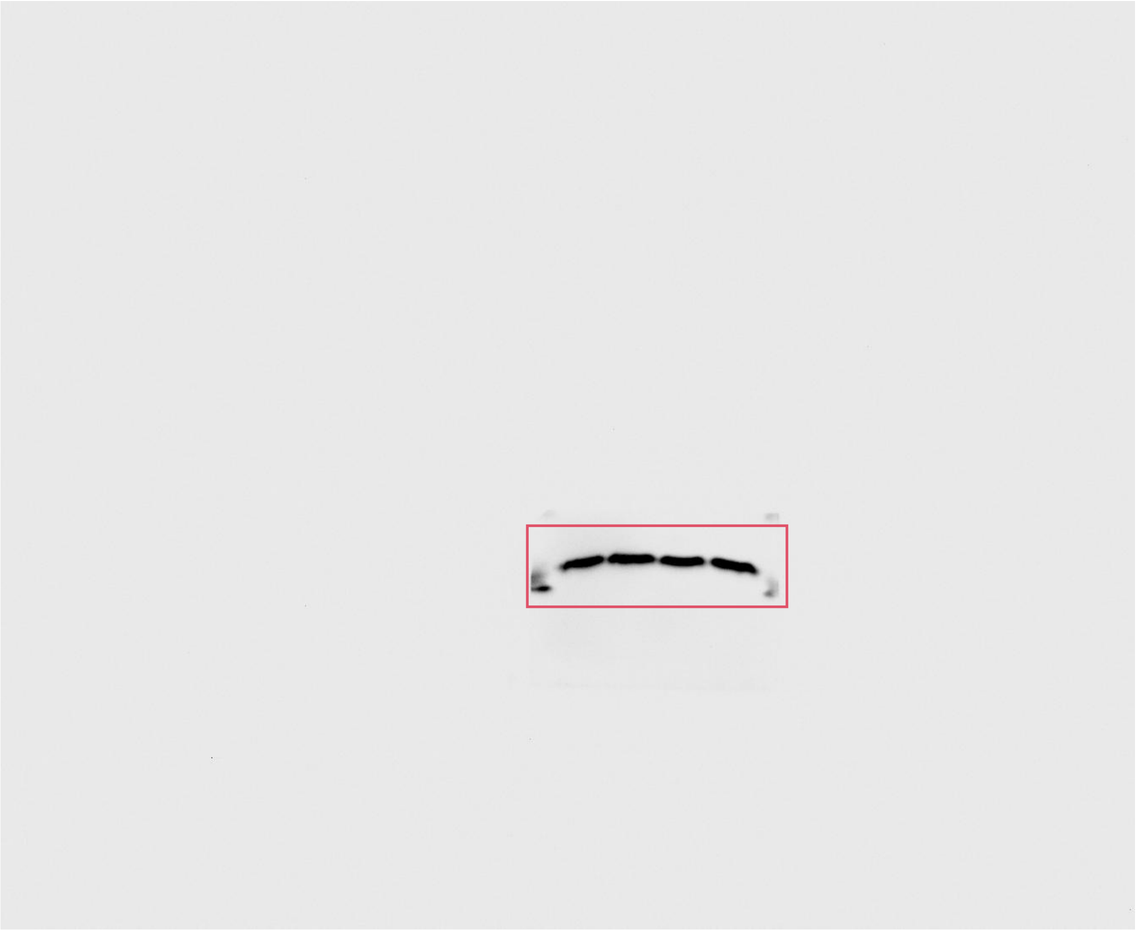

Figure S3C

GAPDH

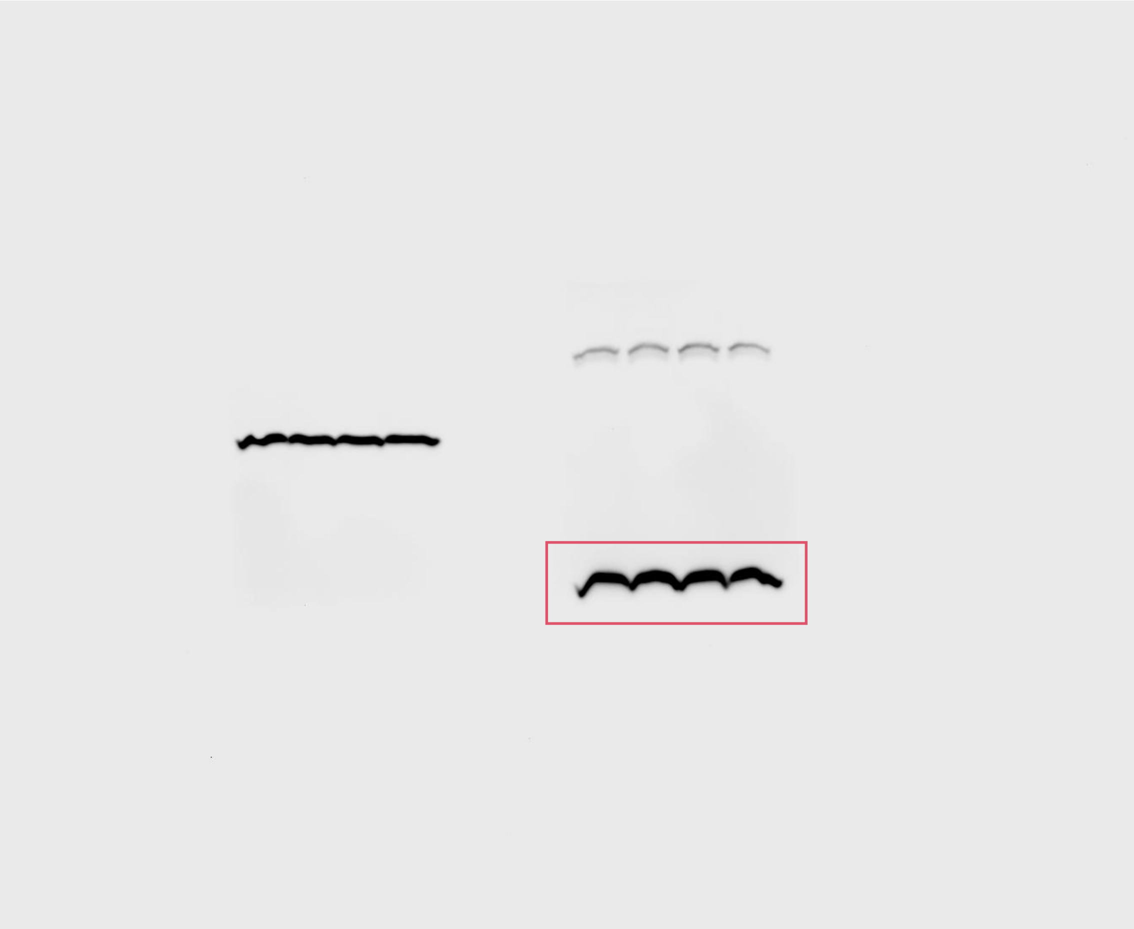

Figure S3D

PTPMT1

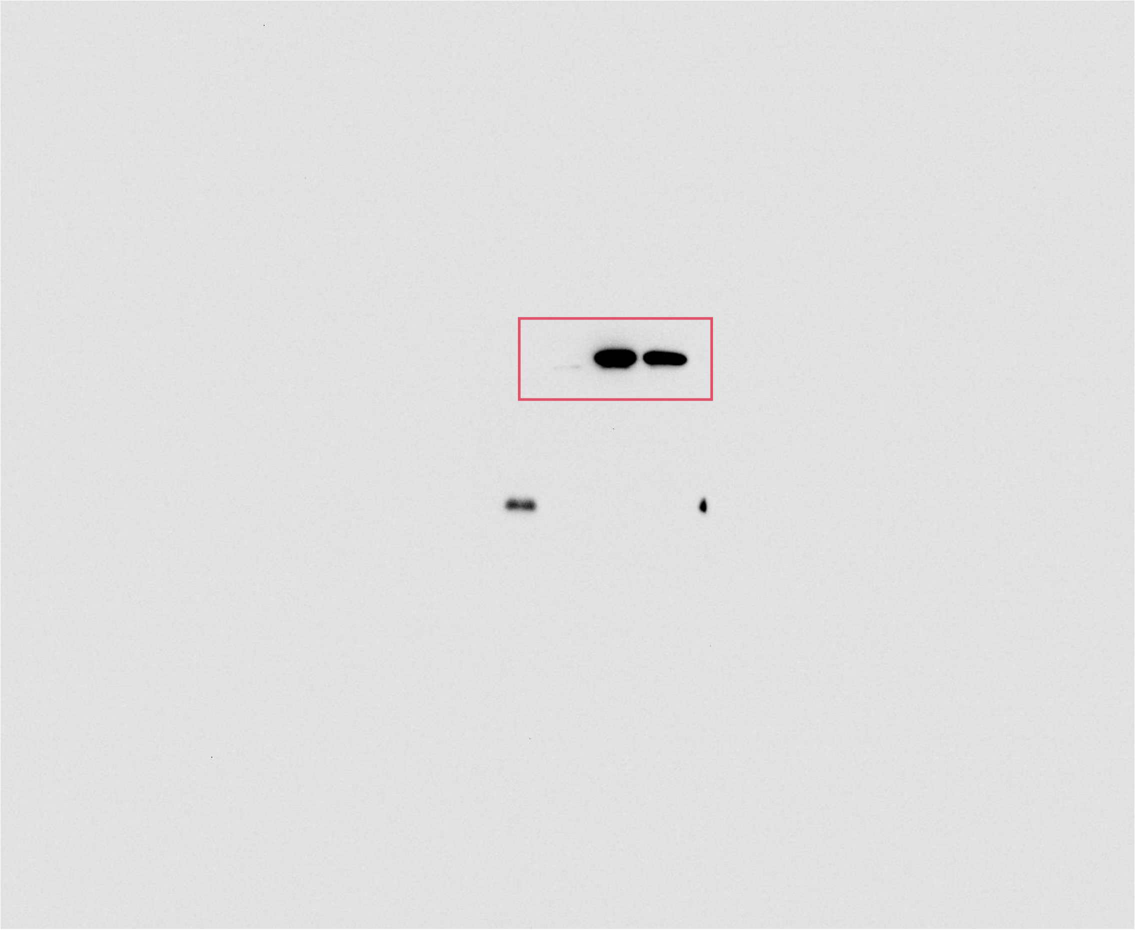

Figure S3D

TUBULIN

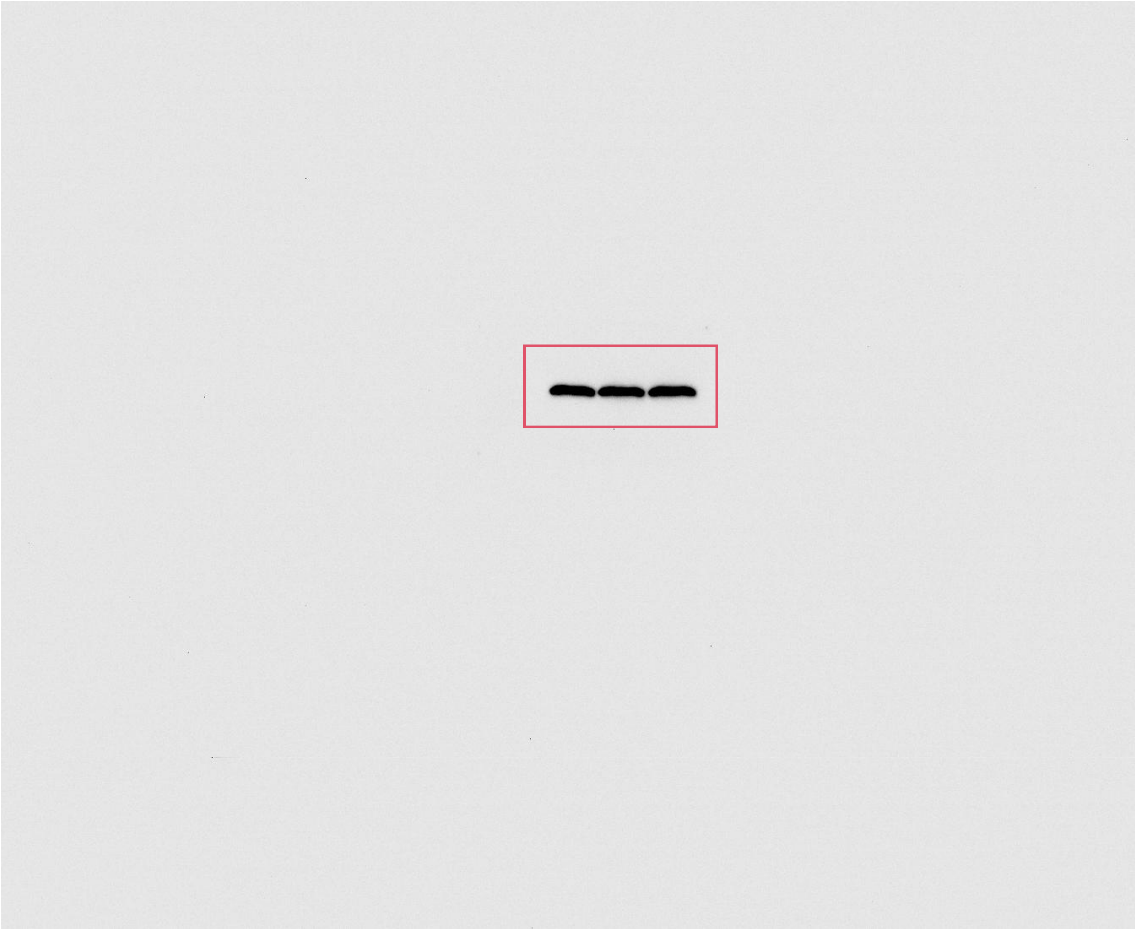

Figure S4B

PTPMT1

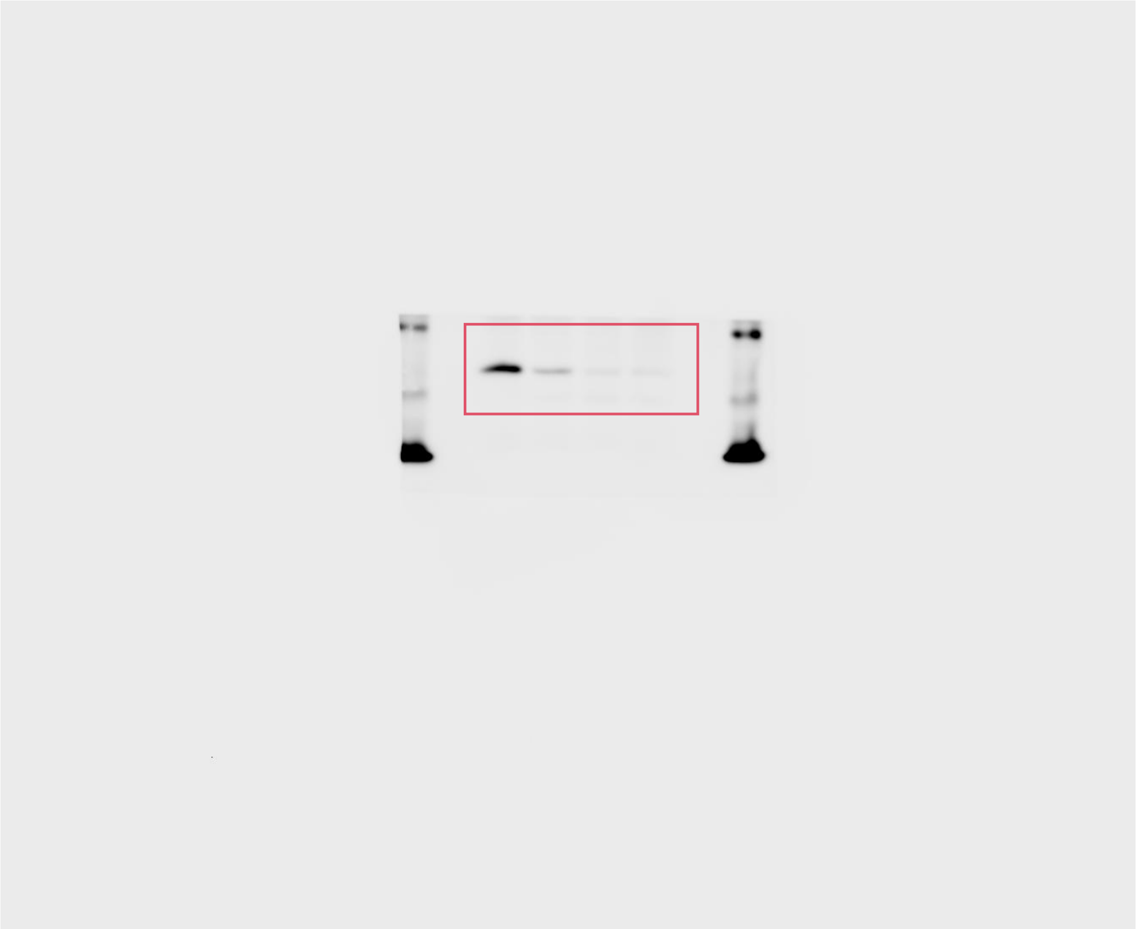

Figure S4B

FTL

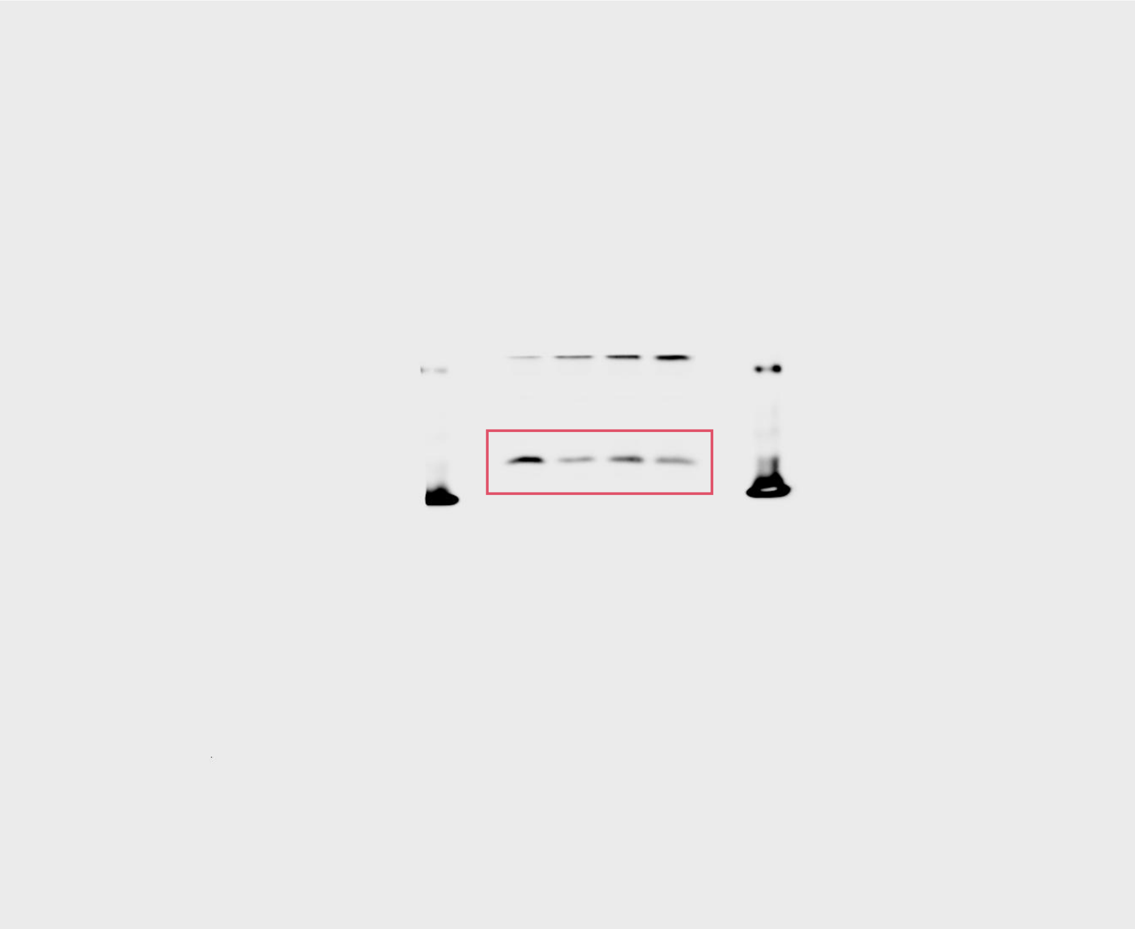

Figure S4A

TUBULIN

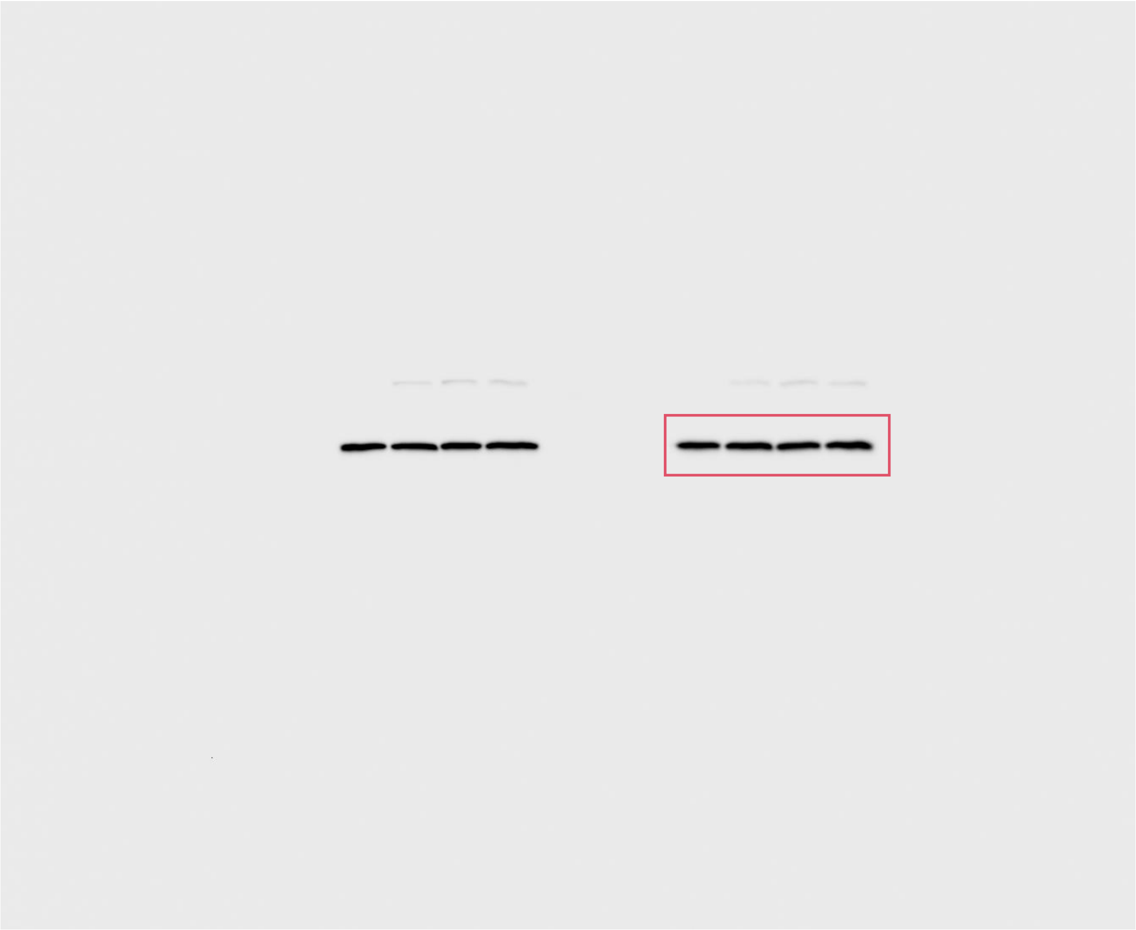

Figure S5D

DRP1

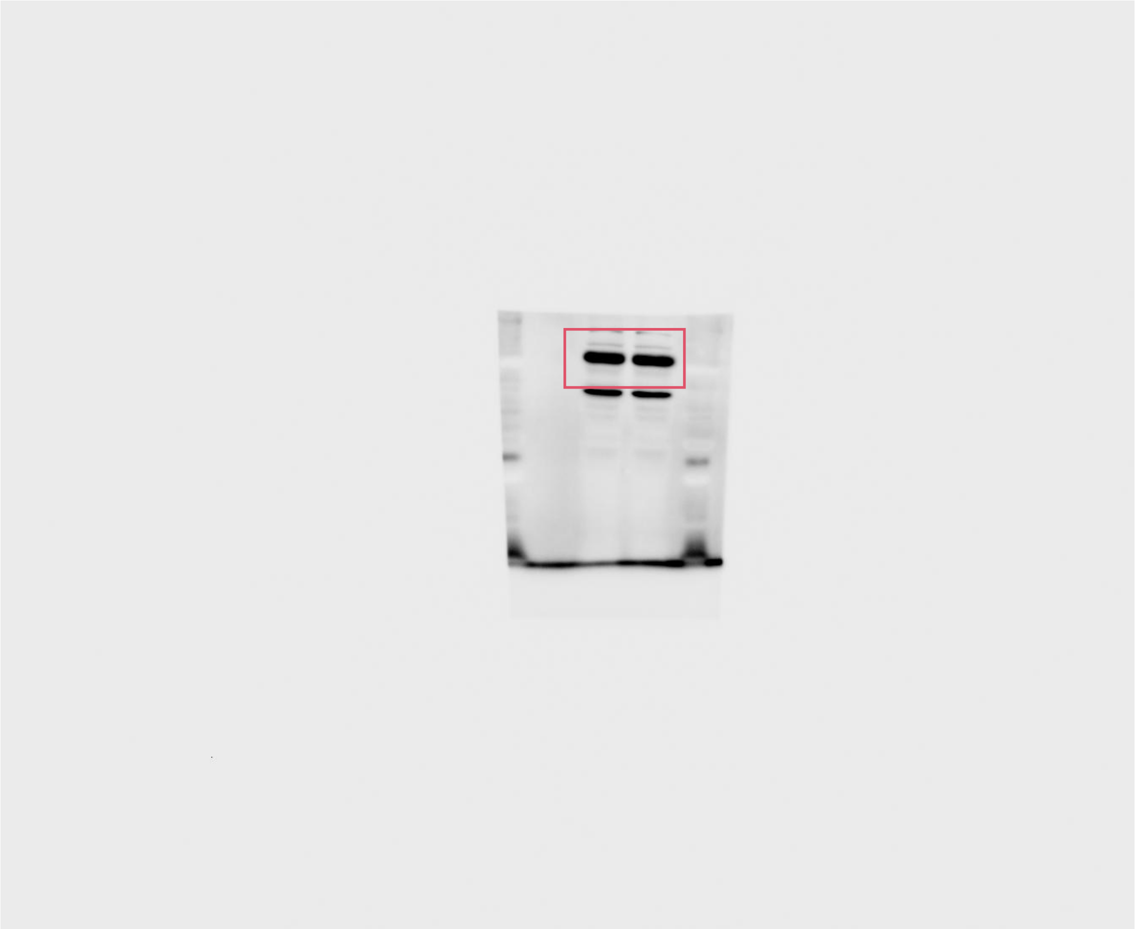

Figure S5D

MFN1

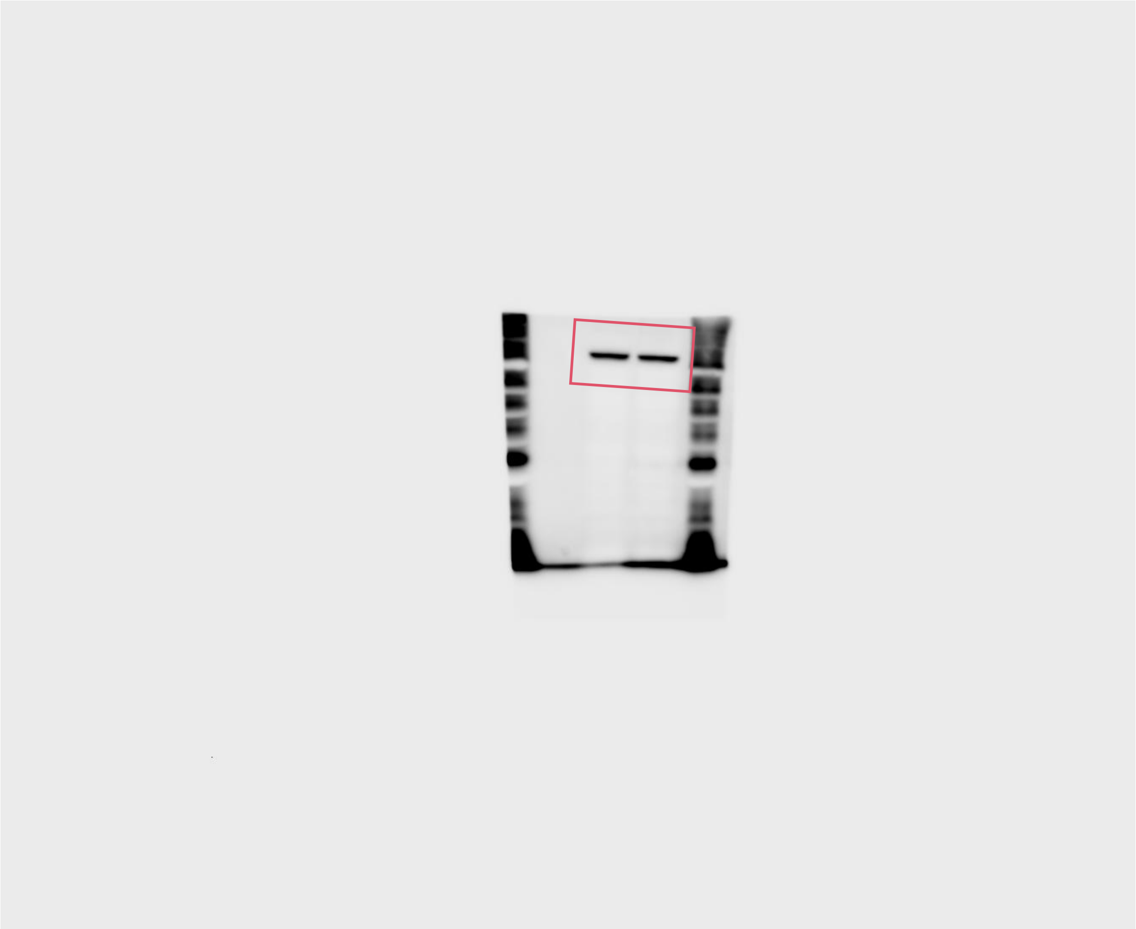

Figure S5D

TUBULIN

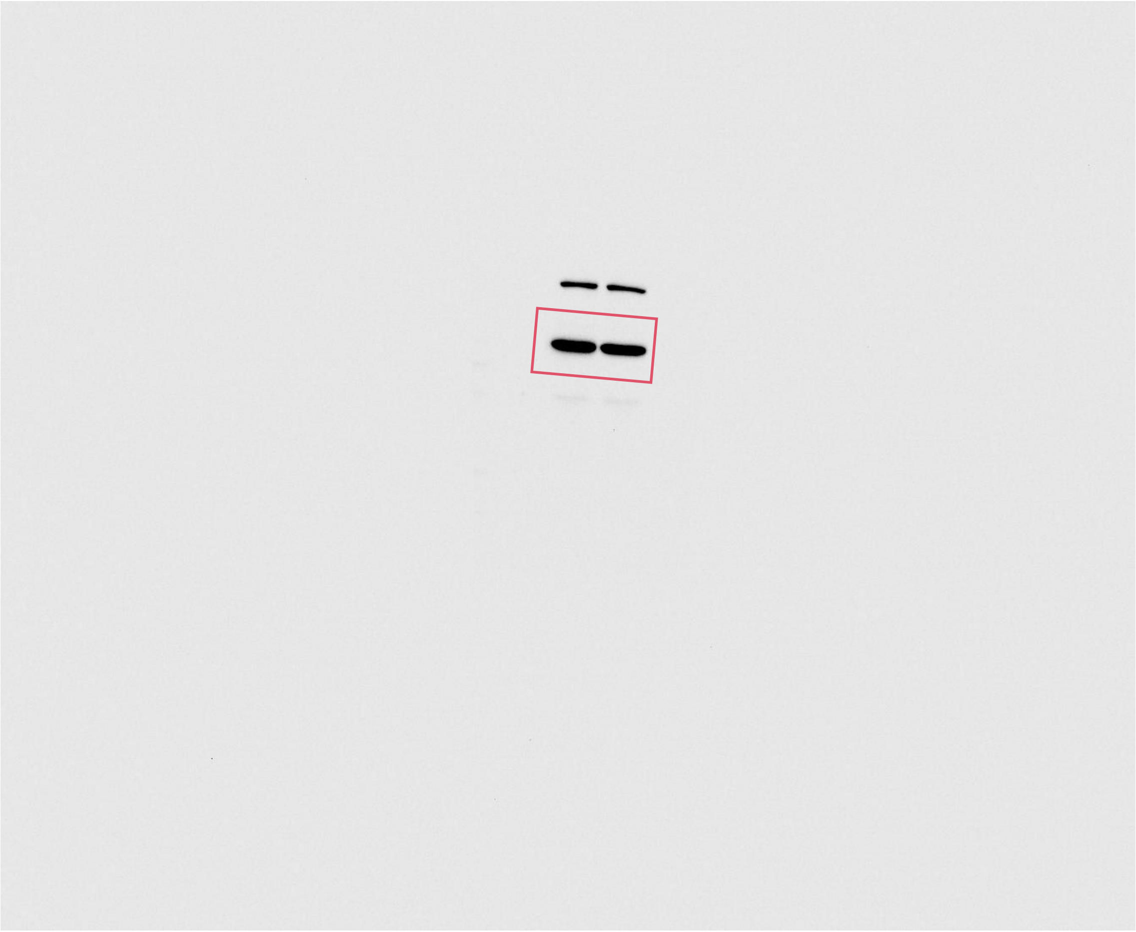

Figure S7B

CASPASE3

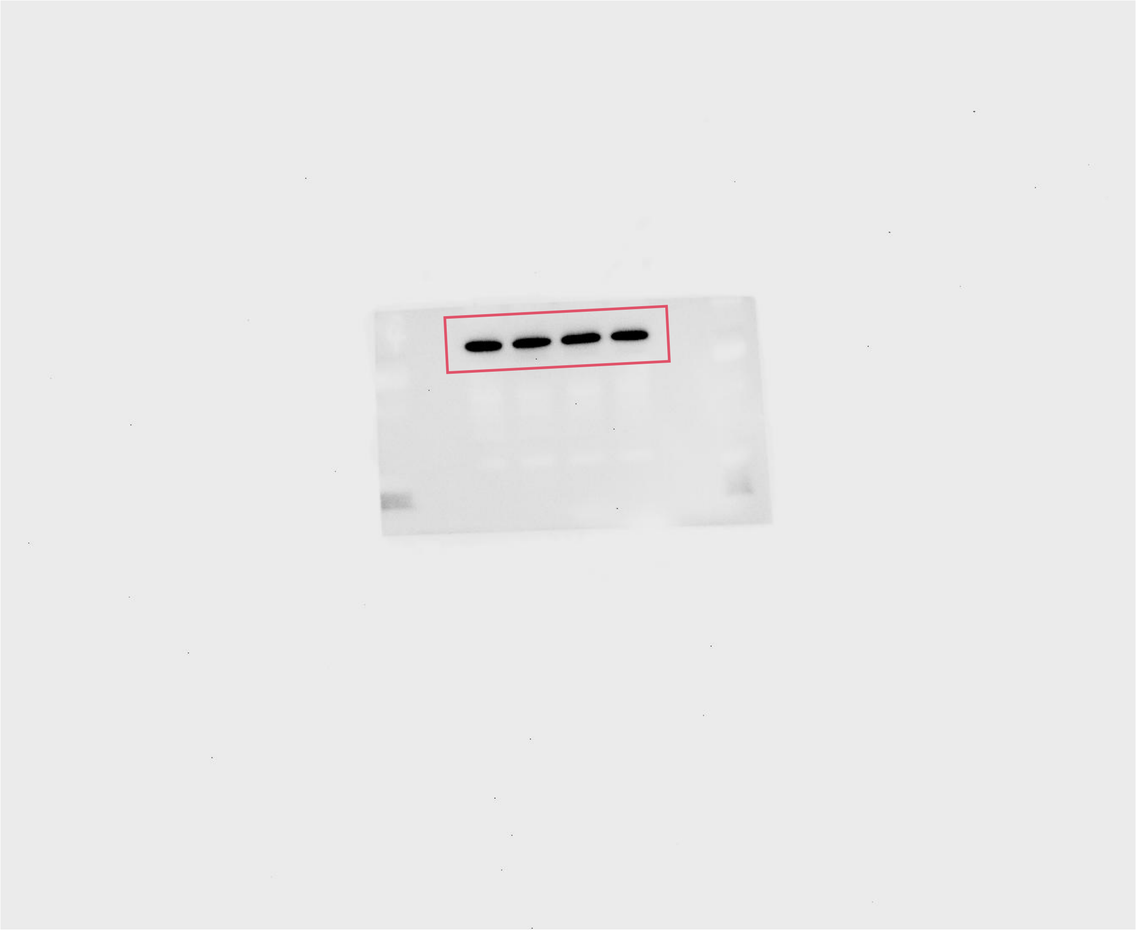

Figure S7B

BCL2

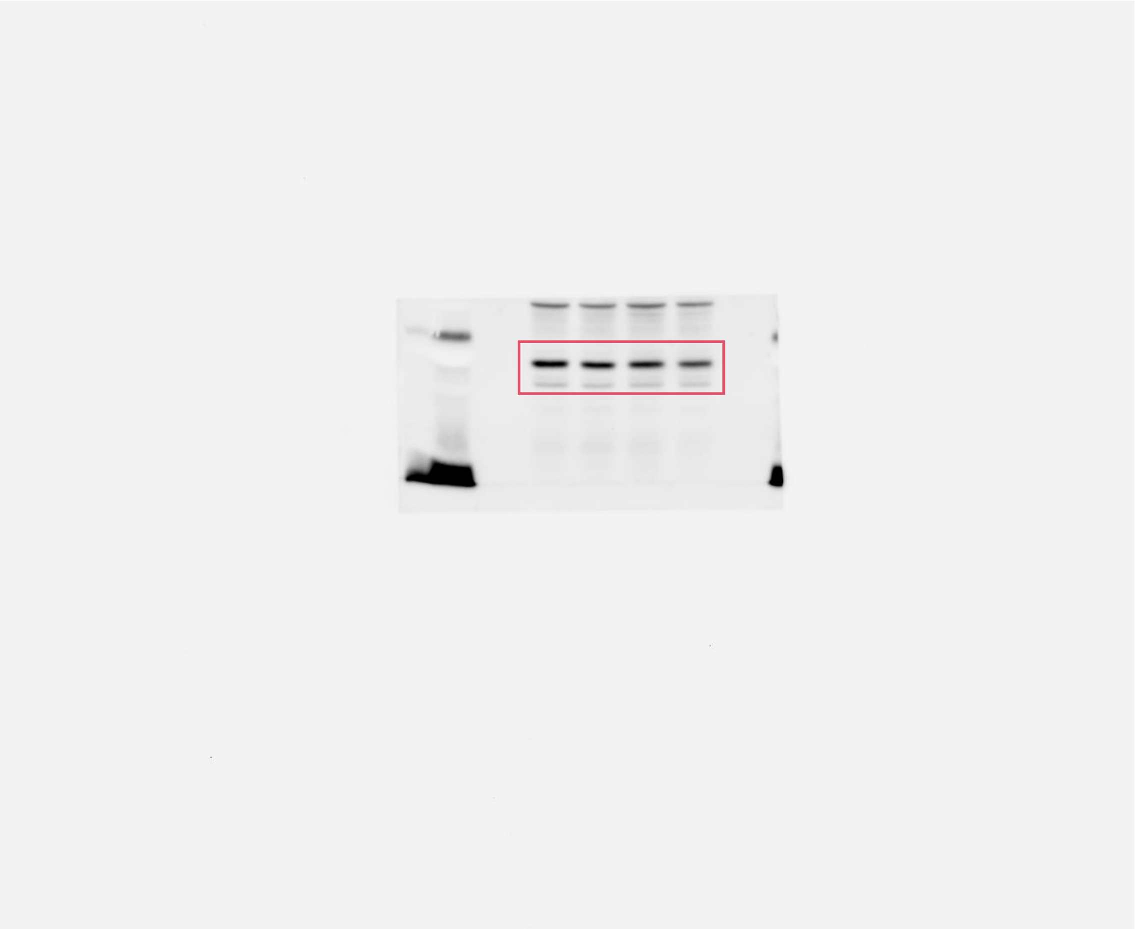

Figure S7B

TUBULIN

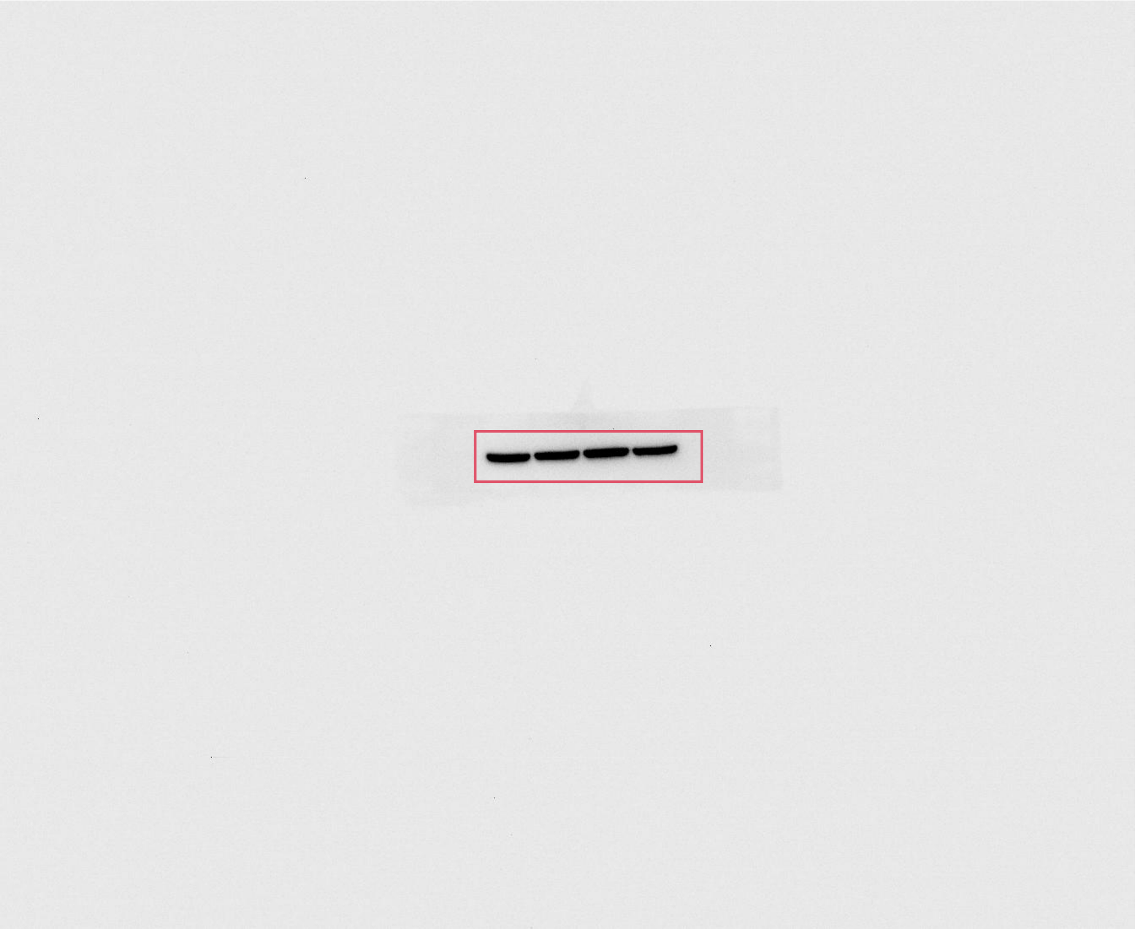

Supplement: Supplementary file 1 — The uncropped raw images of western blot [file 41419_2025_7581_MOESM1_ESM.pdf]
