# Supplementary material for: Pharmacological targeting of the mitochondrial phosphatase PTPMT1 sensitizes hepatocellular carcinoma to ferroptosis
Source: Cell Death Dis. 2025 Apr 6;16(1):257. doi: 10.1038/s41419-025-07581-5 (PMC11973169; doi:10.1038/s41419-025-07581-5)
Supplement: Supplementary file 2 — Supplementary Materials [file 41419_2025_7581_MOESM2_ESM.pdf]

Supplementary Materials for

Pharmacological targeting of the mitochondrial phosphatase PTPMT1 sensitizes hepatocellular carcinoma to ferroptosis

Miaomiao Li[1,2,3], Yi Wang[3], Xinyan Li[2], Jiayi Xu[2], Liangwen Yan[2], Shenkang Tang[2,4], Chenyue Liu[5], Mengjiao Shi[2,6], Rongrong Liu[2], Yaping Zhao[2], Yi Zhang[2], Lan Yang[2], Yinggang Zhang[2], Gang Wang[1,7], Zongfang Li[8,6], Ying Guo[2,6*], Yetong Feng[2,9*], Pengfei Liu[2,6,10*]

1. Department of Critical Care Medicine, National & Local Joint Engineering Research Center of Biodiagnosis and Biotherapy, The Second Affiliated Hospital of Xi'an Jiaotong University, Xi'an, China
2. International Joint Research Center on Cell Stress and Disease Diagnosis and Therapy, National & Local Joint Engineering Research Center of Biodiagnosis and Biotherapy, The Second Affiliated Hospital of Xi'an Jiaotong University, Xi'an, China
3. Department of Regenerative Medicine, School of Pharmaceutical Science, Jilin University, Changchun, China
4. Department of Oncology, Affiliated Hospital of Shaanxi University of Chinese Medicine, Xianyang, China
5. Department of Medical Image, The First Affiliated Hospital of Xi'an Jiaotong University, Xi'an, China
6. Shaanxi Provincial Clinical Research Center for Hepatic & Splenic Diseases, The Second Affiliated Hospital of Xi'an Jiaotong University, Xi'an, China
7. Key Laboratory of Surgical Critical Care and Life Support, Xi'an Jiaotong University, Ministry of Education of China, Xi'an, China
8. Department of General Surgery, National & Local Joint Engineering Research Center of Biodiagnosis and Biotherapy, The Second Affiliated Hospital of Xi'an Jiaotong University, Xi'an, China
9. Core Research Laboratory, The Second Affiliated Hospital of Xi'an Jiaotong University, Xi'an, China.
10. Key Laboratory of Environment and Genes Related To Diseases, Xi'an Jiaotong University, Ministry of Education of China, Xi'an, China.

* Correspondence to:

Pengfei Liu, E-mail address: liupengfei@xjtu.edu.cn

Yetong Feng, Email address: fengyetong@xjtu.edu.cn

Ying Guo, Email address: guoying.2yuan@xjtu.edu.cn

Running title: PTPMT1 regulates HCC ferroptosis

Supplementary Figures and Legends

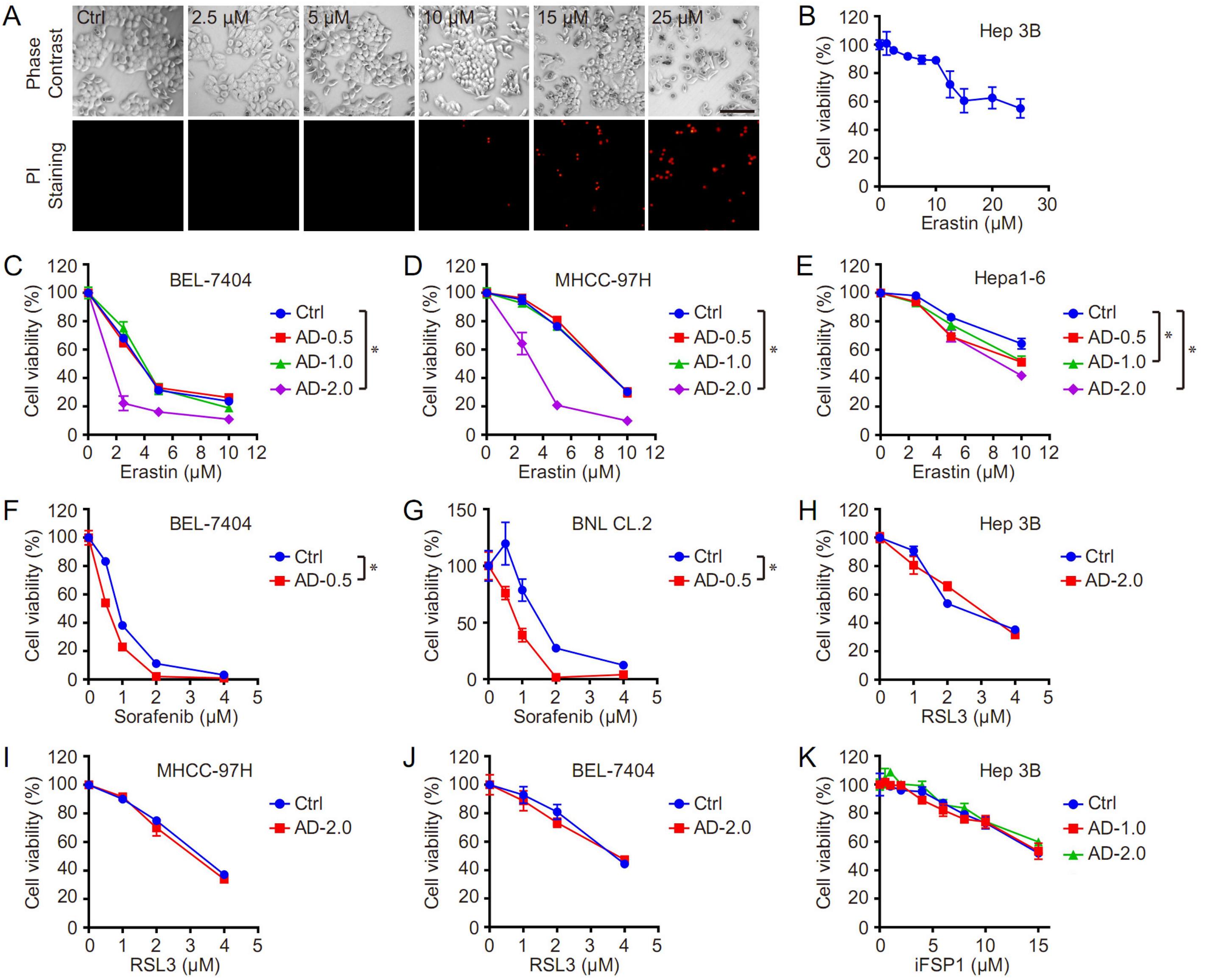

Figure S1. Pharmacological inhibition of PTPMT1 sensitizes HCC to cystine deprivation-induced ferroptosis. In Hep 3B cells, pharmacological inhibition of PTPMT1 was achieved via alexidine dihydrochloride (AD) treatment, while Erastin was employed to induce cystine deprivation-triggered ferroptosis. The Erastin-induced cytotoxicity was first evaluated in Hep 3B cells, and cell death (24-hour treatment) was tested using PI staining (A) and CCK-8 assay (B) respectively. Subsequently, the cell viability of BEL-7404 (C), MHCC-97H (D), and Hepa1-6 (E) for each treatment group was assessed using the CCK-8 assay. In parallel, cell viability of BEL-7404 (F) and BNL CL.2 (G) was also gauged in the Sorafenib-

induced cell death model. Ultimately, the sensitivity to RSL3 (a GPX4 inhibitor)-induced cell death (H-J) and iFSP1 (a FSP1 inhibitor)-induced cell death (K) was evaluated in both AD-treated and AD-untreated cells. Data are expressed as mean ± SD, and the P value less than 0.05 was considered statistically significant. *:$P < 0.05$ compared between two group.

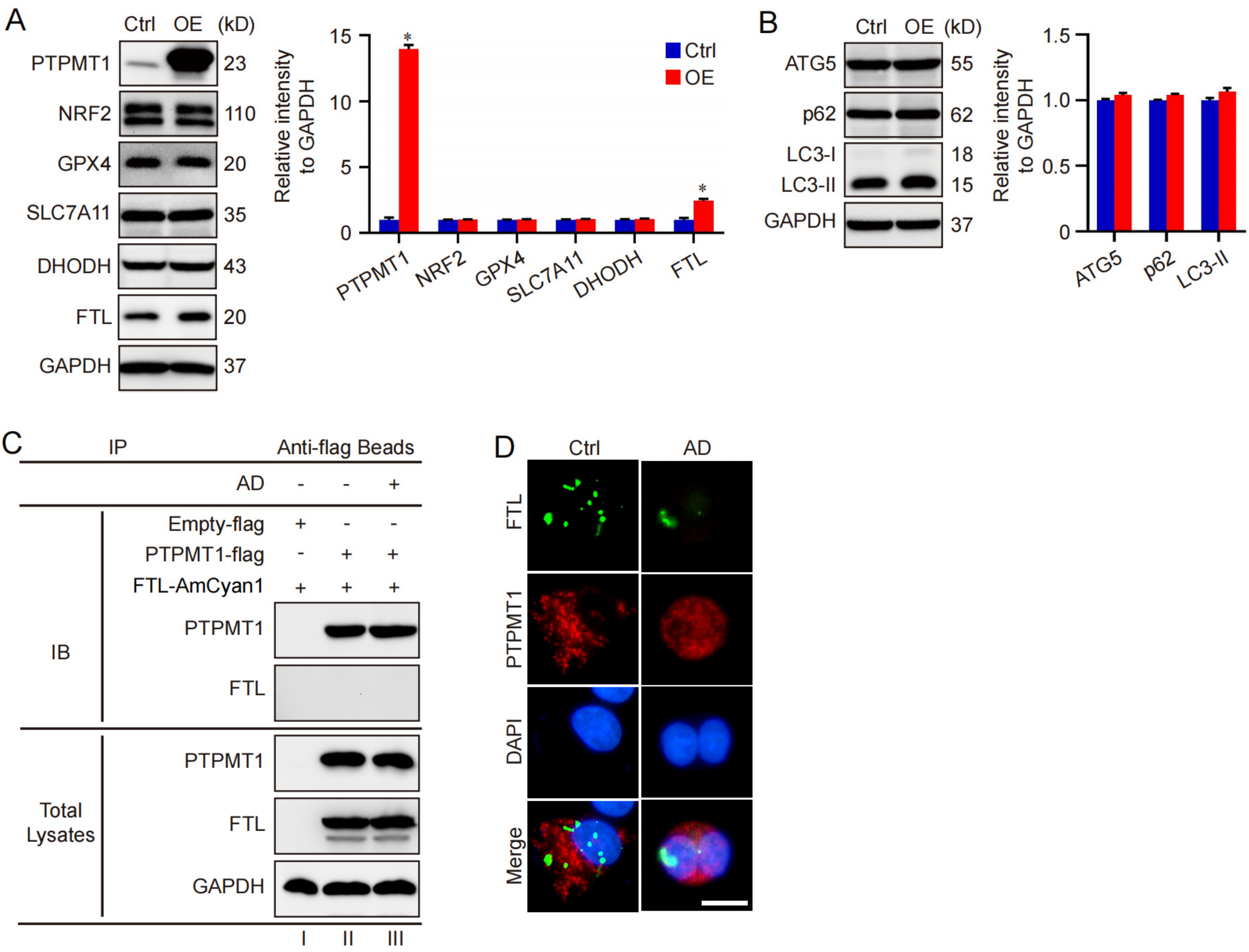

Figure S2. The effect of PTPMT1 overexpression on expression of ferroptosis regulators and autophagy makers. Hep 3B cells were transfected with a PTPMT1 overexpression vector for a duration of 24 hours, with the empty vector serving as the control (Ctrl) group. Subsequent to transfection, cell samples were collected for western blot analysis. Within this context, the protein expression levels of various ferroptosis regulators (A) and autophagy markers (B) were ascertained. Results are expressed as mean ± SD, and the P value less than 0.05 was considered statistically significant. *:P < 0.05 compared between two groups. To confirm the interaction between PTPMT1 and FTL, cells transfected with Flag-tagged PTPMT1 and Amcyan 1-tagged FTL were treated with AD (1 μM) for 4 hours. The binding between PTPMT1 and FTL was evaluated through immunoprecipitation assay (C). In addition, the effect of treatment with AD (1 μM) for 24 hours on the co-localization of

PTPMT1 and FTL was determined via immunofluorescence staining (D).

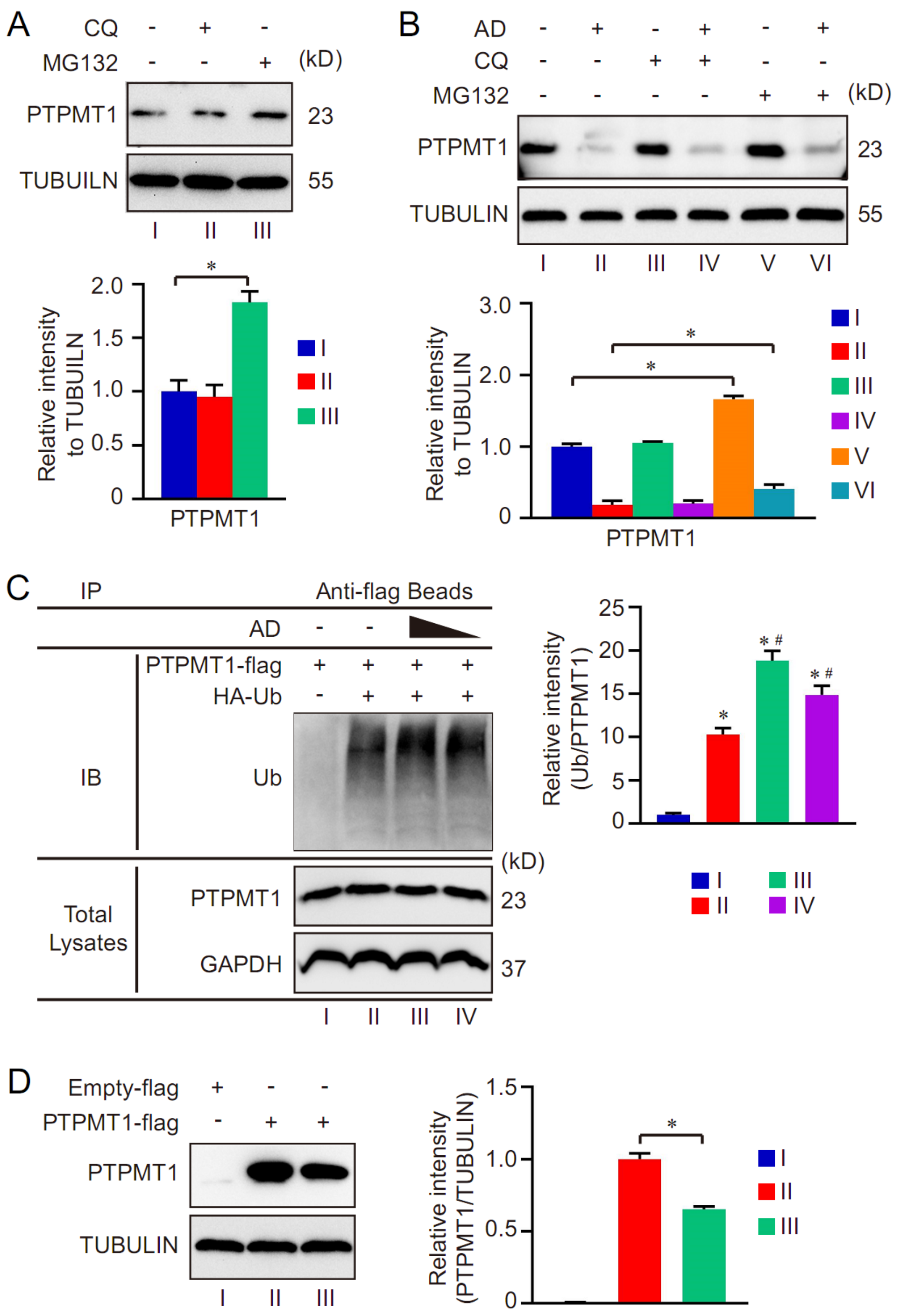

Figure S3. AD treatment augments the proteasomal degradation of PTPMT1 in HCC cells. Hep 3B cells were administered MG132 (5 μM) and Chloroquine (CQ, 20 μM) for 24 hours, followed by harvesting for a PTPMT1 assay (A). Additionally, cells co-treated with AD (1 μM) and CQ/MG132 for 24 hours were also harvested, and the protein levels of PTPMT1 were assessed via western blot (B). Besides, the cells were transfected with Flag-tagged

PTPMT1 and HA-tagged Ub were treated with AD (1 and 2 μM) and MG132 (5 μM) for 4 hours. The ubiquitination-induced degradation of PTPMT1 was evaluated via immunoprecipitation assay (C, Group I-IV represent four different conditions in left panel). In addition, the effect of AD treatment (1 μM for 8 hours) on PTPMT1 vector transfection was further evaluated using immunoblot (D, Group I-III represent three different conditions in left panel). Results are expressed as mean ± SD, and the P value less than 0.05 was considered statistically significant. In Figure S3A-S3B and S3D, *:$P < 0.05$ compared between two groups. In Figure S3C, *:$P < 0.05$ compared with group I, and #:$P < 0.05$ compared with group II.

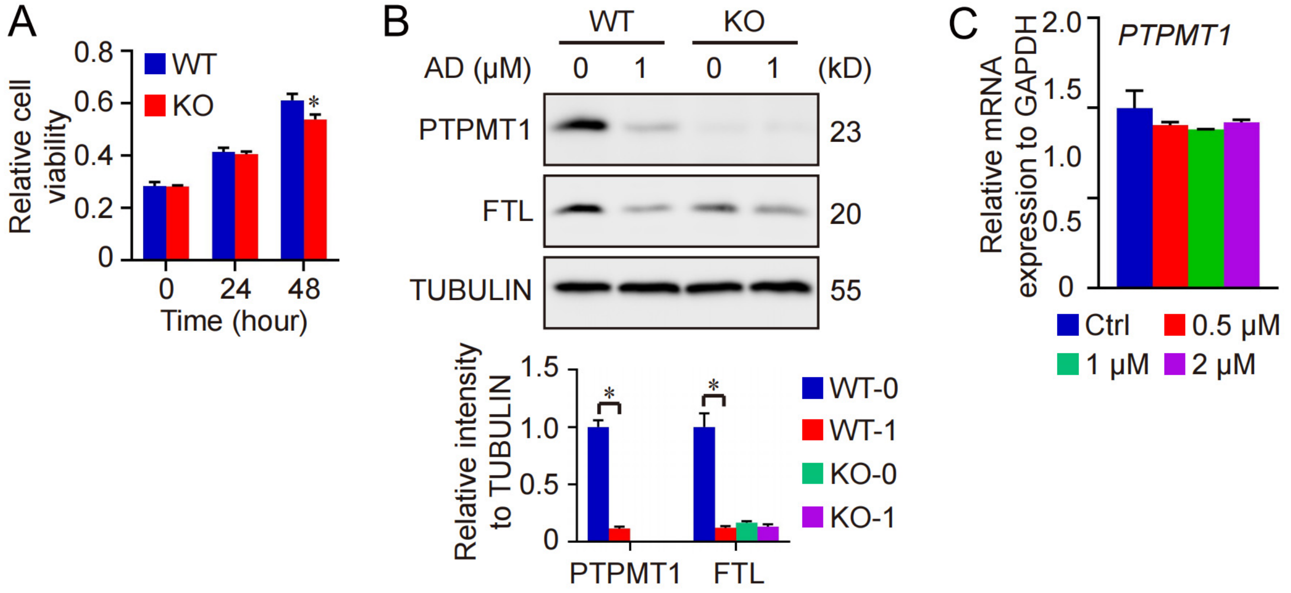

Figure S4. Effect of PTPMT1 knockout on HCC cell viability and AD function. The cell viability of wild type HCC cells and PTPMT1 knockout HCC cells were evaluated using CCK-8 first (A). The impact of AD (1 μM for 24 hours) on both wild-type and PTPMT1- knockout cells was investigated in this study. The protein levels of PTPMT1 and FTL were assessed via western blot (B). Finally, the effect of AD treatment on PTPMT1 transcription was further determined using qRT-PCR (D). Data are presented as mean ± SD, with a P value less than 0.05 deemed statistically significant. *: $P < 0.05$ indicates a significant difference between the two groups.

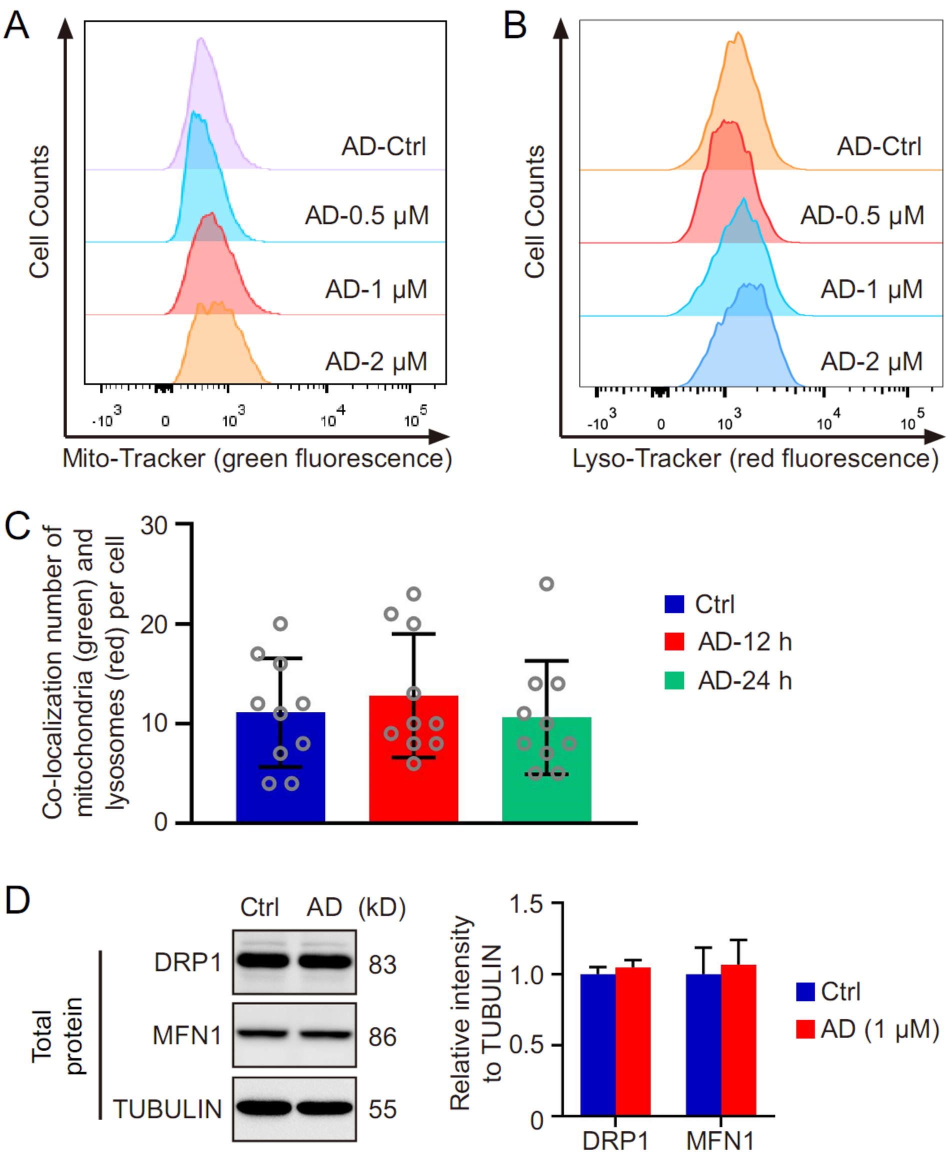

Figure S5. AD treatment does not exert an influence on the abundance of mitochondria and lysosomes. Hep 3B cells were exposed to varying concentrations of AD (0, 0.5, 1, and 2 μM) for a duration of 24 hours. Subsequently, the quantification of mitochondria and lysosomes was accomplished through the application of Mito-Tracker Green staining (A) and Lyso-Tracker Red (B) staining, followed by FACS assay. The staining results were observed under confocal microscope and the co-localization number of mitochondria (green) and lysosomes (red) per cell was quantified in our work (C). Immunoblot assay of total DRP and MFN1 in cytoplasm was performed using Hep 3B cells treated with AD (1 μM) for 24 hours (D).

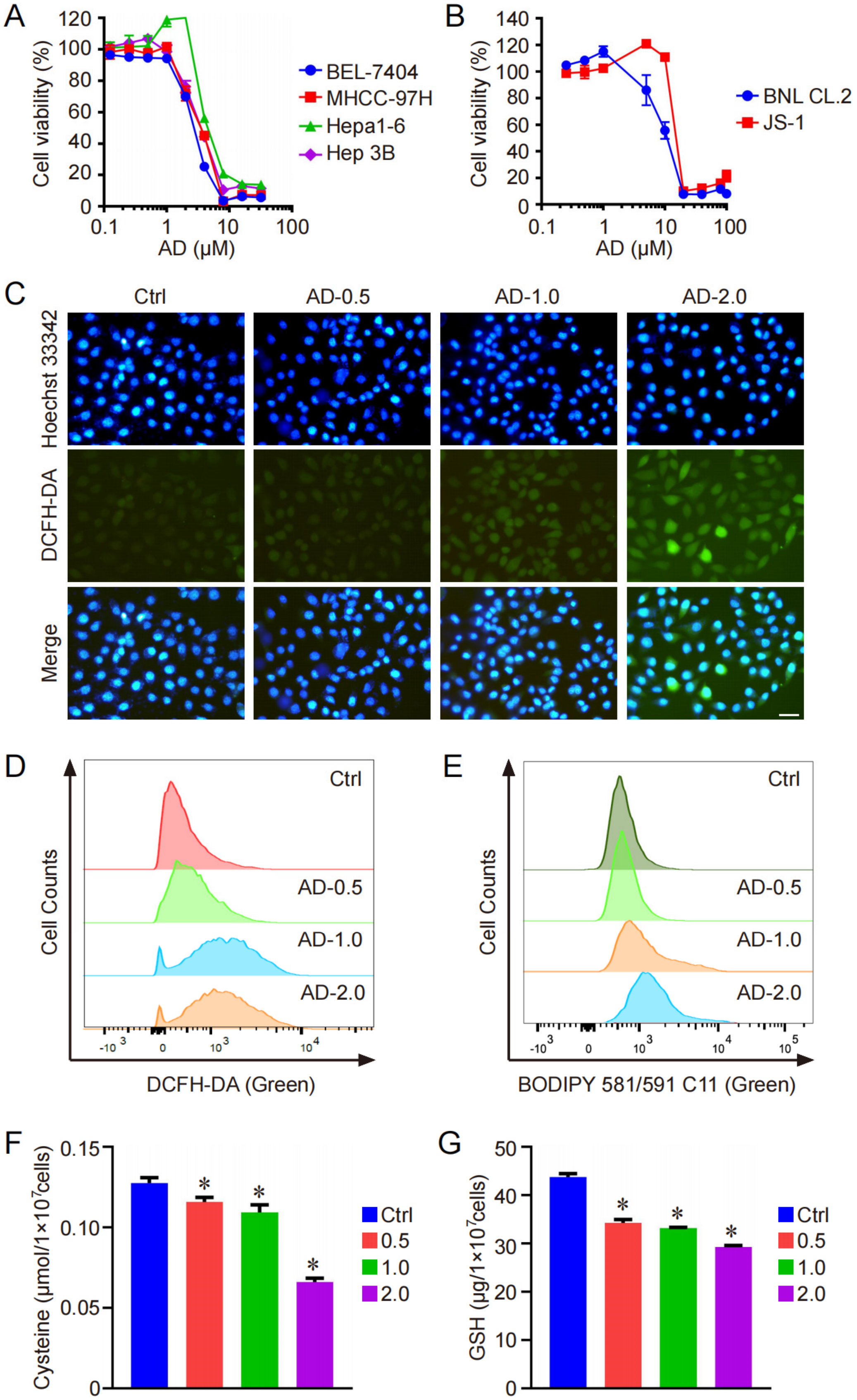

Figure S6. AD induces ferroptosis in HCC cells directly. AD directly triggers ferroptosis in

HCC cells. Various HCC cell lines (A) and non-cancerous cells, including normal liver cells (BNL CL.2) and hepatic stellate cells (JS-1) (B), were exposed to graded concentrations of AD for 24 hours. Subsequently, cell viability across each group was assessed using the CCK-8 assay. Hep 3B cells, subjected to AD treatments at concentrations of 0, 0.5, 1, and 2 μM for 24 hours, were harvested for the ROS generation assay, employing DCFH-DA staining (C-D), and the lipid peroxidation levels (E) were quantified using BODIPY staining to ascertain the ferroptotic response in AD-treated HCC cells. Scale bar = 25 μm. Ultimately, the levels of cysteine (F) as well as GSH (G) in AD-treated cells were tested respectively. Data are presented as mean ± SD, with a P value less than 0.05 deemed statistically significant. *: $P < 0.05$ indicates a significant difference compared with Ctrl group.

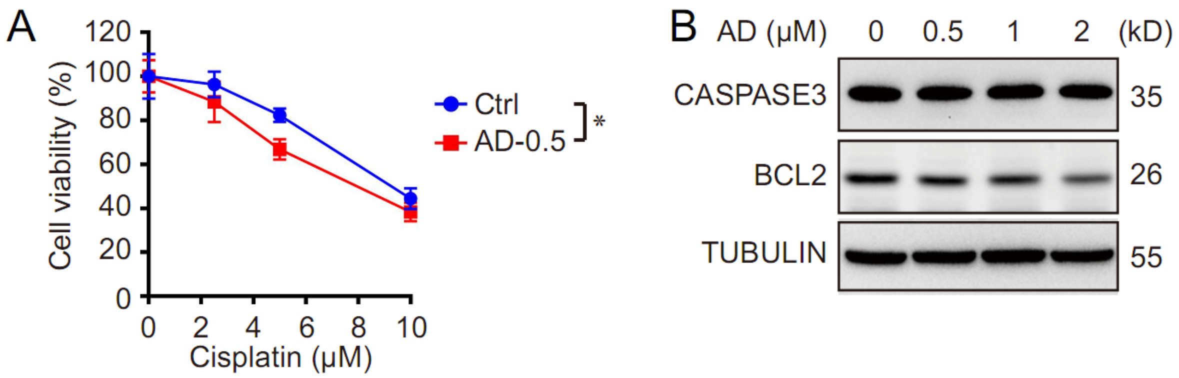

Figure S7. Effect of AD on HCC cell apoptosis. Hep 3B cells underwent co-treatment with AD and Cisplatin for 24 hours. Subsequently, cell viability across each group was assessed using the CCK-8 assay (A). Data are presented as mean ± SD, with a P value less than 0.05 deemed statistically significant. *: $P < 0.05$ indicates a significant difference between the two groups. Additionally, Hep 3B cells, exposed to AD at concentrations of 0, 0.5, 1, and 2 μM for 24 hours, were harvested for western blot analysis (B).
